# The Development of the Davis Food Glycopedia—A Glycan Encyclopedia of Food

**DOI:** 10.3390/nu14081639

**Published:** 2022-04-14

**Authors:** Juan J. Castillo, Garret Couture, Nikita P. Bacalzo, Ye Chen, Elizabeth L. Chin, Sarah E. Blecksmith, Yasmine Y. Bouzid, Yael Vainberg, Chad Masarweh, Qingwen Zhou, Jennifer T. Smilowitz, J. Bruce German, David A. Mills, Danielle G. Lemay, Carlito B. Lebrilla

**Affiliations:** 1 Department of Chemistry, University of California Davis, Davis, CA 95616, USA; jjcastillo@ucdavis.edu (J.J.C.); gacouture@ucdavis.edu (G.C.); nbacalzo@ucdavis.edu (N.P.B.J.); yapchen@ucdavis.edu (Y.C.); qwzzhou@ucdavis.edu (Q.Z.); 2 Foods for Health Institute, University of California Davis, Davis, CA 95616, USA; yvainberg@ucdavis.edu (Y.V.); cfmasarweh@ucdavis.edu (C.M.); jensm@ucdavis.edu (J.T.S.); jbgerman@ucdavis.edu (J.B.G.); damills@ucdavis.edu (D.A.M.); 3 Western Human Nutrition Research Center, USDA Agricultural Research Service, Davis, CA 95616, USA; elizabeth.chin@usda.gov (E.L.C.); danielle.lemay@usda.gov (D.G.L.); 4 Department of Nutrition, University of California Davis, Davis, CA 95616, USA; sblecksmith@ucdavis.edu (S.E.B.); yybouzid@ucdavis.edu (Y.Y.B.); 5 Department of Food Science and Technology, University of California Davis, Davis, CA 95616, USA; 6 Department of Viticulture and Enology, University of California Davis, Davis, CA 95616, USA; 7 Department of Biochemistry and Molecular Medicine, University of California Davis, Davis, CA 95616, USA

**Keywords:** complementary foods, monosaccharide, polysaccharide, food composition, diet, dietary carbohydrates, fiber, library, microbiome, triple quadrupole mass spectrometry

## Abstract

The molecular complexity of the carbohydrates consumed by humans has been deceptively oversimplified due to a lack of analytical methods that possess the throughput, sensitivity, and resolution required to provide quantitative structural information. However, such information is becoming an integral part of understanding how specific glycan structures impact health through their interaction with the gut microbiome and host physiology. This work presents a detailed catalogue of the glycans present in complementary foods commonly consumed by toddlers during weaning and foods commonly consumed by American adults. The monosaccharide compositions of over 800 foods from diverse food groups including Fruits, Vegetables, Grain Products, Beans, Peas, Other Legumes, Nuts, Seeds; Sugars, Sweets and Beverages; Animal Products, and more were obtained and used to construct the “Davis Food Glycopedia” (DFG), an open-access database that provides quantitative structural information on the carbohydrates in food. While many foods within the same group possessed similar compositions, hierarchical clustering analysis revealed similarities between different groups as well. Such a Glycopedia can be used to formulate diets rich in specific monosaccharide residues to provide a more targeted modulation of the gut microbiome, thereby opening the door for a new class of prophylactic or therapeutic diets.

## 1. Introduction

Carbohydrates make up the largest component of human diets, comprising up to 85% depending on geographic location and socioeconomic status [1]. These biomolecules play a profound role in shaping our gut microbial communities, the spectrum of microbial metabolites produced, and the resulting impact on our health. For example, in early life, human milk oligosaccharides (HMOs) play a large role in feeding select *Bifidobacterium* species, thereby shaping the infant’s microbial communities and providing health benefits such as priming the immune system, strengthening the gut barrier, and blocking pathogens [2]. In adults, a high fat/high carbohydrate or “Western” diet has long been implicated in a variety of metabolic diseases such as cardiovascular diseases, type 2 diabetes, obesity, and gastrointestinal disorders [3,4]. On the other hand, consumption of plant-based foods is associated with reducing the risks of those metabolic diseases [5].

Recent research has emphasized the importance of the gut microbiome in the nutrition-health paradigm. Specifically, dietary carbohydrates modulate human health through their interaction with the gut microbiome [6,7]. Despite their importance and the fact that they are one of the most abundant components in foods, their structures, abundances, and functions are still poorly characterized due to a general lack of appropriate analytical methods [8]. While the analysis of proteins and lipids have advanced greatly, the analysis of carbohydrates has been hindered by the inherent complexity of carbohydrate structures. Current groupings categorize carbohydrates into the broad classifications of sugars, starch, and soluble/insoluble fiber; terms which provide little information on specific chemical or structural content therein. Indeed, the common term “fiber” offers no monosaccharide or structural specificity, yet is regularly employed to represent various heterologous polysaccharides composed of differing sugar and linkage assemblages. Perhaps most revealing, total carbohydrates in most foods are currently measured indirectly by gravimetric mass difference of other macronutrients and micronutrients thereby depicting a form of nutritional “dark matter” [9]. Such a lack of chemical resolution impedes efforts to resolve the relationships between carbohydrates, the gut microbiome, and host health. There is thus a need for rapid throughput methods that are capable of characterizing carbohydrate structures and their microbiome interactions in large feeding studies [10].

Food carbohydrates are comprised of a diverse set of molecules ranging from free monosaccharides, disaccharides, oligosaccharides, and large polysaccharides. Additionally, each monosaccharide residue connects to another through numerous linkages (as many as 10 for each glycosidic linkage). Methods for oligosaccharide analysis using liquid chromatography-tandem mass spectrometry (LC-MS/MS) have been developed for structural elucidation, however the analyses remain very difficult and require a number of separation steps and structural elucidation techniques [8]. Furthermore, even the most fundamental information, the monosaccharide composition, is not known in most foods. The lack of this basic structural information inhibits our understanding of the role of the most abundant material in our diet. It prevents the effective design of important clinical trials that could elucidate the specific roles of specific carbohydrate structures in food.

In this work, a recently developed workflow utilizing a high-throughput UPLC-QqQ-MS method was employed to determine the monosaccharide compositions of over 800 food samples. Foods from diverse groups such as fruits, vegetables, fats, grains, dairy, beverages, and processed foods were subjected to monosaccharide analysis, and the resulting monosaccharide compositions were used to create a foundational collection of compositions in a resource here named the Davis Food Glycopedia (DFG) for “Glycan Encyclopedia.” The method entailed the absolute quantitation of 14 naturally occurring monosaccharides separated on a five-minute UPLC-QqQ MS analysis in a 96-well plate format. The monosaccharide compositions of foods within and between food groups, as individual foods, and as part of a diet were revealed. This platform and the resulting Glycopedia will allow for the formulation of feeding trials where the diets may be highly enriched for specific monosaccharide compositions. Tailoring diets will enable future studies to better understand the role of food carbohydrates in shaping the gut microbiome in infants and adults. Furthermore, the presented findings will allow for dietary interventions that are more precisely formulated for modulating the gut microbiome and impacting human health.

## 2. Materials and Methods

### 2.1. Selection of Foods for Inclusion in the Glycopedia

Foods were initially selected for the Glycopedia to design a feeding trial of fiber-rich foods to selectively enrich beneficial gut microbiota in toddlers (12–36 months). The toddler foods selected for the DFG included single foods recommended for toddlers according to the 2020–2025 Dietary Guidelines for Americans [11], which included a diverse group of vegetables such as dark and green vegetables; red and orange vegetables; beans, peas, and lentils; in addition to fruits and starches. The DFG also includes food mixtures and snacks that contain various levels of carbohydrates. Additional foods were then selected to cover foods that are commonly consumed by adults.

To determine foods commonly consumed by adults, three datasets were reviewed: (1) the Nutritional Phenotyping study (NutPheno) [12]; (2) What We Eat in America (WWEIA) 2017–2018 [13]; and (3) the Food and Nutrient Database for Dietary Studies Ingredients Database (FNDDS-Ing) [14]. The NutPheno study was a cross-sectional study that included healthy male and female adults, aged 18–66 y, living near Davis, CA. The NutPheno study included 393 adult subjects who reported dietary intake with up to 4 days of 24-h recalls using the Automated Self-Administered 24-h Dietary Assessment Tool (ASA24) [15]. WWEIA is the dietary component of the National Health and Nutrition Examination Survey (NHANES), a nationally-representative cross sectional study and consists of two 24-h dietary recalls. Both the WWEIA dietary assessment and ASA24 use the Food and Nutrient Database for Dietary Studies (FNDDS), and the food descriptions and numeric identifiers (Food Code) come from FNDDS.

In the NutPheno study, a total of 2435 unique foods (corresponding to 2435 unique FNDDS Food Codes) were reported from a total of 1499 recalls. To identify candidate adult foods to add to the Glycopedia from the NutPheno study, the frequency of each food reported in NutPheno was counted, and the 200 most frequently reported foods were manually cross-matched by searching the food description in the Glycopedia for the closest match. Of the top 200 most frequently consumed foods, 135 did not have a matching Glycopedia food. A second round of manual curation was conducted on these 135 NutPheno foods to identify candidate foods to add to the Glycopedia (e.g., would they be likely to contribute to dietary glycan consumption and/or are they typically consumed in large quantities or very frequently, *n* = 59). 

The same process described above for NutPheno foods was used for FNDDS-Ing and WWEIA. A total of 2744 unique ingredients were identified in FNDDS-Ing. The frequency of an ingredient corresponds to the total number of times the ingredient is used in FNDDS recipes. Of the top 200 most frequently reported ingredients, 137 did not have matches to the Glycopedia, 79 of which were considered as candidates to add to the Glycopedia. A total of 7083 foods were reported in WWEIA. Of the top 200 most frequently consumed foods, 130 had no Glycopedia match, and 49 were considered as candidates to add to the Glycopedia. 

### 2.2. Sources of Materials

All foods and food products were purchased from local markets (Davis and Sacramento, CA, USA) including Safeway, Trader Joe’s, Davis Food Co-op, Whole Foods, Nugget Markets, Target, and online (Amazon). Trifluoroacetic acid (TFA, HPLC grade), 3-methyl-1-phenyl-2-pyrazoline-5-one (PMP), chloroform (HPLC grade), ammonium hydroxide solution (NH_4_OH) (28–30%), ammonium acetate, sodium acetate, glacial acetic acid, methanol (HPLC grade), D-fructose, D-mannose, D-allose, D-glucose, D-galactose, L-rhamnose, L-fucose, D-ribose, D-xylose, L-arabinose, N-acetyl-D-glucosamine (GlcNAc), N-acetyl-D-galactosamine (GalNAc), D-glucuronic acid (GlcA), and D-galacturonic acid (GalA) were purchased from Sigma-Aldrich (St. Louis, MO, USA). Arabinoxylan and polygalacturonic acid were purchased from Megazyme (Bray, Ireland). 96-well Nunc plates and lids were purchased from Thermo Scientific. Viscozyme was provided by Novozyme (Davis, CA, USA). Acetonitrile (ACN) (HPLC grade) was purchased from Honeywell (Muskegon, MI, USA). Nanopure water was used for all experiments.

### 2.3. Preparation of Food and Quality Control (QC) Samples

A total of 828 foods including fresh, frozen, commercial, and processed were purchased from local grocery stores in Davis, CA, USA. Each food was documented with detailed descriptions prior to the sample preparation. Many foods were aliquoted raw for analysis. For some raw and packed foods, samples were first cooked, baked, or steamed as indicated on the package for cooking instructions. Foods were lyophilized to complete dryness and the moisture content was obtained. Samples underwent a dry bead blast or mortar and pestle for homogenization. A 10 mg aliquot of dried food sample was weighed into a 1.5 mL screw cap Eppendorf tube and reconstituted with water to make a stock solution of 10 mg/mL. The stock solution then underwent a bullet blending procedure followed by heat treatment (1 h at 100 °C) and another round of bullet blending prior to monosaccharide analysis. Arabinoxylan and polygalacturonic acid polysaccharide standards were used as QC samples and were prepared in 10 mg/mL stock solutions using the same bullet blending and incubation protocol as food samples.

### 2.4. Monosaccharide Analysis of Food Samples

The monosaccharide analysis of foods was adapted from Xu et al. [16] and Amicucci et al. [17] with the following modifications. A 10 µL aliquot from the homogenized sample or QC stock solution was subjected to incubation with Viscozyme treatment at 50 °C for 1 h in 390 µL of 25 mM acetate buffer (pH 5). A 100 µL aliquot from the enzyme digest was subjected to hard acid hydrolysis with 4 M TFA for 1 h at 121 °C and quenched with 855 µL of ice-cold water. A pool of monosaccharide standards consisting of D-fructose, D-mannose, D-allose, D-glucose, D-galactose, L-rhamnose, L-fucose, D-ribose, D-xylose, L-arabinose, D-GlcNAc, D-GalNAc, D-GlcA, and D-GalA were used to generate a calibration curve and were prepared in water ranging in concentration from 0.001 to 100 µg/mL. The released monosaccharides in samples and standards were then derivatized with 0.2 M PMP solution in methanol and 28% NH_4_OH at 70 °C for 30 min. Samples were then dried to completeness by vacuum centrifugation. The excess PMP was removed by a chloroform extraction and a 1 µL aliquot of the derivatized monosaccharides were subjected to UPLC-QqQ MS analysis.

### 2.5. Mass Spectrometry Instrumental Analysis

Derivatized glycosides were separated on an Agilent Poroshell HPH-C18 column (2.1 × 50 mm, 1.9 µm) and guard using an Agilent 1290 Infinity II UPLC system. A constant flow rate of 1.050 mL/min was employed on a 2 min isocratic elution at 12% solvent B followed by a 1.6 min flush at 99% solvent B and 0.79 min equilibration for a total run time of 4.6 min for the separation of compounds. Solvent A consisted of 25 mM ammonium acetate in 5% acetonitrile with pH adjusted to 8.2 using concentrated ammonia solution. Solvent B consisted of 95% acetonitrile in water. The separated glycosides were then detected on an Agilent 6495B triple-quadrupole mass spectrometer (QqQ-MS) operated in positive ion mode using dynamic multiple reaction monitoring (dMRM). 

### 2.6. Data Analysis

Raw LC-MS files were analyzed using Agilent MassHunter Quantitative Analysis software (Version B 08.00). Chromatographic peaks were manually integrated and matched with standards. Monosaccharides were quantified by external calibration curve fitted with linear regression. Clustering analysis based on monosaccharide profiles were conducted with R using the circlize library (v 0.4.13). Dendrograms and heatmaps used to visualize the clustered data were also generated using circlize. The enrichment of food groups in each cluster was determined using a hypergeometric test and statistical significance was assigned based on FDR-adjusted *p*-values. 

### 2.7. Assigning Food Groups to Glycopedia Foods

The DFG food groups are adapted from the FNDDS food groups that are defined by the first two digits of the FNDDS Food Code: [18] (1) Milk and Milk Products, (2) Meat, Poultry, Fish, and Mixtures, (3) Eggs, (4) Beans, Peas, Other Legumes, Nuts, Seeds, (5) Grain Products, (6) Fruits, (7) Vegetables, (8) Fats, Oils, and Salad Dressings, and (9) Sugars, Sweets and Beverages (excluding juice and plant-based milks). Food groups were assigned based on a Glycopedia food’s first ingredient; the second ingredient was used if water was the first ingredient. For example, both mango juice and fresh yellow mango are Fruits, and orange preserves (first ingredient is sugar) is in Sugars, Sweets, and Beverages. When the ingredient labels for multi-ingredient foods could not be found online, the food group was assigned based on the product name and description. 

## 3. Results

We employed a recently developed LC-MS platform to quantitate the monosaccharide compositions of over 800 foods. The collection included whole and processed foods with an emphasis on the earliest complementary (weaning) foods and common adult foods typical of diets among the US population. The resulting glycan compositions of the foods were used in the clustering analysis to identify food groups with common or similar monosaccharide characteristics. The DFG database is publicly available via GitHub and will be iteratively improved as more information is obtained such glycosidic linkage compositions and free saccharides. The DFG can be further used to create diets enriched in specific monosaccharides for observational and interventional feeding trials that would probe for host-microbe interactions.

### 3.1. Monosaccharide Compositional Analysis of Foods

Foods purchased in local markets were documented with detailed descriptions and processed using preparation procedures that included cooking (where applicable), lyophilization, and homogenization. Moisture contents were determined with the lyophilization step. The samples were first digested with Viscozyme, a multi-enzyme mixture containing strong pectolytic activity, to target the acid-recalcitrant α1→4 GalA bonds found in pectins. Digested samples were then subjected to acid hydrolysis and chemically-labeled to enhance MS ionization and facilitate chromatographic separation in a five-minute UPLC-QqQ-MS analysis. The absolute monosaccharide abundances were obtained using dynamic multiple reaction monitoring (dMRM) and standard monosaccharide solutions were analyzed to generate external calibration curves. Chromatograms of a pooled monosaccharide standard and some selected foods are shown in Supplementary Figure S1. Arabinoxylan and polygalacturonic acid polysaccharide standards were used as QCs for each batch of samples. A control chart summarizing the measured concentrations of the primary monosaccharides detected in each standard is depicted in Supplementary Figure S2. All values measured in each standard fell within two standard deviations of the mean.

**Figure 1:**
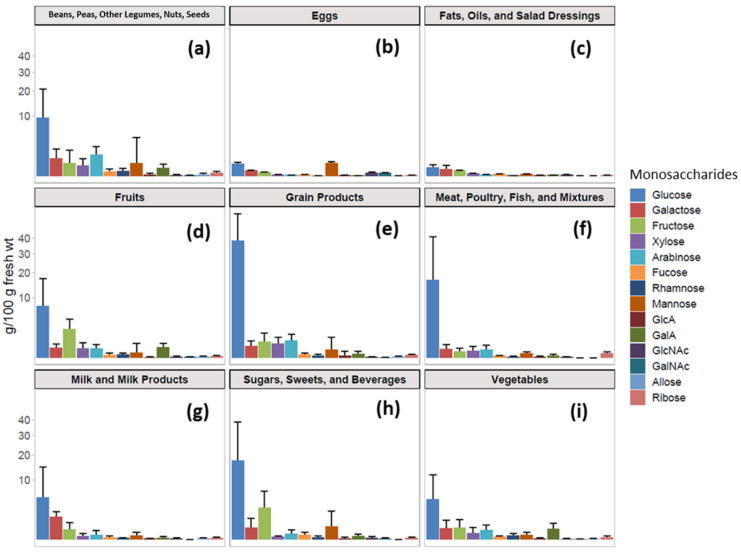
(**a**–**i**) Average monosaccharide compositions of all nine food groups. The *y*-axis follows a square root scale. Error bars represent the standard deviation.

Foods are traditionally assigned to groups and the largest groups in this study were those classified as Fruits, Vegetables, Grain products, Beans, Peas, Other Legumes, Nuts, Seeds (Supplementary Figure S3). Other groups including Meat, Poultry, Fish and Mixtures, Eggs, Milk and Milk Products, Fats, Oils, and Salad Dressings, and Sugars, Sweets, Beverages were also included, although they contained fewer entries. The 14 monosaccharides monitored included glucose, galactose, fructose, xylose, arabinose, fucose, rhamnose, mannose, GlcA, GalA, GlcNAc, GalNAc, allose, and ribose. All were found in measurable abundances except allose, GlcNAc, and GalNAc. Due to the harsh nature of the acid-catalyzed depolymerization of food samples, fructose degraded more readily than the others. Fructose degraded the most and for the disaccharide sucrose (table sugar), it degraded by as much as 90%. The reported value takes this degradation into consideration. Average abundances of monosaccharides were calculated for the food groups (Figure 1a–i). The most commonly found and often the most abundant monosaccharide was glucose likely from starch and/or sucrose. Other common and abundant monosaccharides, particularly those from plant-based foods were fructose, xylose, arabinose, galactose, and GalA with all but fructose likely due to cell wall polysaccharides such as arabinoxylan and pectins [19,20]. Xylose and arabinose were most abundant in Grain Products (Figure 1e) due to the presence of arabinoxylans in their cell walls, while GalA and rhamnose were common in Beans, Peas, Other Legumes, Nuts, Seeds, Fruits, and Vegetables (Figure 1a,d,i) likely due to the abundance of pectins [19,20]. Grain Products had the highest overall measured carbohydrates by fresh weight (Figure 1e) due to high starch and low moisture content followed by Beans, Peas, Other Legumes, Nuts, Seeds, Fruits, and Vegetables (Figure 1a). Eggs and Fats, Oils, and Salad Dressings (Figure 1b,c, respectively) were found to have the lowest carbohydrate content. Meat, Poultry, Fish and Mixtures group contained significant amounts of glucose largely due to bread coatings of meats such as those found in breaded chicken and fish (Figure 1f). Additionally, soups which are in this group also contain large amounts of glucose. However, the analysis of unprocessed meat, poultry, and fish revealed very little glucose. 

**Figure 2:**
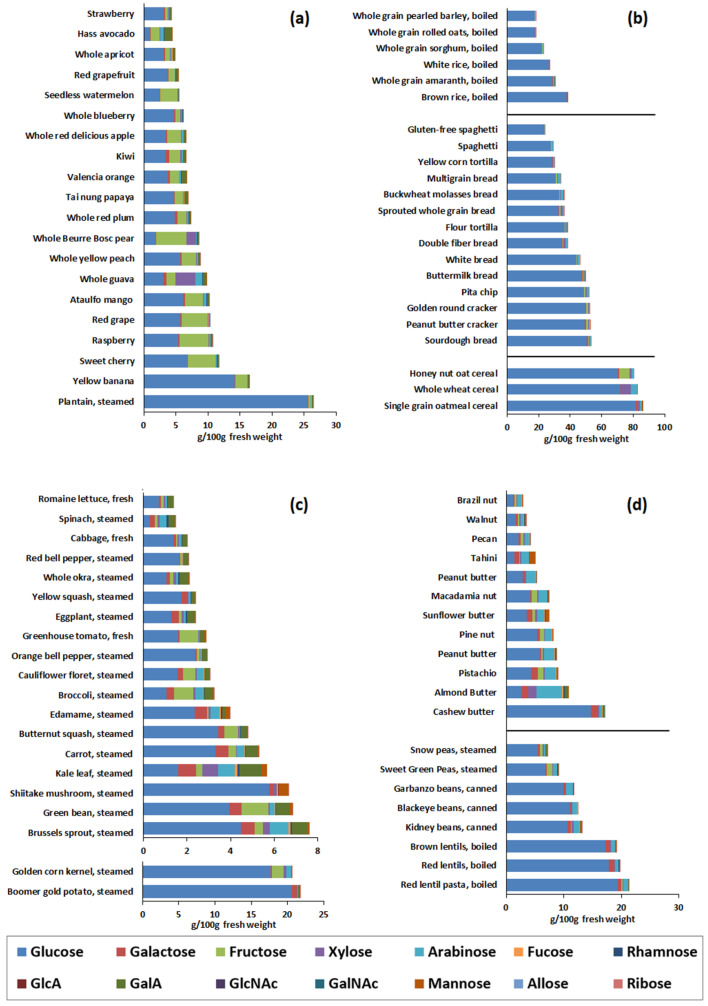
(**a**–**d**). Monosaccharide compositions of selected representative foods from each plant-based food group for (**a**) Fruits, (**b**) Grains Products, (**c**) Vegetables, and (**d**) Beans, Peas, Other Legumes, Nuts, Seeds.

The traditional method of grouping foods misrepresents the carbohydrate content. When foods from plant-based groups are analyzed, each entry had markedly different monosaccharide compositions. Figure 2a–d depicts the monosaccharide compositions of 20 representative foods from each of the plant-based food groups, which include Fruits, Grain Products, Vegetables, and Beans, Peas, Other Legumes, Nuts, Seeds. Even the Fruits group exhibited diverse monosaccharide compositions, although it tended to contain significantly more fructose than other groups, as expected (Figure 2a). Grain Products (Figure 2b) exhibited the highest glucose content from starch, but also contained xylose, arabinose, and galactose. In grains, “white” products like white bread, flour tortillas, and white rice tended to contain less non-glucose monosaccharides than their whole grain counterparts like whole-grain bread, grains, and brown rice (Figure 2b, Supplementary Figure S4).

Aside from potatoes and corn, which have high-starch contents, most vegetables (Figure 2c) had diverse monosaccharide compositions consisting of glucose, fructose, galactose, xylose, arabinose, GalA, and mannose and were more similar to fruits, but with markedly less fructose. Beans, Peas, Other Legumes, Nuts, Seeds (Figure 2d) contained relatively high amounts of arabinose. However, beans and peas had larger amounts of glucose than nuts due to higher starch content.

Solanaceous foods (or nightshades) like tomatoes, eggplant, and bell peppers contained very little arabinose and non-glucose monosaccharides while members of the Brassicaceae family like brussels sprouts, broccoli, kale, and cauliflower yielded significantly larger quantities of arabinose and other monosaccharides such as GalA, galactose, rhamnose, and fucose (Figure 2c). In nuts, almonds contained the largest amount of arabinose while tahini (made from sesame seeds) contained large amounts of mannose (Figure 2d). In fruits, pears and guava tended to contain more xylose than other fruits while berries were very low in non-glucose and non-fructose monosaccharides (Figure 2a). 

Several of the same foods were also purchased and analyzed fresh or frozen from multiple producers/brands. For example, spinach was analyzed from various sources as fresh, frozen, and steamed (Supplementary Figure S5). While the absolute abundance of each monosaccharide differed (less than 30% for most of the abundant monosaccharides), the compositions remained consistent with galacturonic acid, arabinose, galactose, glucose, rhamnose, and xylose being the major monosaccharides.

### 3.2. Clustering Analysis Yields New Combinations of Foods

**Figure 3:**
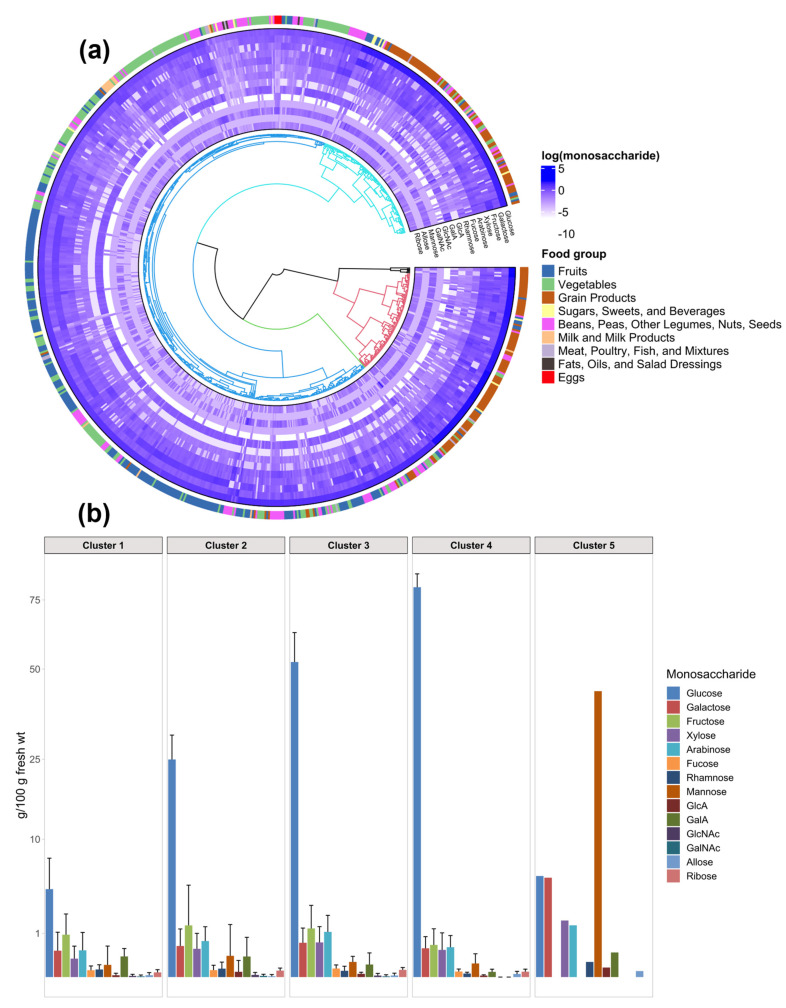
(**a**) Hierarchical cluster analysis of all 828 foods based on their absolute monosaccharide compositions, (**b**) Average monosaccharide composition of each cluster. The *y*-axis follows a square root scale. Error bars represent the standard deviation.

To visualize common features and differences among individual monosaccharide compositions irrespective of food group, an unsupervised hierarchical clustering analysis of the DFG was performed (Figure 3a). The average monosaccharide compositions of each cluster are depicted in Figure 3b. A total of five clusters were used to divide the 828 foods into clusters based on their total monosaccharide compositions (Figure 3a). The number of clusters chosen was based on various clustering indices [21]. Food belonging to the same groups (Fruits, Vegetables, and Grain Products) were largely clustered together as defined by their monosaccharide compositions, however these classifications were essentially dominated by the amount of glucose. Cluster enrichment factors were included in Supplementary Figure S6. 

Glucose was the most abundant component in many of the samples, and hence was the major factor for the separation of the clusters. Cluster 1 was the largest, comprising of over half of the total foods surveyed. This cluster was significantly enriched in Fruits, Vegetables, and the Beans, Peas, Other Legumes, Nuts, Seeds group, but also contained entries from all of the other food groups. We further separated Cluster 1 and obtained sub-clusters as shown in Supplementary Figure S7. Based on this sub-clustering analysis, specific groupings were observed such as Cluster 1A (intermediate glucose and fructose) with apples and toddler food products, Cluster 1B with stone fruits, tomatoes, berries, squash, fruit juices, and soups, and Cluster 1C (high glucose) with potatoes, bananas, and oats. Cluster 1F comprised of almond butters and flax seed with high arabinose and xylose values, while Clusters 1G (soy flour, roasted seaweed) and 1H (coffee grounds, dried coconut chips) are high in galactose and mannose, respectively. The average monosaccharide composition for Cluster 1 as a whole was most dissimilar to Clusters 2–5 (Figure 3b) and largely reflected the Fruit and Vegetable food groups. Specifically, Cluster 1 contained significantly lower amounts of glucose and a larger overall diversity than other clusters. Cluster 2 was significantly enriched in Grain Products, which were largely breads and cooked whole grains such as oats, barley, millet, quinoa, and rice. Additionally, Cluster 2 contained pastas, dried fruits, and plant-based meat products. Cluster 3 was significantly enriched in Grain Products, most of which were dried cereals and snacks. This cluster also contained foods from other groups such as Fruits and Vegetables, which were also mostly dried and snack products. Cluster 4 contained only six items, all of which were dried rice or corn products exhibiting the highest glucose and lowest non-glucose monosaccharides of all food analyzed. Cluster 5 is a single-member group with coconut flour having the highest amount of mannose in the DFG. 

### 3.3. Creation of Diets Based on Monosaccharide Compositions

In its current form, the DFG can be used to create fiber-focused diets. The utility of the DFG resource is that meals can be created with known amounts of carbohydrates based on monosaccharide compositions. The assembled DFG was then used to quantitate carbohydrates in a standard diet. According to the USDA Dietary Guidelines for Americans 2020–2025 and USDA MyPlate, it is recommended for adults to consume 2 cups of fruits, 2.5 cups of vegetables, 6 ounces of grains, 5.5 ounces of protein, and 3 cups of dairy in a day [11]. These recommendations are based on consuming 2000 calories per day and have different food groups compared to the food groups described in this work. To generate a relative chart of each food group (Supplementary Figure S8), the recommended servings in an example meal were converted from cups and ounces to grams. The ingredients for the example dinner meal included 4 ounces of chicken breast, 0.5 cups of broccoli, 0.33 cups of carrots, 0.33 cups of summer squash, 0.75 cups of pasta, 1 tablespoon of oil, 1 cup of a navel orange, and 1 cup of milk. Based on the USDA Dietary Guidelines for Americans 2020–2025, the recommended food groups relative composition for the example meal yielded 30% for vegetables, 26% for fruits, 19% for dairy, 13% for grains, and 12% for proteins.

**Figure 4:**
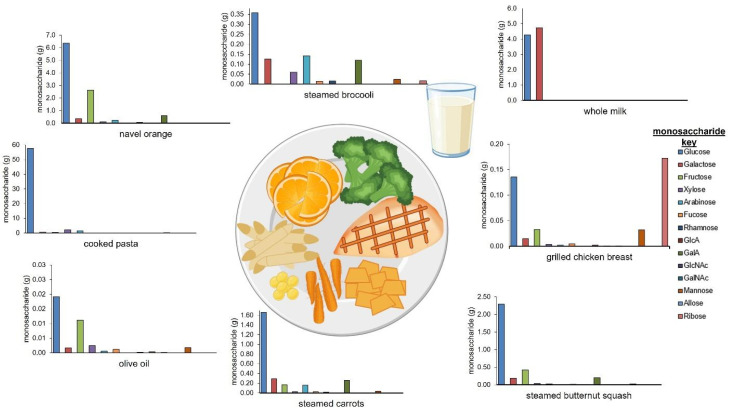
Example meal with quantitative monosaccharide bar graphs of each ingredient. The serving amounts are based on the USDA Dietary Guidelines for Americans 2020–2025.

The total dietary carbohydrate content in the example meal was determined using values from the DFG. Additionally, the monosaccharide concentrations and composition of each ingredient in the meal were determined (Figure 4). The calculated total carbohydrate content in the entire meal was 89.09 g (Table 1). The cooked penne pasta, navel orange, and glass of whole milk resulted in the highest total carbohydrate amounts (per ingredient and serving) with values of 62.4 g, 10.4 g, 9.1 g, respectively. As expected, olive oil, and grilled chicken breast had minimal carbohydrates (per ingredient and serving) with values 0.0 g, and 0.4 g, respectively. The cooked penne pasta had less relative monosaccharide diversity with glucose from starch as the most abundant. On the other hand, steamed broccoli, steamed carrots, navel orange and steamed butternut squash had the most (non-glucose) monosaccharide diversity with higher amounts of galactose, fructose, xylose, arabinose, and galacturonic acid present. Whole milk contained glucose (4.27 g) and galactose (4.74 g) per cup, which matches the known composition of lactose, the major disaccharide in milk.

In addition to determining the total carbohydrate content in each ingredient in a meal, the database was used to determine the total amount of each monosaccharide by adding the total monosaccharides from each ingredient. In the exemplified meal above, the glucose was the most abundant monosaccharide with a total of 72.70 g. The next most abundant monosaccharides were galactose and fructose with a total of 6.25 g and 3.72 g, respectively. Xylose (2.35 g) and arabinose (2.09 g) were similar in abundance, while fucose (0.09 g), rhamnose (0.13 g), galacturonic acid (1.21 g), mannose (0.30 g), and ribose (0.25 g) were present in smaller amounts. With the DFG, the monosaccharide profile of a meal can be altered by simply swapping an ingredient with another from the same food group with a higher concentration of the desired monosaccharide. For example, if more arabinose is desired, the navel orange (0.15 g/100 g arabinose) and cooked pasta (1.51 g/100 g arabinose) can be exchanged for a Bartlett pear (0.37 g/100 g arabinose) and sprouted wheat bread (2.11 g/100 g arabinose), respectively, to significantly and selectively increase the arabinose content of the meal.

### 3.4. Personalized Nutrition Based on Specific Monosaccharide Abundances

**Figure 5:**
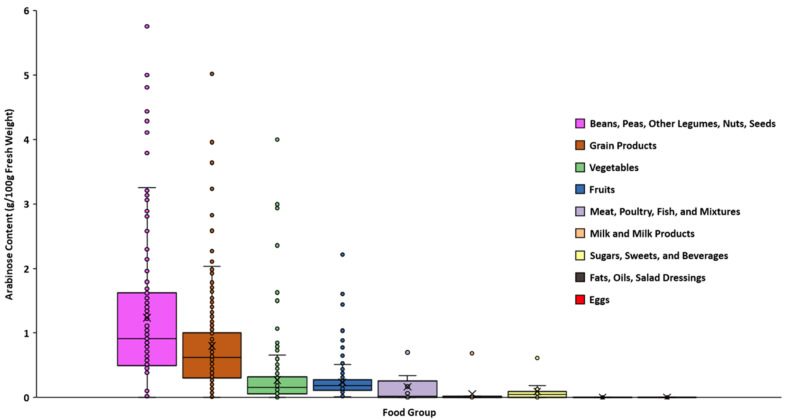
Average arabinose abundances of all food groups. Beans, Peas, Other Legumes, Nuts, Seeds yielded the highest arabinose amounts and animal-based groups like eggs yielded the least, respectively. The × indicates the average arabinose content while the solid line indicates the median.

The DFG can be used not only to compare foods, but to create personalized diets rich in specific monosaccharides, and by extension, fibers for altering and modulating the gut microbiome or other health endpoints. Arabinose is a prime target for this purpose as it is found commonly only in plants, is not digested or absorbed well endogenously in animal models, and has been shown to play an important role in shaping the gut microbiome [22,23,24]. Arabinose is not abundant in foods as a free monomer, rather it is a part of ubiquitous cell wall polysaccharides such as arabinoxylan in grains and pectins in fruits and vegetables [19,25]. While this method does not differentiate according to which polymer the arabinose originates, arabinose can nonetheless be quantitated to identify foods to maximize dietary levels of this monosaccharide. Figure 5 provides the broad arabinose content of the individual food groups. The highest average arabinose content was observed in Beans, Peas, Other Legumes, Nuts, Seeds (1.24/100 g fresh weight) followed by Grain Products, Vegetables, and Fruits (0.8, 0.27, and 0.24/100 g fresh weight). In general, the highest arabinose concentrations were found in plant-based foods such as legumes, grains, vegetables, and fruits. However, the range of arabinose in each plant-based food group was large and depended on the specific food and moisture content. For example, pear cultivars tended to have more arabinose than apple cultivars (Supplementary Figure S9). Relatively dry foods like cereals, nut butters, and dehydrated legume, vegetable, and fruit products consistently displayed the highest arabinose concentrations and total measured carbohydrate in each group (Supplementary Table S1). By selecting specific foods high in a particular monosaccharide such as arabinose, dietary intake of fibers containing that monosaccharide can be selectively increased, thereby providing an avenue for investigating the impact of particular fiber structures on the gut microbiome and health.

### 3.5. Processed Foods—Monosaccharide Composition in Commercial Complementary Foods

**Figure 6:**
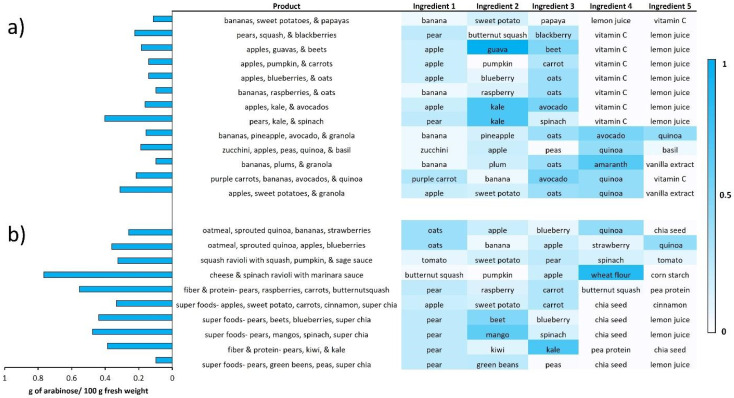
Heatmap of 23 commercial complementary foods from Happy Family brand. The bar graphs on the left represent the total amount of arabinose found in the complementary food product. The corresponding list of ingredients for each product on the right is provided as a heatmap of arabinose content (mg/mg dry wt.) for babies (**a**) and toddlers (**b**).

To investigate the carbohydrate content in processed foods or foods containing multiple ingredients such as commercial complementary foods, we compared the levels of arabinose in 23 products in a subset of a store name brand (Happy Family Brand foods). In processed foods, multiple ingredients were used to make the final product where the monosaccharide abundances of ingredients vary. As the ingredients mostly contained raw food ingredients, the arabinose concentration (by fresh weight) for that whole food was used to generate the heat map in Figure 6 for commercial complementary foods. For example, the raw ingredients for “Happy Tot Super Foods: pears, mangoes, spinach, super chia” included raw pears, mangoes, and spinach and were found to contain 0.27, 0.67, and 0.17 g of arabinose/100 g of fresh weight, respectively. Among this product, the arabinose content in mango (0.67 g/100 g fresh weight) was highest from all ingredients, while the total arabinose content of the complementary food product had lower amounts (0.47 g/100 g fresh weight). The rest of the complementary food for babies and toddlers had a varying range from 0.097 to 0.77 of arabinose g/100 g fresh weight. 

The first whole food ingredient in processed foods contributes greatly to the monosaccharide composition. For example, when bananas were the first ingredients in the “Happy Family” infant products, the total arabinose content was low (less than 0.2 g of arabinose/100 g fresh weight). On the other hand, when pears were the first ingredients, the total arabinose had greater than 0.2 g of arabinose/100 g fresh weight with exception of the “Super Foods: pears, green beans, peas, super chia” product. The cheese and spinach ravioli with marinara sauce meal in the “Happy Family” toddler product yielded the highest arabinose content, likely due to the minimal moisture content. 

## 4. Discussion

Current methods in dietary carbohydrate analysis are limited to quantifying sugars, starch, and fiber. Within the definition of fiber is an immense amount of structural complexity that can alter the gut microbiome and affect the health of the host. Dietary recommendations emphasize the importance of consuming fiber. However, the term “fiber” makes no distinction of the monosaccharide composition, nor the primary structure of the molecule. The reality is that food glycans are composed of a very large number of compounds, each with their unique structural variations and potentially specific activities both to the consuming host and their associated microbiome. Thus, the advice “eat more fiber,” is not meaningful as fiber from two different sources can have completely different monosaccharide compositions, glycosidic bond linkages, degrees of polymerization, and in turn, biological functions. The analytical methods used to measure carbohydrates must be updated to match the evolving throughput and coverage of genomic sequencing and metabolomic analyses. To address this need, we developed and employed a rapid-throughput, LC-MS based monosaccharide analysis to determine the total monosaccharide composition of 828 foods to create the Davis Food Glycopedia (DFG), which will inform future feeding studies in infants transitioning to complementary diets, toddlers and adults. The total monosaccharide composition and quantitation provides more useful information on dietary carbohydrates than traditional gravimetric methods especially in the context of the gut microbiome and infant nutrition. This comes with greatly increased sample throughput making the construction of large food glycan libraries possible. 

The DFG revealed the most abundant monosaccharide in the foods was primarily glucose from simple sugars such as sucrose and from starch polysaccharides. From an evolutionary and agricultural perspective, humans have historically used innovative strategies to seek and cultivate sugar- and starch-dense foods and parts of foods as a source of energy as evidenced by the expansion of salivary amylase genes in humans [26]. While these energy-rich foods were once a necessity for survival, increasingly sedentary lifestyles, and overconsumption of highly processed versions of these foods has contributed to a variety of metabolic disorders such as obesity, type 2 diabetes, and heart disease, particularly in Western populations [4]. The Glycopedia provides information not only on digestible glucose content (i.e., starch), but also on non-glucose content corresponding to various dietary fiber structures. This information can be used to inform dietary choices to alleviate these metabolic disorders by reducing starch and sugar consumption and increasing the consumption of specific fiber types to shape the gut microbiome in a targeted manner. Clustering analysis of the DFG revealed that the food group does not necessarily inform a food’s carbohydrate composition. For example, Fruits and Vegetables are two food groups that clustered together due to their similar monosaccharide compositions with higher average GalA and rhamnose, which reflect their pectin content. In contrast, Grain Products clustered away from Fruits and Vegetables due to their high glucose, xylose, and arabinose content, which reflected their starch, β-glucan, and arabinoxylan polysaccharide constituents. Together, these results suggest that diets meant to target the gut microbiome should be informed by the carbohydrate composition rather than the food group alone [27,28,29,30,31,32]. Indeed, carbohydrates represent a major carbon source for gut microbes. However, the intent is that the DFG will encourage similar efforts towards other macronutrients, such as lipids and proteins. This research also demonstrated monosaccharide compositions can vary within food groups with several implications for nutrition research. For example, it will be necessary for nutrition studies to resolve dietary data at the individual food level, rather than summarizing servings at the food group level, if the intent is to study food-microbiome structure relationships. Mixed meals will need to be resolved at the ingredient level. The database will eventually need to be expanded to incorporate the full variety of plant products consumed. The effects of ripeness and other biological variations will be addressed in future iterations of the DFG.

The broad epidemiologic importance of carbohydrates in the diet is well established. Their role in caloric transfer is critical to human health, however, this simple view belies their important intrinsic biological activities. Increased consumption of carbohydrates that resist digestion by the host, typically termed “dietary fiber,” has been associated with a reduced risk of obesity, type 2 diabetes, certain gastrointestinal disorders, and coronary heart disease [33,34]. Even monosaccharides, the smallest carbohydrate unit, have their own inherent activities [35]. More recently, the ability of carbohydrates to modulate the gut microbiome has become of considerable interest [10,36]. Furthermore, numerous lines of evidence demonstrate that specific oligosaccharides and polysaccharides have direct effects on human cells, even in the absence of microbes, influencing intestinal barrier function and inflammation in vitro [27,28,29,30,31,32]. Thus, the carbohydrate component of diet potentially has far-reaching effects that we can now begin to investigate in the context of whole foods, rather than in the context of isolated polysaccharides such as inulin [27,28,29,30,31,32]. While carbohydrates in food are undisputedly a necessary part of any healthy diet, the relative amounts, types of carbohydrates, and whether some foods can be called carbohydrates at all are the subject of considerable and even broad disagreements. Such conflicts regarding carbohydrates stem from our general ignorance of their chemical structures. These limitations can directly affect feeding trials, particularly those related to food-microbiome interactions. Once we understand food structures, then variations stemming from, for example, sampling, individual subjects, populations, and even data analysis can be more readily addressed. The response of the microbiome to specific dietary intervention can be more readily deduced. The DFG will be an important resource in addressing these and other issues. The intent of this database is to improve and guide study design in clinical feeding trials.

Even within the current DFG, there lies an enormous amount of structural diversity, which is not captured as the polysaccharide and glycosidic linkages were not obtained. Additionally, the methods here did not employ steps to separate free sugars and oligosaccharides from polysaccharides. Thus, for example, free fructose and glucose were not differentiated from inulin or starch, respectively. Future iterations of the DFG will use rapid-throughput analytical workflows that will separate free saccharides from polysaccharides and provide linkage and polysaccharide compositions, while free saccharides will be quantitated separately. These amendments will further provide a more comprehensive and higher level resolution picture of food carbohydrates.

## Figures and Tables

**Table 1:** The absolute monosaccharide composition and amounts in an example dinner meal.

				Monosaccharide (g)														
Food	Serving Amount	Amount (Grams)	Moisture (%)	Glc	Gal	Fruc	Xyl	Ara	Fuc	Rhm	GlcA	Gal A	GlcNAc	GalNAc	Man	All	Rib	Total
grilled chicken breast	4 oz	113	62.0	0.14	0.01	0.03	0.00	0.00	0.00	0.00	0.00	0.00	0.00	0.00	0.03	0.00	0.17	0.40
steamed broccoli	0.5 cups	38	89.6	0.36	0.13	0.00	0.06	0.14	0.01	0.02	0.00	0.12	0.00	0.00	0.02	0.00	0.02	0.90
steamed carrots	0.33 cups	50	87.1	1.66	0.29	0.17	0.03	0.16	0.02	0.02	0.00	0.26	0.00	0.00	0.04	0.00	0.00	2.70
steamed butternut squash	0.33 cups	67	89.6	2.29	0.19	0.42	0.03	0.03	0.01	0.01	0.00	0.20	0.00	0.00	0.02	0.00	0.00	3.20
cooked pasta	0.75 cups	150	54.7	57.60	0.51	0.45	2.10	1.51	0.00	0.02	0.01	0.03	0.00	0.00	0.15	0.00	0.03	62.40
olive oil	1 Tbsp	14	0.8	0.02	0.00	0.01	0.00	0.00	0.00	0.00	0.00	0.00	0.00	0.00	0.00	0.00	0.00	0.00
navel orange	1 medium orange	165	87.3	6.37	0.37	2.64	0.12	0.24	0.04	0.06	0.00	0.60	0.00	0.00	0.00	0.00	0.00	10.40
whole milk	1 cup of milk	245	89.0	4.27	4.74	0.00	0.00	0.01	0.00	0.00	0.00	0.00	0.00	0.00	0.03	0.00	0.01	9.10
total	N/A	842	N/A	72.70	6.25	3.72	2.35	2.09	0.09	0.13	0.01	1.21	0.00	0.00	0.30	0.00	0.25	89.09

## Data Availability

Glycopedia monosaccharide data, sample metadata, and scripts used for analysis and figures are available at https://github.com/quarksome/Food-Glycopedia (accessed on 13 April 2022). An interactive dashboard to visualize the monosaccharide data is hosted at https://dash-app-fooden.herokuapp.com (accessed on 13 April 2022).

## Supplementary Materials

The following supporting information can be downloaded at: https://www.mdpi.com/article/10.3390/nu14081639/s1. The Supplementary Materials include Table S1 of all food present in the Davis Food Glycopedia with corresponding, UIC number, monosaccharide value, % moisture, and simple food descriptions. Supplementary Figure S1 depicts dMRM chromatograms of a monosaccharide pool and selected food samples. Supplementary Figure S2 is a control chart showing the measured concentrations of the QC samples for each batch. Supplementary Figure S3 describes the number of foods assigned to each of nine total food groups. Supplementary Figure S4 shows difference in total non-glucose monosaccharides between whole grain and “white” grain products. Supplementary Figure S5 provides the monosaccharide compositions of nine spinach samples included in the DFG. Supplementary Figure S6 illustrates an enrichment factor heatmap for each food group and cluster. Supplementary Figure S7 depicts the average monosaccharide compositions of the generated sub-clusters A-H of Cluster 1. Supplementary Figure S8 illustrates the mass percentages of food groups used to generate an example meal based on recommendations in the USDA Dietary Guidelines for Americans 2020–2025. Supplementary Figure S9 shows pear cultivars tended to contain more arabinose content than apple cultivars on average.
