# Supplementary material for: The Development of the Davis Food Glycopedia—A Glycan Encyclopedia of Food
Source: Nutrients. 2022 Apr 14;14(8):1639. doi: 10.3390/nu14081639 (PMC9032246; doi:10.3390/nu14081639)
Supplement: Supplementary file 1 [file nutrients-14-01639-s001.zip › nutrients-1652477-supplementary.pdf]

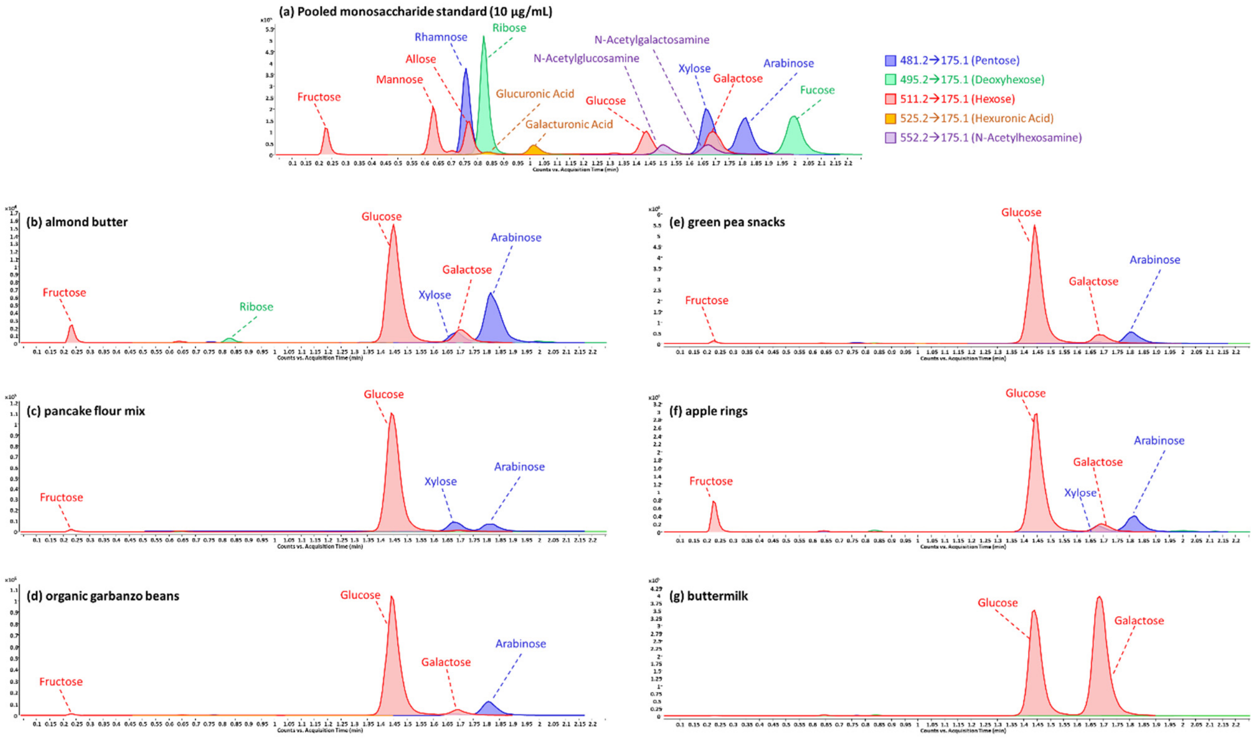

Supplementary Figure S1. Dynamic multiple reaction monitoring (dMRM) chromatograms depicting the monosaccharide profiles of (a) a pooled 10 µg/mL monosaccharide standard, (b) almond butter, (c) pancake flour mix, (d) organic garbanzo beans, (e) green pea snacks, (f) apple rings, and (g) buttermilk.

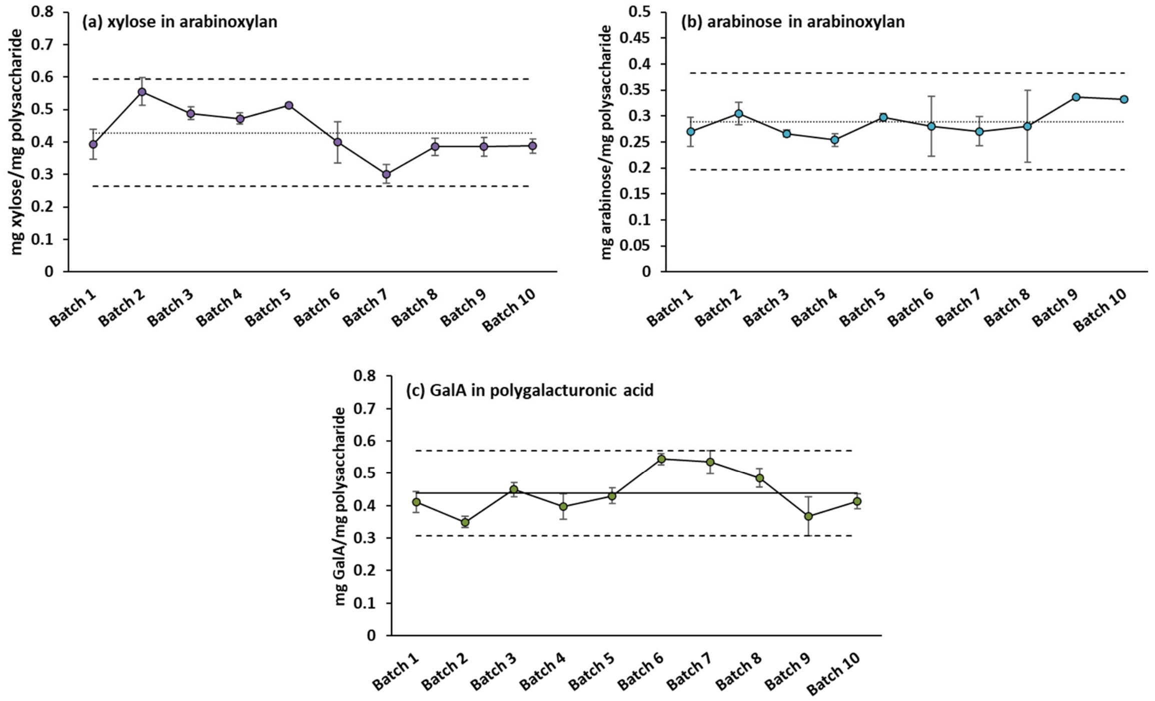

Supplementary Figure S2. Control diagrams depicting the polysaccharide standard quality controls (QCs) analyzed with each of ten batches of samples. (a) Xylose measured in the arabinoxylan control. (b) Arabinose measured in the arabinoxylan control. (c) GalA measured in

the polygalacturonic acid control. The dashed lines indicate +/- two times the standard deviation. The dotted middle lines indicate the average.

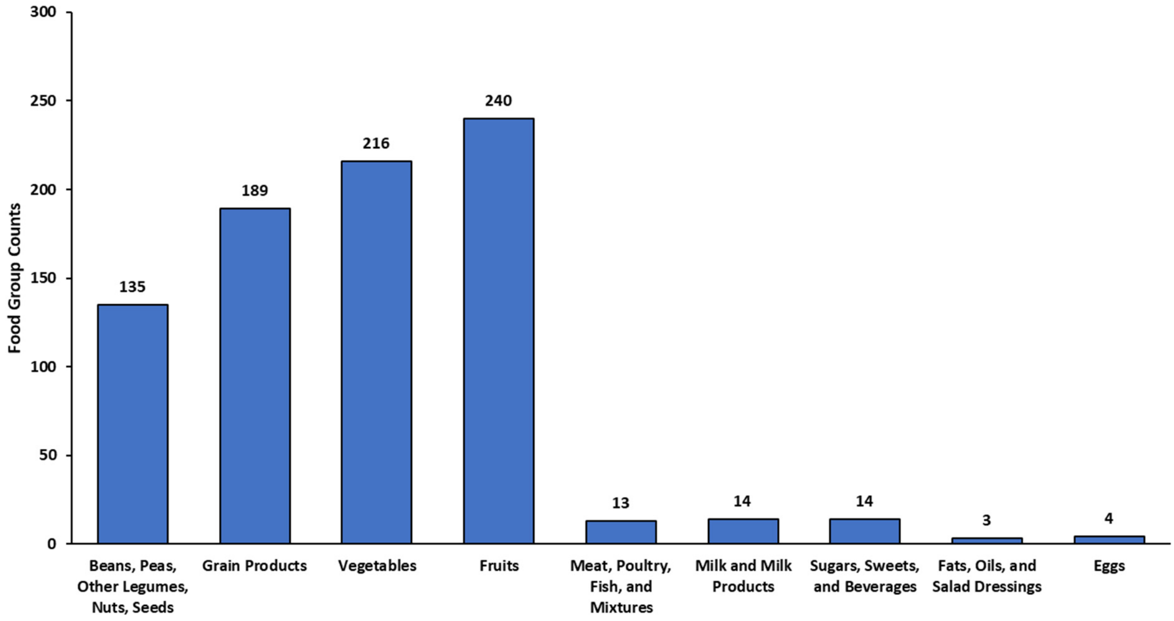

Supplementary Figure S3. Number of foods assigned to each of nine total food groups. Those groups containing plant-based foods contributed the largest number while animal-based and low carbohydrate groups contributed the least.

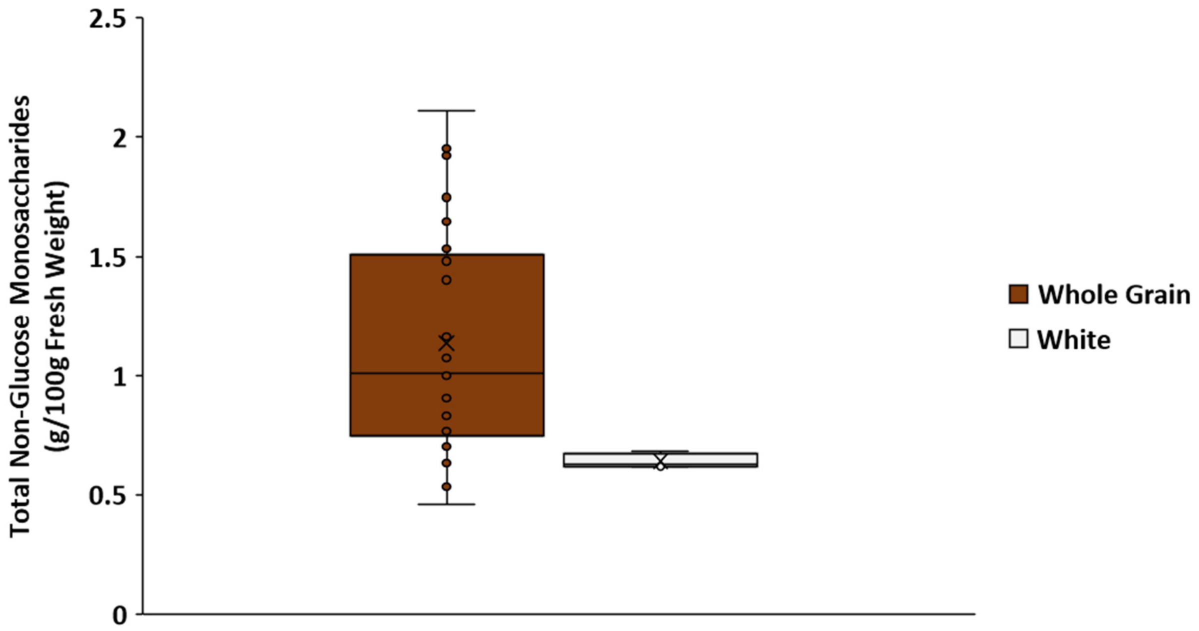

Supplementary Figure S4. Difference in total non-glucose monosaccharides between whole grain and "white" grain products. Although not statistically significant, whole grain bread tended to contain more non-glucose residues (mostly from xylose, arabinose, and galactose).

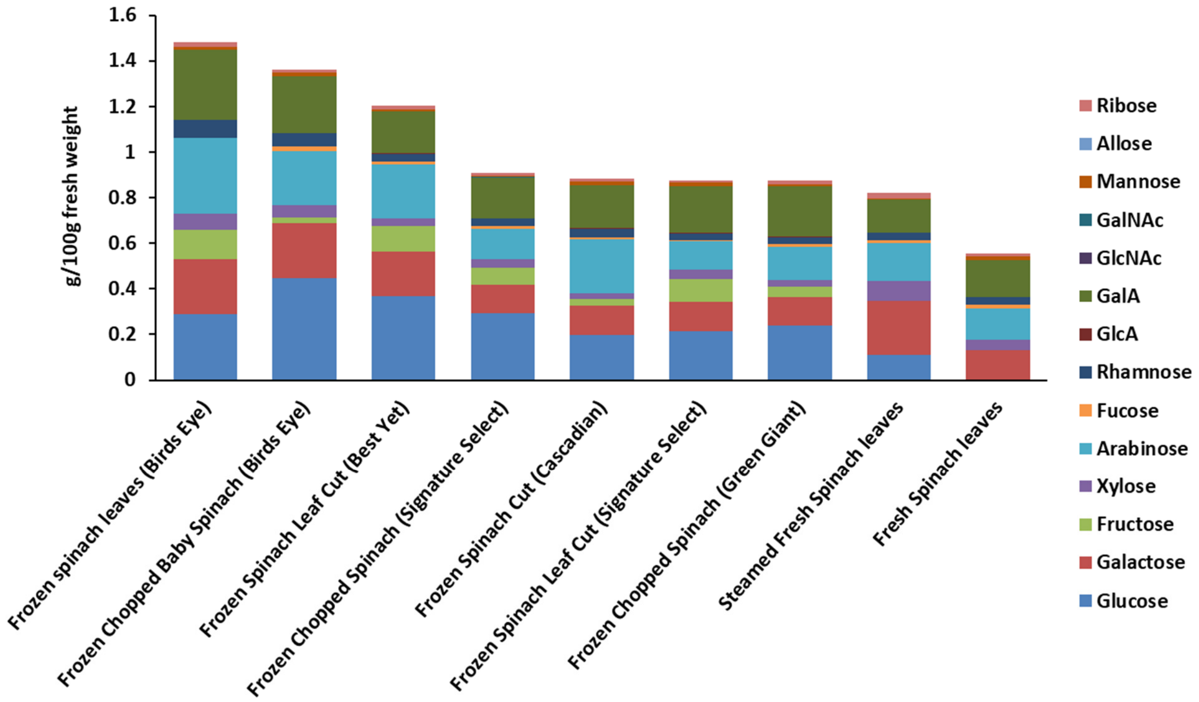

Supplementary Figure S5. Monosaccharide compositions of nine spinach samples included in the DFG including frozen varieties from several brands as well as fresh and steamed fresh preparations.

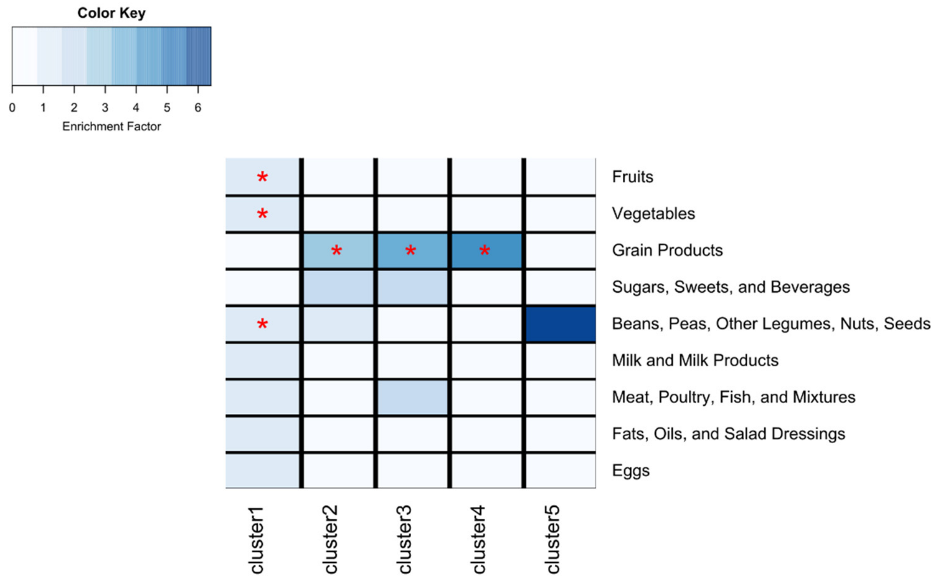

Supplementary Figure S6. Enrichment factor heatmap for each food group and cluster. Red asterisk indicates a significance of $p < 0.05$ using FDR adjusted p-values derived from a hypergeometric (over-representation) test.

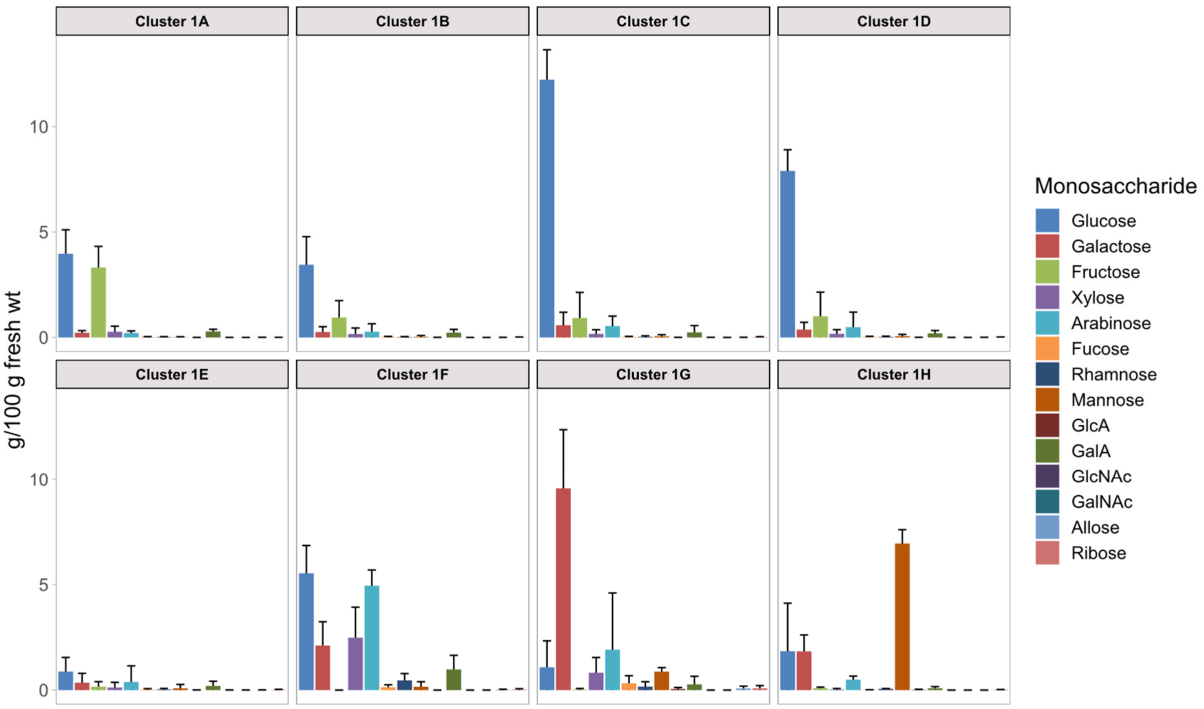

Supplementary Figure S7. Average monosaccharide compositions of the sub-clusters A-H of Cluster 1. Error bars represent the standard deviation.

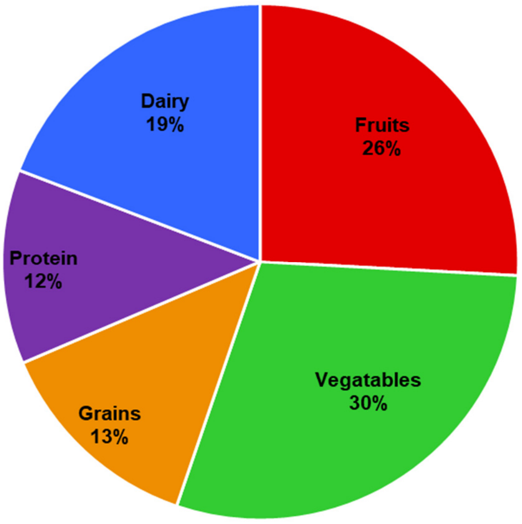

Supplementary Figure S8. Mass percentages of food groups used to generate an example meal based on recommendations in the USDA Dietary Guidelines for Americans 2020-2025.

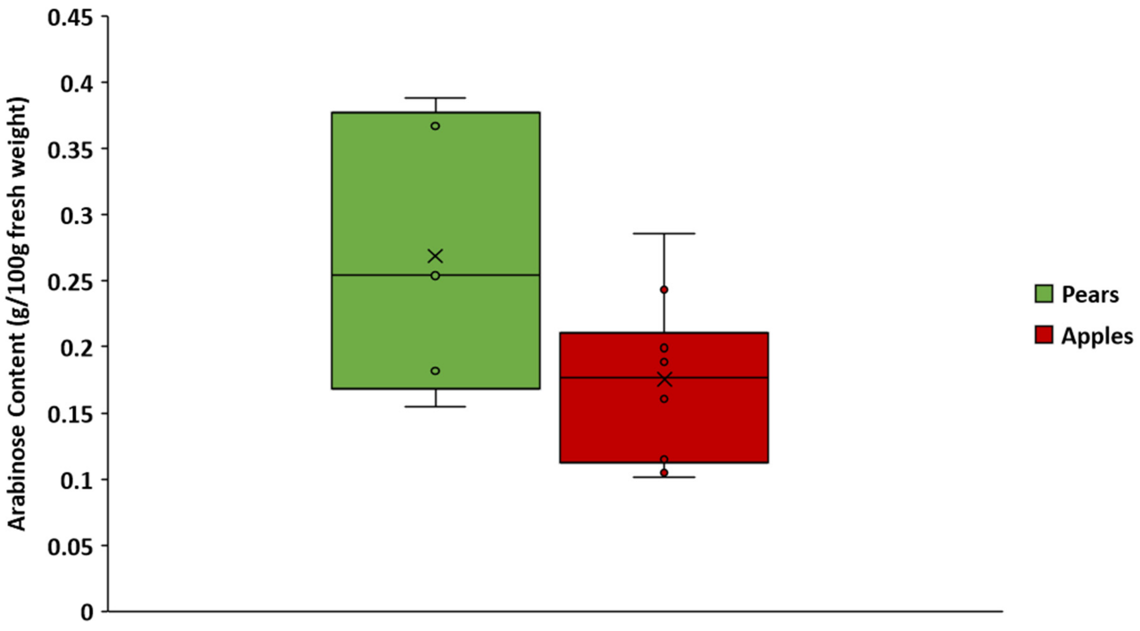

Supplementary Figure S9. Although not statistically significant, pear cultivars tended to contain more arabinose content than apple cultivars on average.

Table S1. Monosaccharide compositions, food groups, and cluster numbers of all 828 foods surveyed. Units are g/100 fresh weight. N.D = "Not Detected".

Sample Number	Sample Name	Food Group	Moisture Content (%)	Cluster Number	Glucose	Galactose	Fructose	Xylose	Arabinose	Fucose	Rhamnose	GlcA	GalA	GlcNAc	GalNAc	Mannose	Allose	Ribose	Total
1	Whole envy apple w/o seed	Fruits	80.79	cluster1	4.55	0.17	2.42	0.12	0.20	0.04	0.01	N.D	0.24	N.D	N.D	N.D	N.D	0.01	7.75
2	Envy apple flesh only	Fruits	80.79	cluster1	3.61	0.18	2.05	0.14	0.14	0.05	0.01	N.D	0.18	N.D	N.D	N.D	N.D	N.D	6.37
3	Whole jazz apple w/o seed	Fruits	85.29	cluster1	2.53	0.14	1.81	0.17	0.10	0.05	0.01	N.D	0.23	N.D	N.D	N.D	N.D	N.D	5.04
4	Jazz apple flesh only	Fruits	86.70	cluster1	2.40	0.09	1.73	0.14	0.06	0.03	0.01	N.D	0.18	N.D	N.D	N.D	N.D	N.D	4.63
5	Whole Honeycrips apple w/o seed	Fruits	85.73	cluster1	2.43	0.10	1.96	0.09	0.11	0.03	0.01	N.D	0.15	N.D	N.D	0.00	N.D	N.D	4.90
6	Honeycrips apple flesh only	Fruits	86.86	cluster1	2.03	0.08	2.06	0.09	0.08	0.03	0.01	N.D	0.12	N.D	N.D	N.D	N.D	N.D	4.51
7	Whole Pink Lady apple w/o seed	Fruits	84.14	cluster1	2.73	0.17	2.11	0.14	0.24	0.05	0.02	N.D	0.30	N.D	N.D	0.03	N.D	N.D	5.79
8	Pink Lady apple flesh only	Fruits	84.52	cluster1	2.87	0.18	2.37	0.15	0.24	0.06	0.02	N.D	0.24	N.D	N.D	N.D	N.D	N.D	6.13
9	Whole Fuji apple w/o seed	Fruits	83.81	cluster1	2.19	0.04	2.08	0.11	0.11	0.03	0.01	N.D	0.15	N.D	N.D	N.D	N.D	N.D	4.71
10	Fuji apple flesh only	Fruits	83.71	cluster1	2.93	0.15	2.98	0.19	0.13	0.04	0.02	N.D	0.17	N.D	N.D	N.D	N.D	N.D	6.60
11	Whole Gala apple w/o seed	Fruits	85.54	cluster1	2.55	0.17	2.33	0.28	0.17	0.06	0.02	N.D	0.24	N.D	N.D	N.D	N.D	N.D	5.83
12	Gala apple flesh only	Fruits	85.44	cluster1	3.16	0.17	2.24	0.28	0.13	0.06	0.02	N.D	0.21	N.D	N.D	N.D	N.D	N.D	6.26
13	Whole Golden Del apple w/o seed	Fruits	84.70	cluster1	2.83	0.16	2.23	0.12	0.19	0.03	0.02	N.D	0.27	N.D	N.D	N.D	N.D	N.D	5.85

14	Golden Del apple flesh only	Fruits	85.07	cluster1	2.44	0.16	1.38	0.12	0.19	0.07	0.02	N.D	0.21	N.D	N.D	N.D	N.D	N.D	4.59
15	Whole Red Delicious apple w/o seed	Fruits	81.18	cluster1	3.38	0.29	2.08	0.18	0.29	0.03	0.02	N.D	0.26	N.D	N.D	0.01	N.D	N.D	6.54
16	Red Delicious apple flesh only	Fruits	81.56	cluster1	4.42	0.27	2.89	0.19	0.27	0.04	0.02	N.D	0.25	N.D	N.D	0.02	N.D	N.D	8.36
17	Whole Granny Smith apple w/o seed	Fruits	85.81	cluster1	3.13	0.22	1.58	0.23	0.19	0.05	0.02	N.D	0.31	N.D	N.D	0.04	N.D	N.D	5.75
18	Granny Smith apple flesh only	Fruits	85.88	cluster1	3.20	0.30	2.45	0.29	0.21	0.06	0.02	N.D	0.35	N.D	N.D	0.03	N.D	N.D	6.92
19	Whole Pazazz apple w/o seed	Fruits	85.47	cluster1	2.97	0.20	3.28	0.18	0.16	0.04	0.02	N.D	0.20	N.D	N.D	0.02	N.D	N.D	7.07
20	Pazazz apple flesh only	Fruits	85.74	cluster1	1.96	0.16	1.16	0.13	0.13	0.05	0.01	N.D	0.16	N.D	N.D	N.D	N.D	N.D	3.76
21	Whole Red D'anjou pear w/o seed	Fruits	83.62	cluster1	1.83	0.18	1.64	2.07	0.39	0.02	0.03	N.D	0.28	N.D	N.D	0.01	N.D	N.D	6.45
22	Whole Bartlett pear w/o seed	Fruits	80.89	cluster1	1.77	0.14	1.01	0.31	0.37	N.D	0.02	N.D	0.27	N.D	N.D	0.06	N.D	N.D	3.94
23	Whole D'anjou pear w/o seed	Fruits	84.39	cluster1	2.09	0.10	1.80	0.25	0.18	0.02	0.02	N.D	0.26	N.D	N.D	0.00	N.D	N.D	4.72
24	Whole Beurre Bosc pear w/o seed	Fruits	85.48	cluster1	1.81	0.15	4.74	1.42	0.15	0.03	0.03	N.D	0.27	N.D	N.D	N.D	0.01	N.D	8.61
25	Whole Shingo pear w/o seed	Fruits	82.45	cluster1	3.78	0.10	2.53	0.16	0.25	0.02	0.02	N.D	0.25	N.D	N.D	0.00	N.D	N.D	7.10
26	Whole interspecific aprium	Fruits	87.93	cluster1	3.24	0.11	0.99	0.08	0.16	0.02	0.02	N.D	0.22	N.D	N.D	0.01	N.D	N.D	4.86
27	Whole apricot	Fruits	86.15	cluster1	3.13	0.17	0.78	0.14	0.21	0.03	0.02	N.D	0.29	N.D	N.D	0.01	N.D	N.D	4.79
28	Whole black plum	Fruits	84.48	cluster1	3.61	0.37	1.45	0.09	0.19	0.02	0.02	N.D	0.31	N.D	N.D	N.D	N.D	0.01	6.07

29	Whole red plum	Fruits	82.91	cluster1	4.78	0.48	1.41	0.12	0.19	0.03	0.03	N.D	0.27	N.D	N.D	0.03	N.D	N.D	7.33
30	Whole flavor gator pluot	Fruits	86.21	cluster1	3.86	0.34	1.11	0.09	0.16	0.02	0.02	N.D	0.18	N.D	N.D	0.01	N.D	N.D	5.79
31	Whole black plum (HGO farms)	Fruits	84.11	cluster1	3.53	0.38	1.20	0.12	0.21	0.03	0.02	N.D	0.35	N.D	N.D	0.03	N.D	N.D	5.87
32	Whole Plumogranate pluot	Fruits	87.34	cluster1	3.46	0.25	0.70	0.11	0.15	0.02	0.02	N.D	0.22	N.D	N.D	0.01	N.D	N.D	4.94
33	Whole white nectarine (HGO Farms)	Fruits	85.64	cluster1	4.50	0.22	1.75	0.15	0.29	0.04	0.02	N.D	0.30	N.D	N.D	0.01	N.D	N.D	7.28
34	Whole white saturn peach	Fruits	84.44	cluster1	5.83	0.18	1.93	0.12	0.27	0.03	0.02	N.D	0.33	N.D	N.D	0.01	N.D	N.D	8.71
35	Whole white peach	Fruits	86.14	cluster1	4.91	0.15	1.57	0.07	0.26	0.02	0.02	N.D	0.32	N.D	N.D	0.02	N.D	N.D	7.34
36	Whole yellow nectarine	Fruits	83.41	cluster1	4.69	0.17	1.32	0.08	0.27	0.02	0.02	N.D	0.33	N.D	N.D	0.01	N.D	0.01	6.91
37	Whole yellow peach (Prima Farms)	Fruits	90.25	cluster1	2.98	0.14	1.28	0.11	0.14	0.02	0.01	N.D	0.21	N.D	N.D	0.01	N.D	0.01	4.91
38	Whole yellow nectarine (overipe)	Fruits	87.35	cluster1	4.47	0.16	1.95	0.10	0.20	0.02	0.02	N.D	0.34	N.D	N.D	0.00	N.D	0.01	7.27
39	Yellow nectarine flesh only	Fruits	88.96	cluster1	3.89	0.11	1.69	0.09	0.14	0.02	0.01	N.D	0.25	N.D	N.D	0.01	N.D	0.00	6.20
40	Whole yellow peach (HGO Farms)	Fruits	83.89	cluster1	5.65	0.21	2.26	0.10	0.24	0.04	0.02	N.D	0.29	N.D	N.D	0.01	N.D	0.01	8.81
41	Whole white nectarine (Summeripe)	Fruits	86.29	cluster1	5.22	0.14	2.32	0.06	0.20	0.02	0.01	N.D	0.26	N.D	N.D	0.01	N.D	0.00	8.24
42	Pureed whole golden dell apple w/o seed	Fruits	85.27	cluster1	2.32	0.18	1.88	0.13	0.22	0.06	0.02	N.D	0.27	N.D	N.D	0.03	N.D	N.D	5.10

43	Pureed whole bartlett pear w/o seed	Fruits	80.75	cluster1	2.25	0.19	2.41	0.59	0.49	0.04	0.03	N.D	0.29	N.D	N.D	0.05	N.D	N.D	6.32
44	Pureed whole honeycrisp apple w/o seed	Fruits	88.13	cluster1	2.74	0.17	2.05	0.12	0.20	0.05	0.02	N.D	0.27	N.D	N.D	0.03	N.D	N.D	5.64
45	Red mango	Fruits	88.50	cluster1	1.69	0.14	0.64	0.13	0.22	0.05	0.01	N.D	0.23	N.D	N.D	0.00	N.D	N.D	3.12
46	Hass (large) avocado	Fruits	56.52	cluster1	0.92	0.18	1.35	0.17	0.51	N.D	0.06	N.D	1.23	N.D	N.D	0.01	N.D	N.D	4.43
47	Hass (small) avocado	Fruits	73.00	cluster1	0.39	0.17	0.60	0.16	0.43	N.D	0.05	N.D	0.92	N.D	N.D	0.03	N.D	N.D	2.75
48	Whole Green house (large) tomato	Vegetables	93.96	cluster1	1.60	0.06	0.86	0.02	0.03	0.00	0.01	N.D	0.22	N.D	N.D	0.07	N.D	N.D	2.88
49	Whole Glorys tomato	Vegetables	91.48	cluster1	1.84	0.14	0.74	0.08	0.07	0.00	0.01	N.D	0.26	N.D	N.D	0.01	N.D	N.D	3.17
50	Whole Twilight tomato	Vegetables	89.19	cluster1	2.49	0.16	0.71	0.11	0.10	0.01	0.01	N.D	0.29	N.D	N.D	0.04	N.D	N.D	3.93
51	Whole Comets tomato	Vegetables	91.15	cluster1	2.06	0.21	0.69	0.10	0.11	0.01	0.01	N.D	0.22	N.D	N.D	0.18	N.D	N.D	3.60
52	Whole Cherubs tomato	Vegetables	89.46	cluster1	2.72	0.28	1.00	0.16	0.16	0.01	0.02	N.D	0.30	N.D	N.D	0.14	N.D	N.D	4.78
53	Whole blueberry	Fruits	84.89	cluster1	4.75	0.21	0.71	0.25	0.09	0.04	0.01	N.D	0.20	N.D	N.D	N.D	N.D	N.D	6.27
54	Whole raspberry	Fruits	82.65	cluster1	5.31	0.22	4.54	0.17	0.14	0.02	0.02	N.D	0.22	N.D	N.D	0.03	0.01	N.D	10.70
55	Whole blackberry	Fruits	84.74	cluster1	3.98	0.25	1.48	0.15	0.33	0.04	0.03	N.D	0.43	N.D	N.D	0.05	N.D	N.D	6.74
56	Whole strawberry	Fruits	87.50	cluster1	3.15	0.14	0.43	0.10	0.10	0.06	0.02	N.D	0.24	N.D	N.D	0.00	N.D	0.01	4.25
57	Whole goldenberry	Fruits	79.02	cluster1	5.06	0.16	1.76	0.08	0.14	0.01	0.02	N.D	0.23	N.D	N.D	0.02	N.D	N.D	7.48
58	Whole sweet cherry	Fruits	78.89	cluster1	6.71	0.16	4.24	0.07	0.20	0.01	0.08	N.D	0.19	N.D	N.D	N.D	0.01	N.D	11.66
59	Whole guava	Fruits	77.81	cluster1	3.02	0.51	1.43	3.05	1.07	0.07	0.07	N.D	0.58	N.D	N.D	0.05	N.D	N.D	9.85
60	Navel orange	Fruits	87.27	cluster1	3.86	0.22	1.60	0.07	0.15	0.02	0.04	N.D	0.36	N.D	N.D	N.D	N.D	N.D	6.32

61	Valencia orange	Fruits	85.61	cluster1	3.78	0.27	1.36	0.06	0.30	0.02	0.06	N.D	0.74	N.D	N.D	0.03	N.D	N.D	6.63
62	Deep red grapefruit	Fruits	87.92	cluster1	3.71	0.18	0.89	0.08	0.10	0.02	0.04	N.D	0.37	N.D	N.D	0.01	N.D	N.D	5.41
63	Kiwi	Fruits	85.64	cluster1	3.40	0.59	1.68	0.29	0.12	0.03	0.03	N.D	0.46	N.D	N.D	0.05	N.D	N.D	6.63
64	Cantaloupe melon	Fruits	88.78	cluster1	1.66	0.12	0.69	0.03	0.06	0.01	0.01	N.D	0.19	N.D	N.D	0.01	N.D	N.D	2.77
65	Honeydew melon	Fruits	92.11	cluster1	2.47	0.10	0.96	0.05	0.03	0.01	0.01	N.D	0.12	N.D	N.D	0.00	N.D	N.D	3.74
66	Seedless watermelon	Fruits	91.25	cluster1	2.50	0.09	2.70	0.05	0.03	0.01	0.00	N.D	0.09	N.D	N.D	N.D	0.00	N.D	5.47
67	Gold (golden) pineapple	Fruits	81.35	cluster1	6.31	0.18	0.99	0.21	0.17	0.05	0.00	N.D	0.03	N.D	N.D	0.00	N.D	N.D	7.94
68	Tai nung papaya	Fruits	86.98	cluster1	4.67	0.15	1.26	0.13	0.03	0.03	0.02	N.D	0.56	N.D	N.D	0.03	N.D	N.D	6.90
69	Mexican maradol papaya	Fruits	86.47	cluster1	4.23	0.12	1.37	0.10	0.04	0.02	0.02	N.D	0.50	N.D	N.D	N.D	N.D	N.D	6.38
70	Red seedless grape	Fruits	83.99	cluster1	5.68	0.17	4.11	0.10	0.07	0.02	0.01	N.D	0.17	N.D	N.D	0.00	0.01	N.D	10.34
71	Pureed red seedless grape	Fruits	86.57	cluster1	5.02	0.17	1.45	0.09	0.09	0.02	0.02	N.D	0.24	N.D	N.D	0.03	N.D	N.D	7.12
72	Pureed green seedless grape	Fruits	81.37	cluster1	5.96	0.14	2.19	0.08	0.11	0.02	0.02	N.D	0.20	N.D	N.D	0.05	N.D	N.D	8.77
73	Jicama	Vegetables	91.17	cluster1	4.64	0.09	0.57	0.02	0.07	0.01	0.01	N.D	0.19	N.D	N.D	N.D	N.D	N.D	5.60
74	Peeled, steamed beetroot	Vegetables	85.43	cluster1	4.04	0.25	0.38	0.03	0.45	0.05	0.03	N.D	0.19	N.D	N.D	0.01	N.D	0.00	5.45
75	Bunch beetroot flesh	Vegetables	85.40	cluster1	3.95	0.20	0.37	0.02	0.50	0.05	0.02	N.D	0.11	N.D	N.D	N.D	N.D	0.01	5.22
76	Bunch Steamed beetroot flesh	Vegetables	84.91	cluster1	4.05	0.23	0.38	0.05	0.55	0.04	0.03	N.D	0.16	N.D	N.D	0.01	N.D	0.01	5.50
77	SteamedLoose beetroot flesh	Vegetables	87.27	cluster1	2.96	0.24	0.27	0.11	0.53	0.03	0.04	N.D	0.22	N.D	N.D	0.03	N.D	0.00	4.43

78	Steamed Kale leaves (Organics)	Vegetables	87.72	cluster1	0.91	0.52	N.D	0.41	0.33	0.07	0.07	N.D	0.63	N.D	N.D	0.18	N.D	0.02	3.15
79	Steamed Kale leaves (Ratto Bros)	Vegetables	86.61	cluster1	1.60	0.81	0.29	0.73	0.76	0.12	0.10	N.D	1.03	N.D	N.D	0.24	N.D	N.D	5.66
80	Steamed Lacinato Kale leaves and stalk (Ratto Bros)	Vegetables	84.24	cluster1	0.88	0.51	N.D	0.18	0.52	0.05	0.10	N.D	0.74	N.D	N.D	0.13	N.D	0.03	3.14
81	Steamed Lacinato Kale leaves and stalk (Cal-Organics)	Vegetables	83.46	cluster1	1.43	0.90	N.D	0.43	0.64	0.08	0.09	N.D	0.76	N.D	N.D	0.26	N.D	0.04	4.62
82	Steamed Red chard leaves and stalk (Cal-Organic)	Vegetables	88.71	cluster1	0.74	0.26	N.D	0.12	0.65	0.02	0.09	N.D	0.19	N.D	N.D	0.05	N.D	0.01	2.14
83	Steamed Rainbow chard leaves and stalk (Cal-Organic)	Vegetables	91.06	cluster1	1.14	0.30	N.D	0.12	0.39	0.02	0.06	N.D	0.16	N.D	N.D	0.05	N.D	0.01	2.24
84	Steamed Green chard leaves and stalk (Cal-Organic)	Vegetables	86.87	cluster1	0.98	0.47	N.D	0.36	0.85	0.04	0.09	N.D	0.25	N.D	N.D	0.02	N.D	0.02	3.08
85	Steamed Red Chard leaves and stalk (Ratto Bros)	Vegetables	86.10	cluster1	1.17	0.35	N.D	0.24	0.79	0.02	0.07	N.D	0.21	N.D	N.D	0.06	N.D	0.03	2.92
86	Steamed Red kale leaves and stalk	Vegetables	88.26	cluster1	0.34	0.42	N.D	0.33	0.32	0.06	0.06	N.D	0.65	N.D	N.D	0.08	N.D	0.04	2.30

87	Steamed Collard greens leaves and stalk	Vegetables	87.70	cluster1	0.45	0.42	0.04	0.26	0.36	0.04	0.05	N.D	0.51	N.D	N.D	0.05	N.D	0.03	2.20
88	Steamed Collard greens leaves	Vegetables	89.48	cluster1	0.35	0.38	0.10	0.27	0.29	0.04	0.05	N.D	0.43	N.D	N.D	0.05	N.D	0.02	1.99
89	Steamed Curely mustard greens	Vegetables	89.29	cluster1	0.58	0.39	0.10	0.39	0.32	0.05	0.06	N.D	0.51	N.D	N.D	0.05	N.D	0.03	2.47
90	Steamed Spinach leaves	Vegetables	92.30	cluster1	0.11	0.24	N.D	0.09	0.17	0.01	0.03	N.D	0.14	N.D	N.D	0.00	N.D	0.03	0.82
91	Steamed Baby Bok Choy leaves and stalk	Vegetables	94.47	cluster1	0.55	0.11	0.11	0.11	0.06	0.02	0.03	N.D	0.28	N.D	N.D	0.02	N.D	0.01	1.30
92	Steamed Iceberg lettuce	Vegetables	94.96	cluster1	0.60	0.11	N.D	0.13	0.04	0.02	0.02	N.D	0.17	N.D	N.D	0.02	N.D	0.01	1.12
93	Spinach leaves	Vegetables	91.41	cluster1	0.00	0.13	N.D	0.05	0.14	0.02	0.04	N.D	0.16	N.D	N.D	0.02	N.D	0.01	0.55
94	Steamed Whole brussel sprouts	Vegetables	80.70	cluster1	4.49	0.63	0.36	0.34	0.85	0.09	0.07	N.D	0.68	N.D	N.D	0.11	N.D	0.02	7.64
95	Steamed Crown broccoli florets and stalk	Vegetables	89.62	cluster1	0.94	0.33	N.D	0.16	0.37	0.04	0.04	N.D	0.31	N.D	N.D	0.06	N.D	0.04	2.30
96	Steamed Cauliflower florets and stalk	Vegetables	91.57	cluster1	0.91	0.20	N.D	0.09	0.26	0.03	0.03	N.D	0.19	N.D	N.D	0.03	N.D	0.02	1.76
97	Steamed Celery stalk	Vegetables	95.17	cluster1	0.66	0.15	N.D	0.10	0.11	0.02	0.02	N.D	0.18	N.D	N.D	0.03	N.D	0.00	1.27
98	Steamed whole snow peas	Beans, Peas, Legumes,	85.59	cluster1	5.45	0.44	0.42	0.23	0.25	0.03	0.03	N.D	0.32	N.D	N.D	0.00	N.D	0.02	7.20

		Nuts, Seeds																	
99	Whole snow peas	Beans, Peas, Legumes, Nuts, Seeds	84.92	cluster1	5.61	0.40	0.29	0.06	0.24	0.01	0.03	N.D	0.32	N.D	N.D	N.D	N.D	0.02	6.98
100	Steamed Green beans	Vegetables	88.54	cluster1	2.56	0.49	N.D	0.31	0.27	0.05	0.04	N.D	0.49	N.D	N.D	0.20	N.D	0.03	4.44
101	Steamed French green beans	Vegetables	92.61	cluster1	0.76	0.38	N.D	0.17	0.16	0.05	0.03	N.D	0.33	N.D	N.D	0.07	N.D	0.02	1.96
102	Steamed Sugar snap pea	Beans, Peas, Legumes, Nuts, Seeds	85.60	cluster1	3.70	0.46	N.D	0.12	0.26	0.03	0.04	N.D	0.36	N.D	N.D	0.02	N.D	0.03	5.01
103	Steamed peeled baby carrot	Vegetables	89.49	cluster1	2.58	0.38	0.25	0.05	0.23	0.04	0.04	N.D	0.43	N.D	N.D	0.05	N.D	0.00	4.06
104	Peeled baby carrot	Vegetables	89.75	cluster1	2.21	0.30	0.03	0.08	0.24	0.02	0.04	N.D	0.36	N.D	N.D	0.04	N.D	0.01	3.34
105	Steamed peeled white rainbow baby carrots	Vegetables	90.11	cluster1	2.52	0.36	0.33	0.08	0.32	0.02	0.04	N.D	0.46	N.D	N.D	0.04	N.D	0.01	4.17
106	Steamed peeled yellow rainbow baby carrots	Vegetables	88.86	cluster1	3.62	0.43	0.26	0.07	0.28	0.05	0.04	N.D	0.52	N.D	N.D	0.04	N.D	0.01	5.33
107	Steamed peeled purple rainbow baby carrots	Vegetables	88.38	cluster1	3.79	0.44	0.80	0.09	0.26	0.02	0.04	N.D	0.44	N.D	N.D	0.05	N.D	0.00	5.93
108	Steamed whole carrots	Vegetables	87.11	cluster1	3.32	0.59	0.34	0.05	0.32	0.04	0.04	N.D	0.53	N.D	N.D	0.07	N.D	0.01	5.30

109	Steamed carrot flesh only	Vegetables	87.53	cluster1	1.56	0.52	0.70	0.08	0.31	0.02	0.04	N.D	0.44	N.D	N.D	0.02	N.D	0.01	3.70
110	Steamed whole green bell pepper w/o seed	Vegetables	93.57	cluster1	1.34	0.18	0.07	0.06	0.06	0.03	0.02	N.D	0.25	N.D	N.D	0.01	N.D	0.00	2.01
111	Steamed whole red bell pepper w/o seed	Vegetables	92.42	cluster1	1.65	0.04	0.05	0.01	0.02	0.03	0.02	N.D	0.24	N.D	N.D	N.D	N.D	0.00	2.07
112	Steamed whole yellow bell pepper w/o seed	Vegetables	91.15	cluster1	1.97	0.05	0.15	0.02	0.03	0.03	0.02	N.D	0.22	N.D	N.D	N.D	N.D	0.00	2.49
113	Steamed whole orange bell pepper w/o seed	Vegetables	91.00	cluster1	2.38	0.06	0.14	0.02	0.03	0.03	0.02	N.D	0.24	N.D	N.D	0.01	N.D	0.00	2.94
114	Steamed butternut squash	Vegetables	89.61	cluster1	3.44	0.28	0.63	0.05	0.04	0.01	0.02	N.D	0.30	N.D	N.D	0.04	N.D	0.01	4.81
115	Pureed butternut squash	Vegetables	89.33	cluster1	3.13	0.30	0.13	0.06	0.04	0.03	0.02	N.D	0.30	N.D	N.D	0.06	N.D	0.01	4.08
116	Steamed spaghetti squash	Vegetables	92.97	cluster1	1.72	0.15	0.11	0.06	0.03	0.02	0.02	N.D	0.25	N.D	N.D	0.04	N.D	0.01	2.42
117	Steamed yellow neck squash	Vegetables	92.77	cluster1	1.75	0.25	N.D	0.08	0.08	0.02	0.02	N.D	0.16	N.D	N.D	0.02	N.D	0.01	2.39
118	Steamed green zuchini	Vegetables	93.80	cluster1	0.84	0.12	N.D	0.07	0.06	0.02	0.01	N.D	0.14	N.D	N.D	0.02	N.D	0.02	1.29
119	Steamed mexican squash	Vegetables	93.46	cluster1	1.10	0.19	N.D	0.08	0.08	0.02	0.02	N.D	0.15	N.D	N.D	0.01	N.D	0.01	1.66
120	Steamed whole sweet potato	Vegetables	81.65	cluster1	6.25	0.33	1.21	0.05	0.18	0.01	0.04	N.D	0.48	N.D	N.D	N.D	N.D	0.01	8.55

121	Steamed sweet potato flesh only	Vegetables	82.09	cluster1	5.70	0.40	0.05	0.03	0.15	0.02	0.05	N.D	0.39	N.D	N.D	N.D	N.D	0.01	6.81
122	Whole baby bella mushrooms	Vegetables	90.61	cluster1	1.28	0.11	N.D	0.07	0.01	0.02	0.00	0.01	N.D	N.D	N.D	0.09	N.D	0.04	1.64
123	Steamed whole baby bella mushrooms	Vegetables	90.32	cluster1	1.73	0.16	N.D	0.08	0.01	0.03	0.00	0.02	N.D	N.D	N.D	0.12	N.D	0.04	2.19
124	Steamed whole crimini mushrooms	Vegetables	92.24	cluster1	0.66	0.09	N.D	0.07	0.01	0.02	0.00	0.01	N.D	N.D	N.D	0.05	N.D	0.02	0.92
125	Steamed whole white mushrooms	Vegetables	92.07	cluster1	0.93	0.13	N.D	0.05	0.01	0.02	0.00	0.01	N.D	N.D	N.D	0.07	N.D	0.02	1.25
126	Steamed whole shiitake mushrooms	Vegetables	90.18	cluster1	5.76	0.20	N.D	0.14	0.02	0.07	0.00	0.03	N.D	N.D	N.D	0.43	N.D	0.02	6.67
127	Steamed whole bunapi mushrooms	Vegetables	91.07	cluster1	2.81	0.14	N.D	0.12	0.01	0.06	0.00	0.03	0.00	N.D	N.D	0.11	N.D	0.03	3.30
128	Steamed whole beech mushrooms	Vegetables	87.33	cluster1	2.99	0.19	0.08	0.11	0.01	0.07	0.00	0.02	N.D	N.D	N.D	0.08	N.D	0.05	3.61
129	Steamed purple top turnip flesh only	Vegetables	94.03	cluster1	2.01	0.15	0.64	0.10	0.09	0.02	0.02	N.D	0.23	N.D	N.D	0.04	N.D	0.01	3.31
130	Whole English cucumber	Vegetables	95.91	cluster1	0.26	0.18	N.D	0.14	0.04	0.02	0.01	N.D	0.15	N.D	N.D	0.04	N.D	N.D	0.84
131	Steamed whole English cucumber	Vegetables	95.70	cluster1	0.96	0.17	0.10	0.10	0.05	0.02	0.01	N.D	0.12	N.D	N.D	0.02	N.D	0.01	1.56
132	Steamed whole mini cucumbers	Vegetables	94.65	cluster1	1.04	0.22	0.14	0.11	0.07	0.02	0.01	N.D	0.13	N.D	N.D	0.03	N.D	0.01	1.78

133	Steamed green cucumber flesh only	Vegetables	95.38	cluster1	1.24	0.16	0.11	0.09	0.04	0.02	0.01	N.D	0.09	N.D	N.D	0.02	N.D	0.01	1.79
134	Steamed whole green cucumber	Vegetables	95.00	cluster1	0.93	0.14	N.D	0.06	0.05	0.02	0.01	N.D	0.11	N.D	N.D	0.01	N.D	0.01	1.35
135	Creamy peanut butter (Skippy)	Beans, Peas, Legumes, Nuts, Seeds	0.50	cluster1	5.84	0.22	0.27	0.25	1.83	0.08	0.05	0.01	0.13	N.D	N.D	0.06	N.D	0.04	8.77
136	Peanut butter (Laura Scudder's)	Beans, Peas, Legumes, Nuts, Seeds	0.06	cluster1	2.87	0.32	0.06	0.20	1.52	0.11	0.04	0.00	0.13	N.D	N.D	0.05	N.D	0.04	5.35
137	Sesame seed butter (Organics)	Beans, Peas, Legumes, Nuts, Seeds	0.26	cluster1	1.61	0.52	N.D	0.23	0.58	N.D	0.03	N.D	0.11	N.D	N.D	1.34	0.02	N.D	4.44
138	Peanut butter (JIF)	Beans, Peas, Legumes, Nuts, Seeds	0.42	cluster1	4.19	0.27	N.D	0.42	0.98	0.05	0.06	N.D	0.33	N.D	N.D	N.D	0.02	N.D	6.31
139	Almond Butter (Justin's)	Beans, Peas, Legumes, Nuts, Seeds	0.66	cluster1	2.59	1.22	N.D	1.49	4.44	0.20	0.23	N.D	0.53	N.D	N.D	0.09	N.D	N.D	10.79
140	Maple almond Butter (Justin's)	Beans, Peas, Legumes, Nuts, Seeds	0.31	cluster1	5.06	1.42	N.D	1.31	4.81	0.18	0.25	N.D	0.53	N.D	N.D	N.D	0.04	N.D	13.59

141	Soy milk	Beans, Peas, Legumes, Nuts, Seeds	89.69	cluster1	1.71	0.31	0.45	N.D	0.02	N.D	0.00	N.D	0.01	N.D	N.D	0.02	N.D	0.02	2.54
142	Almond milk	Beans, Peas, Legumes, Nuts, Seeds	85.33	cluster1	0.21	0.37	0.02	0.04	0.40	0.02	0.09	0.03	0.03	N.D	N.D	0.13	N.D	0.00	1.35
143	Mango juice	Fruits	87.01	cluster1	3.55	0.13	2.24	0.06	0.13	0.03	0.01	N.D	0.19	N.D	N.D	N.D	N.D	0.00	6.35
144	Carrot juice	Vegetable s	93.93	cluster1	2.14	0.09	0.69	0.00	0.04	N.D	0.01	N.D	0.10	N.D	N.D	N.D	N.D	0.00	3.07
145	Beet juice	Vegetable s	87.58	cluster1	4.64	0.02	1.85	0.00	0.03	N.D	0.00	0.00	0.02	0.01	0.00	N.D	N.D	0.01	6.58
146	Vegetable juice	Vegetable s	94.04	cluster1	0.95	0.11	0.05	0.09	0.04	N.D	0.01	N.D	0.13	N.D	N.D	0.04	N.D	N.D	1.42
147	Apple juice	Fruits	98.26	cluster1	0.41	0.00	0.32	0.01	0.01	N.D	0.00	N.D	0.02	N.D	N.D	N.D	N.D	0.00	0.77
148	Pulp free orange juice	Fruits	90.30	cluster1	3.51	0.04	1.32	0.01	0.03	N.D	0.01	0.00	0.03	0.01	0.00	N.D	N.D	0.00	4.96
149	High pulp orange juice	Fruits	96.85	cluster1	0.79	0.09	0.31	0.03	0.11	0.01	0.03	N.D	0.21	N.D	N.D	0.01	N.D	0.00	1.59
150	Lowfat chocolate milk	Milk and Milk Products	83.19	cluster1	3.66	2.44	N.D	0.00	0.01	N.D	0.00	N.D	0.01	N.D	N.D	0.02	N.D	N.D	6.14
151	Whole milk	Milk and Milk Products	88.98	cluster1	1.74	1.94	N.D	0.00	0.00	N.D	0.00	N.D	0.00	N.D	N.D	0.01	N.D	0.01	3.70
152	Lactose-free whole milk	Milk and Milk Products	87.64	cluster1	0.64	1.40	N.D	0.00	0.00	N.D	0.00	N.D	N.D	N.D	N.D	N.D	N.D	N.D	2.04
153	Oat milk	Grain Products	90.92	cluster1	4.49	N.D	N.D	0.02	0.03	N.D	0.00	N.D	0.00	N.D	N.D	0.02	N.D	N.D	4.56
154	Non fat greek yogurt	Milk and Milk Products	84.68	cluster1	1.45	2.14	N.D	0.00	0.00	0.00	0.02	N.D	0.00	N.D	N.D	0.00	0.02	N.D	3.64

155	Blueberry greek yogurt	Milk and Milk Products	79.12	cluster1	4.99	1.66	1.92	0.02	0.02	0.00	0.02	N.D	0.06	N.D	N.D	0.02	0.01	N.D	8.72
156	Cottage cheese	Milk and Milk Products	76.99	cluster1	1.93	2.08	0.57	0.00	0.00	N.D	0.01	N.D	N.D	N.D	N.D	0.01	0.01	N.D	4.61
157	Low Moisture part Skim Mozarella cheese	Milk and Milk Products	47.84	cluster1	0.17	0.40	0.01	0.00	0.00	0.01	0.01	N.D	N.D	N.D	N.D	N.D	N.D	0.02	0.62
158	Sprouted tofu	Beans, Peas, Legumes, Nuts, Seeds	79.47	cluster1	0.25	0.31	N.D	0.01	0.08	N.D	0.03	N.D	0.05	N.D	N.D	0.12	N.D	0.03	0.88
159	Commercial tofu	Beans, Peas, Legumes, Nuts, Seeds	81.72	cluster1	1.41	0.79	N.D	1.17	0.64	0.06	0.11	N.D	0.70	N.D	N.D	0.11	0.09	N.D	5.08
160	Cinnamon Almond Butter	Beans, Peas, Legumes, Nuts, Seeds	0.25	cluster1	4.53	1.51	N.D	2.05	5.76	0.22	0.31	N.D	0.66	N.D	N.D	0.04	0.03	N.D	15.10
161	Almond flour	Beans, Peas, Legumes, Nuts, Seeds	4.53	cluster1	1.65	0.46	0.11	0.36	2.89	0.10	0.07	N.D	0.18	N.D	N.D	0.06	N.D	0.03	5.92
162	Hazelnut flour	Beans, Peas, Legumes, Nuts, Seeds	0.02	cluster1	2.90	0.54	0.26	0.19	1.67	0.09	0.07	0.01	0.13	N.D	N.D	0.04	N.D	0.04	5.93
163	Soy flour	Beans, Peas,	6.89	cluster1	1.97	11.55	N.D	1.33	3.82	0.58	0.33	0.10	0.54	N.D	N.D	1.01	0.15	N.D	21.38

		Legumes, Nuts, Seeds																	
164	Canned Artichoke hearts	Vegetable s	81.41	cluster1	1.51	0.42	0.55	0.12	0.40	0.03	0.06	0.00	0.53	N.D	N.D	0.06	N.D	0.03	3.72
165	Corn polenta	Grain Products	81.84	cluster1	6.20	0.24	2.38	0.15	0.30	0.03	0.03	N.D	0.20	N.D	N.D	N.D	0.04	N.D	9.57
166	Original apple sauce (Tree top)	Fruits	82.20	cluster1	3.77	0.25	3.33	0.27	0.24	0.07	0.03	N.D	0.42	N.D	N.D	0.01	N.D	0.00	8.39
167	No added sugar apple sauce (Tree top)	Fruits	84.00	cluster1	2.79	0.21	1.56	0.17	0.26	N.D	0.02	N.D	0.30	N.D	N.D	0.02	N.D	N.D	5.32
168	No added sugar apple sauce (Motts)	Fruits	86.26	cluster1	2.63	0.14	2.16	0.14	0.13	N.D	0.01	N.D	0.18	N.D	N.D	0.00	N.D	N.D	5.40
169	No added sugar mixed berry apple sauce (Motts)	Fruits	85.59	cluster1	2.84	0.13	1.86	0.11	0.19	N.D	0.01	N.D	0.21	N.D	N.D	0.02	N.D	N.D	5.37
170	No added sugar strawberry apple sauce (Motts)	Fruits	84.84	cluster1	3.78	0.18	2.95	0.20	0.16	0.04	0.02	N.D	0.25	0.01	0.00	0.02	N.D	0.01	7.63
171	Organic Happy Tots 287	Fruits	69.22	cluster1	3.64	0.26	4.41	0.23	0.55	0.01	0.04	0.02	0.43	N.D	N.D	N.D	N.D	0.02	9.62
172	Organic Happy Tots 288	Fruits	81.46	cluster1	5.04	0.42	2.17	1.29	0.33	N.D	0.03	N.D	0.30	N.D	N.D	0.02	N.D	N.D	9.59
173	Organic Happy Tots 289	Fruits	82.09	cluster1	2.79	0.26	1.83	0.22	0.44	N.D	0.02	N.D	0.26	N.D	N.D	0.02	N.D	N.D	5.84
174	Organic Happy Tots 290	Fruits	82.13	cluster1	2.58	0.30	1.72	0.28	0.47	N.D	0.03	N.D	0.39	N.D	N.D	0.02	N.D	N.D	5.81
175	Organic Happy Tots 291	Fruits	79.68	cluster1	1.85	0.19	1.78	0.12	0.39	N.D	0.02	N.D	0.35	N.D	N.D	N.D	N.D	N.D	4.70

176	Organic Happy Tots 292	Fruits	95.57	cluster1	0.58	0.06	0.38	0.03	0.10	N.D	0.01	N.D	0.10	N.D	N.D	0.00	N.D	N.D	1.26
177	Organic Happy Baby 294	Fruits	83.71	cluster1	2.07	0.10	0.77	0.08	0.22	0.06	0.02	0.00	0.33	N.D	N.D	0.02	N.D	0.00	3.67
178	Organic Happy Baby 295	Fruits	84.05	cluster1	3.49	0.16	2.17	0.29	0.19	0.05	0.03	N.D	0.30	0.01	0.01	0.01	N.D	0.01	6.72
179	Organic Happy Baby 296	Fruits	84.52	cluster1	4.55	0.35	1.95	0.09	0.14	N.D	0.02	N.D	0.27	N.D	N.D	0.03	N.D	N.D	7.40
180	Organic Happy Baby 297	Fruits	80.64	cluster1	2.62	0.07	0.76	0.27	0.14	0.06	0.02	N.D	0.14	N.D	N.D	N.D	N.D	0.01	4.08
181	Organic Happy Baby 299	Fruits	86.18	cluster1	2.91	0.16	2.55	0.14	0.16	N.D	0.02	N.D	0.26	N.D	N.D	0.01	N.D	N.D	6.21
182	Organic Happy Baby 300	Fruits	84.55	cluster1	1.65	0.24	0.67	0.65	0.40	0.07	0.04	N.D	0.40	0.01	0.00	0.02	N.D	0.01	4.17
183	Organic Happy Baby 303	Vegetables	87.68	cluster1	5.84	0.25	2.62	0.22	0.19	0.02	0.02	N.D	0.17	N.D	N.D	N.D	0.01	N.D	9.33
184	Organic Happy Baby 305	Vegetables	82.42	cluster1	6.32	0.33	1.11	0.14	0.22	N.D	0.02	N.D	0.32	N.D	N.D	0.08	N.D	N.D	8.54
185	Organic Happy Baby 306	Fruits	82.26	cluster1	4.75	0.74	3.16	0.11	0.31	0.02	0.06	N.D	0.63	N.D	N.D	0.03	0.01	N.D	9.83
186	Beach-Nut Naturals 307	Vegetables	91.59	cluster1	1.77	0.17	1.47	0.07	0.04	0.01	0.02	N.D	0.26	N.D	N.D	0.03	0.01	N.D	3.84
187	Beach-Nut Naturals 308	Vegetables	93.69	cluster1	3.43	0.13	0.74	0.04	0.06	0.00	0.02	N.D	0.08	N.D	N.D	N.D	0.00	N.D	4.51
188	Beach-Nut Naturals 311	Fruits	85.43	cluster1	3.36	0.13	1.70	0.15	0.15	0.05	0.02	N.D	0.26	N.D	N.D	N.D	N.D	0.00	5.83
189	Beach-Nut Naturals 313	Fruits	86.53	cluster1	5.59	0.15	3.99	0.48	0.21	0.02	0.01	N.D	0.12	N.D	N.D	N.D	0.01	N.D	10.58
190	Beach-Nut Naturals 314	Fruits	86.07	cluster1	2.92	0.19	4.55	0.16	0.16	0.03	0.02	N.D	0.20	N.D	N.D	0.03	0.00	N.D	8.26
191	Beach-Nut Naturals 315	Vegetables	93.01	cluster1	2.50	0.22	0.83	0.09	0.18	0.02	0.02	N.D	0.12	N.D	N.D	0.02	0.02	N.D	4.02
192	Beach-Nut Naturals 318	Fruits	82.89	cluster1	1.71	0.16	1.72	0.54	0.31	N.D	0.03	N.D	0.31	0.01	0.00	N.D	N.D	0.01	4.80
193	Beach-Nut Naturals 319	Vegetables	88.98	cluster1	2.40	0.33	1.32	0.09	0.17	0.01	0.03	N.D	0.22	N.D	N.D	N.D	0.01	N.D	4.58

194	Beach-Nut Naturals 320	Fruits	81.07	cluster1	5.07	0.26	1.49	0.20	0.24	N.D	0.02	N.D	0.29	N.D	N.D	0.05	N.D	N.D	7.60
195	Beach-Nut Naturals 321	Vegetables	89.37	cluster1	4.77	0.37	0.86	0.25	0.27	0.01	0.01	N.D	0.11	N.D	N.D	0.02	0.02	N.D	6.68
196	Beach-Nut Organics 323	Fruits	83.37	cluster1	2.92	0.22	4.56	0.20	0.18	0.04	0.02	N.D	0.24	N.D	N.D	0.01	0.01	N.D	8.40
197	Beach-Nut 324	Vegetables	94.00	cluster1	1.25	0.38	0.82	0.05	0.16	0.01	0.03	N.D	0.31	N.D	N.D	0.02	0.01	N.D	3.05
198	Beach-Nut 325	Fruits	82.14	cluster1	4.46	0.31	2.95	0.45	0.32	0.04	0.03	N.D	0.34	N.D	N.D	N.D	0.01	N.D	8.92
199	Beach-Nut 326	Fruits	84.77	cluster1	4.01	0.20	6.01	0.20	0.18	0.04	0.02	N.D	0.23	N.D	N.D	0.00	0.00	N.D	10.90
200	Beach-Nut Naturals 327	Fruits	78.94	cluster1	5.44	0.28	5.98	0.70	0.30	0.03	0.03	N.D	0.28	N.D	N.D	0.05	0.01	N.D	13.10
201	Beach-Nut Naturals 328	Fruits	82.19	cluster1	4.79	0.24	2.64	0.33	0.24	N.D	0.03	N.D	0.37	N.D	N.D	N.D	N.D	0.01	8.66
202	Beach-Nut Naturals 329	Vegetables	84.30	cluster1	5.23	0.17	0.90	0.05	0.06	N.D	0.01	N.D	0.21	N.D	N.D	0.05	N.D	N.D	6.69
203	Nature's Heart 330	Fruits	83.32	cluster1	3.72	0.28	4.32	0.83	0.31	0.04	0.03	N.D	0.34	N.D	N.D	0.03	0.01	N.D	9.91
204	Nature's Heart 332	Fruits	80.73	cluster1	5.84	0.11	2.93	0.34	0.18	N.D	0.02	N.D	0.30	N.D	N.D	0.01	N.D	0.01	9.74
205	Nature's Heart 333	Fruits	81.75	cluster1	4.06	0.25	1.90	0.42	0.20	N.D	0.02	N.D	0.34	N.D	N.D	0.02	N.D	N.D	7.21
206	Nature's Heart 334	Vegetables	83.31	cluster1	5.59	0.18	3.48	0.08	0.11	0.01	0.02	N.D	0.26	N.D	N.D	0.06	0.01	N.D	9.81
207	Nature's Heart 335	Fruits	86.68	cluster1	4.28	0.13	2.57	0.09	0.09	0.01	0.01	N.D	0.15	N.D	N.D	0.04	0.00	N.D	7.37
208	Plum Organics 336	Fruits	81.17	cluster1	5.53	0.18	3.20	0.08	0.32	N.D	0.03	N.D	0.45	N.D	N.D	N.D	N.D	0.01	9.80
209	Plum Organics 340	Fruits	83.39	cluster1	5.80	0.26	2.55	0.44	0.26	0.03	0.02	N.D	0.22	N.D	N.D	N.D	0.01	N.D	9.59
210	Plum Organics 342	Fruits	81.77	cluster1	2.01	0.18	1.95	0.85	0.37	N.D	0.02	N.D	0.28	N.D	N.D	N.D	N.D	N.D	5.65
211	Plum Organics 343	Fruits	83.96	cluster1	2.26	0.29	1.32	0.83	0.30	N.D	0.02	N.D	0.30	N.D	N.D	0.01	N.D	N.D	5.33
212	Plum Organics 344	Fruits	86.16	cluster1	3.01	0.30	2.47	0.15	0.19	0.03	0.03	N.D	0.29	N.D	N.D	N.D	0.01	N.D	6.47

213	Plum Organics 346	Vegetables	87.11	cluster1	5.44	0.24	0.84	0.20	0.27	0.01	0.03	N.D	0.12	N.D	N.D	N.D	0.02	N.D	7.16
214	Plum Organics 349	Fruits	85.54	cluster1	3.87	0.49	2.11	0.30	0.23	0.02	0.02	N.D	0.18	N.D	N.D	0.02	0.01	N.D	7.24
215	Plum Organics 350	Fruits	82.63	cluster1	6.26	0.36	1.08	0.06	0.09	N.D	0.01	N.D	0.15	N.D	N.D	0.03	N.D	N.D	8.03
216	Plum Organics 351	Fruits	83.96	cluster1	5.09	0.31	2.67	0.99	0.44	0.03	0.04	N.D	0.25	N.D	N.D	0.05	0.01	N.D	9.88
217	Matene 352	Fruits	81.35	cluster1	4.13	0.24	3.00	0.22	0.17	N.D	0.02	N.D	0.26	N.D	N.D	0.01	N.D	N.D	8.06
218	Matene 353	Fruits	82.46	cluster1	2.01	0.17	3.34	0.22	0.13	0.03	0.02	N.D	0.25	N.D	N.D	N.D	0.00	N.D	6.16
219	Matene 354	Fruits	85.26	cluster1	4.23	0.14	3.03	0.13	0.14	N.D	0.02	N.D	0.24	0.01	0.00	N.D	N.D	0.01	7.94
220	Matene 355	Fruits	80.74	cluster1	4.26	0.29	3.13	0.33	0.30	N.D	0.04	N.D	0.48	N.D	N.D	N.D	N.D	0.01	8.84
221	Matene 356	Fruits	82.01	cluster1	3.54	0.14	2.94	0.15	0.14	N.D	0.01	N.D	0.21	N.D	N.D	N.D	N.D	N.D	7.14
222	Matene 357	Fruits	84.13	cluster1	4.24	0.18	1.44	0.20	0.17	N.D	0.02	N.D	0.30	N.D	N.D	0.03	N.D	0.01	6.59
223	Paw Patrol 358	Fruits	79.98	cluster1	5.68	0.14	1.33	0.11	0.11	N.D	0.02	N.D	0.27	N.D	N.D	0.05	N.D	0.01	7.72
224	Paw Patrol 359	Fruits	84.13	cluster1	3.70	0.21	2.81	0.22	0.20	N.D	0.01	N.D	0.27	N.D	N.D	0.02	N.D	N.D	7.44
225	Paw Patrol 360	Fruits	80.13	cluster1	5.65	0.12	1.65	0.23	0.13	N.D	0.02	N.D	0.23	N.D	N.D	0.00	N.D	0.00	8.06
226	Organics 361	Fruits	84.82	cluster1	4.94	0.27	1.84	0.18	0.21	0.03	0.03	N.D	0.33	N.D	N.D	0.03	0.00	N.D	7.86
227	Organics 362	Fruits	84.25	cluster1	5.30	0.22	1.67	0.21	0.19	N.D	0.02	N.D	0.22	N.D	N.D	0.05	N.D	N.D	7.88
228	Organics 363	Vegetables	78.21	cluster1	4.42	0.20	1.53	0.11	0.14	0.07	0.02	N.D	0.42	N.D	N.D	0.05	N.D	0.00	6.96
229	Organics 367	Fruits	93.40	cluster1	0.77	0.01	0.25	0.04	0.04	0.01	0.00	N.D	0.04	N.D	N.D	0.01	N.D	0.00	1.17
230	Organics 368	Fruits	85.75	cluster1	3.17	0.19	2.03	0.15	0.17	N.D	0.02	N.D	0.27	0.01	0.00	N.D	N.D	0.00	6.02
231	Organics 369	Vegetables	86.74	cluster1	4.41	0.32	1.01	0.16	0.16	0.03	0.03	N.D	0.27	N.D	N.D	0.02	0.01	N.D	6.40
232	Pumpkin Tree 371	Fruits	85.19	cluster1	2.46	0.20	3.20	0.14	0.13	0.02	0.03	N.D	0.23	N.D	N.D	0.02	0.01	N.D	6.43
233	Pumpkin Tree 373	Fruits	81.75	cluster1	4.13	0.13	2.96	0.09	0.09	0.01	0.01	N.D	0.20	N.D	N.D	N.D	0.01	N.D	7.63
234	Pumpkin Tree 374	Fruits	77.56	cluster1	2.85	0.36	2.44	0.91	0.65	N.D	0.03	N.D	0.53	N.D	N.D	0.10	N.D	N.D	7.87
235	Pumpkin Tree 375	Fruits	83.93	cluster1	4.94	0.26	1.60	0.19	0.28	0.03	0.02	N.D	0.25	N.D	N.D	0.03	0.03	N.D	7.63
236	Pumpkin Tree 376	Fruits	81.18	cluster1	5.47	0.30	2.78	0.12	0.22	0.03	0.02	N.D	0.31	N.D	N.D	0.03	0.02	N.D	9.29
237	Gerber Natural 378	Fruits	83.50	cluster1	4.49	0.19	2.37	0.16	0.16	N.D	0.03	N.D	0.34	N.D	N.D	0.01	N.D	0.01	7.75

238	Gerber Natural 380	Fruits	84.58	cluster1	2.19	0.26	1.46	0.50	0.37	N.D	0.02	N.D	0.31	N.D	N.D	0.03	N.D	N.D	5.14
239	Geber Organics 382	Fruits	81.61	cluster1	5.62	0.24	5.88	0.13	0.18	0.04	0.02	N.D	0.25	N.D	N.D	0.03	0.01	N.D	12.40
240	Geber Organics 383	Fruits	85.81	cluster1	2.92	0.15	4.86	0.26	0.14	0.03	0.02	N.D	0.23	N.D	N.D	N.D	0.00	N.D	8.61
241	Geber Organics 384	Vegetables	85.49	cluster1	3.53	0.24	2.50	0.17	0.26	N.D	0.04	N.D	0.49	N.D	N.D	0.03	N.D	0.01	7.28
242	Geber Organics 385	Vegetables	84.06	cluster1	4.77	0.19	2.99	0.08	0.09	0.01	0.02	N.D	0.23	N.D	N.D	0.08	0.01	N.D	8.46
243	Geber Organics 386	Fruits	85.14	cluster1	2.04	0.15	2.05	0.21	0.28	N.D	0.03	N.D	0.38	0.01	0.00	N.D	N.D	0.00	5.15
244	Geber Organics 387	Fruits	84.96	cluster1	3.48	0.23	2.91	0.17	0.17	0.03	0.02	N.D	0.22	N.D	N.D	0.01	0.01	N.D	7.25
245	Geber Organics 388	Fruits	81.44	cluster1	2.03	0.20	3.25	1.20	0.42	N.D	0.05	N.D	0.42	N.D	N.D	N.D	N.D	0.01	7.57
246	Geber Organics 391	Fruits	83.23	cluster1	3.27	0.18	3.14	0.19	0.15	0.04	0.02	N.D	0.27	N.D	N.D	0.00	0.00	N.D	7.28
247	Geber Organics 392	Fruits	85.64	cluster1	2.03	0.24	2.38	0.47	0.32	0.04	0.02	N.D	0.29	N.D	N.D	0.03	0.01	N.D	5.82
248	Geber Organics 393	Fruits	81.92	cluster1	2.21	0.21	2.21	0.77	0.25	0.03	0.03	N.D	0.31	N.D	N.D	N.D	0.01	N.D	6.04
249	Geber Organics 394	Fruits	77.39	cluster1	3.59	0.32	2.53	0.51	0.53	N.D	0.03	N.D	0.44	N.D	N.D	0.06	N.D	N.D	8.00
250	Geber Organics 395	Vegetables	86.00	cluster1	2.12	0.11	1.00	0.09	0.13	0.04	0.02	0.00	0.31	N.D	N.D	0.02	N.D	0.00	3.84
251	Geber Organics 396	Fruits	76.29	cluster1	4.32	0.35	1.64	0.07	0.23	0.08	0.03	0.00	0.51	N.D	N.D	N.D	N.D	0.01	7.23
252	Geber Organics 398	Fruits	86.17	cluster1	2.30	0.19	2.17	0.29	0.15	0.03	0.02	N.D	0.23	N.D	N.D	N.D	0.00	N.D	5.39
253	Geber Organics 401	Fruits	93.41	cluster1	1.72	0.19	0.93	0.16	0.13	N.D	0.01	N.D	0.15	N.D	N.D	0.03	N.D	N.D	3.31
254	Gerber 402	Fruits	84.62	cluster1	2.30	0.30	2.13	0.82	0.44	N.D	0.02	N.D	0.38	N.D	N.D	0.07	N.D	N.D	6.47
255	Gerber 407	Fruits	88.56	cluster1	3.88	0.55	1.06	0.09	0.09	N.D	0.01	N.D	0.16	N.D	N.D	0.00	N.D	N.D	5.84
256	Gerber Organic 408	Fruits	85.91	cluster1	2.86	0.20	0.66	0.21	0.19	N.D	0.02	N.D	0.21	N.D	N.D	0.05	N.D	N.D	4.39

257	Gerber Organic 409	Fruits	71.69	cluster1	4.31	0.14	2.53	0.23	0.18	N.D	0.04	N.D	0.39	N.D	N.D	N.D	N.D	0.01	7.82
258	Gerber 412	Vegetables	84.81	cluster1	4.96	0.26	N.D	0.19	0.19	N.D	0.02	N.D	0.21	N.D	N.D	0.05	N.D	N.D	5.87
259	Gerber 413	Fruits	79.80	cluster1	6.00	0.25	3.41	0.25	0.21	N.D	0.04	N.D	0.38	0.02	0.01	0.04	N.D	0.01	10.61
260	Gerber 414	Fruits	86.27	cluster1	2.49	0.17	1.48	0.24	0.14	N.D	0.03	N.D	0.28	N.D	N.D	0.00	N.D	0.01	4.82
261	Gerber 418	Fruits	83.00	cluster1	3.77	0.21	2.72	0.47	0.29	N.D	0.03	N.D	0.34	N.D	N.D	0.04	N.D	0.01	7.89
262	Beach-Nut Naturals 419	Vegetables	89.47	cluster1	5.34	0.33	N.D	0.14	0.40	N.D	0.02	N.D	0.13	N.D	N.D	0.07	N.D	N.D	6.41
263	Golden flaxseed	Beans, Peas, Legumes, Nuts, Seeds	5.13	cluster1	7.03	3.41	N.D	4.10	4.29	N.D	0.83	N.D	1.75	N.D	N.D	0.43	N.D	0.07	21.91
264	Hummus spread	Beans, Peas, Legumes, Nuts, Seeds	64.78	cluster1	4.99	0.19	0.18	0.06	0.95	0.02	0.01	0.00	0.03	N.D	N.D	0.08	N.D	0.01	6.53
265	Tahini spread (365 Whole Foods Market)	Beans, Peas, Legumes, Nuts, Seeds	16.11	cluster1	1.14	0.53	0.25	0.16	0.94	0.04	0.03	0.00	0.02	N.D	N.D	0.98	N.D	0.03	4.12
266	Tahini spread (Once Again)	Beans, Peas, Legumes, Nuts, Seeds	0.12	cluster1	0.52	0.27	0.06	0.20	1.11	0.04	0.03	0.00	0.04	N.D	N.D	0.70	N.D	0.03	3.01
267	Almond butter (MaraNatha)	Beans, Peas, Legumes, Nuts, Seeds	2.59	cluster1	3.93	0.39	0.91	0.36	3.25	0.11	0.06	0.00	0.13	N.D	N.D	0.05	N.D	0.03	9.23
268	Almond butter (Barney)	Beans, Peas, Legumes,	0.50	cluster1	3.09	0.44	0.81	0.30	2.81	0.10	0.05	0.00	0.10	N.D	N.D	0.06	N.D	0.03	7.80

		Nuts, Seeds																	
269	Almond butter (Artisana Organics)	Beans, Peas, Legumes, Nuts, Seeds	1.21	cluster1	1.65	0.43	0.34	0.30	2.34	0.10	0.05	0.00	0.09	N.D	N.D	0.06	N.D	0.03	5.41
270	Tahini spread (Kevala)	Beans, Peas, Legumes, Nuts, Seeds	0.02	cluster1	1.47	0.73	0.22	0.20	1.30	0.03	0.04	0.00	0.04	N.D	N.D	0.94	N.D	0.04	5.02
271	Tahini spread (Artisana Organics)	Beans, Peas, Legumes, Nuts, Seeds	4.69	cluster1	1.43	0.63	0.23	0.18	0.92	0.03	0.03	0.01	0.03	N.D	N.D	0.68	N.D	0.04	4.21
272	Almond butter (Once again)	Beans, Peas, Legumes, Nuts, Seeds	0.20	cluster1	2.30	0.54	0.64	0.38	3.20	0.12	0.06	0.01	0.13	N.D	N.D	0.06	N.D	0.04	7.48
273	Almon butter (Alive & Organics)	Beans, Peas, Legumes, Nuts, Seeds	1.37	cluster1	2.55	0.68	0.77	0.41	3.14	0.11	0.06	0.01	0.13	N.D	N.D	0.07	N.D	0.03	7.95
274	Black tahini spread	Beans, Peas, Legumes, Nuts, Seeds	0.53	cluster1	1.24	0.74	0.26	0.30	1.03	0.03	0.05	0.01	0.04	N.D	N.D	0.58	N.D	0.04	4.32
275	Dark roasted peanut butter	Beans, Peas, Legumes, Nuts, Seeds	0.23	cluster1	4.84	0.38	0.61	0.22	1.39	0.07	0.03	0.00	0.07	N.D	N.D	0.05	N.D	0.04	7.69

276	Coconut beverage	Beans, Peas, Legumes, Nuts, Seeds	96.77	cluster1	0.13	0.04	0.04	0.00	0.00	0.00	0.00	0.00	0.00	N.D	N.D	0.01	N.D	0.00	0.21
277	Hemp beverage	Beans, Peas, Legumes, Nuts, Seeds	95.99	cluster1	0.07	0.03	0.01	0.00	0.03	0.01	0.00	0.00	0.00	N.D	N.D	0.01	N.D	0.00	0.17
278	Macadamia milk	Beans, Peas, Legumes, Nuts, Seeds	97.58	cluster1	0.03	0.01	0.01	0.00	0.03	0.00	0.00	0.00	0.00	N.D	N.D	0.03	N.D	0.00	0.12
279	Almond and cashew beverage with cocoa	Beans, Peas, Legumes, Nuts, Seeds	92.37	cluster1	1.87	0.02	0.39	0.01	0.10	0.01	0.00	0.00	0.01	N.D	N.D	0.05	N.D	0.00	2.47
280	Almon, cashew and macademia beverage	Beans, Peas, Legumes, Nuts, Seeds	97.58	cluster1	0.12	0.02	0.01	0.00	0.04	0.00	0.00	0.00	0.00	N.D	N.D	0.03	N.D	0.00	0.23
281	Hemp seeds	Beans, Peas, Legumes, Nuts, Seeds	5.48	cluster1	1.10	0.62	N.D	0.13	0.63	N.D	0.10	N.D	0.24	N.D	N.D	N.D	N.D	0.10	2.91
282	Dried coconut chips	Fruits	2.41	cluster1	3.45	1.28	0.06	N.D	0.38	N.D	0.05	N.D	0.14	N.D	N.D	7.42	N.D	0.01	12.80
283	Roasted seaweed	Vegetables	1.24	cluster1	0.19	7.59	0.06	0.31	0.01	0.07	N.D	0.01	N.D	N.D	N.D	0.74	N.D	0.18	9.16
284	Peanuts	Beans, Peas, Legumes,	0.68	cluster1	4.94	0.48	0.54	0.31	2.32	0.11	0.05	N.D	0.10	N.D	N.D	0.07	N.D	0.04	8.96

		Nuts, Seeds																	
285	Pistachio	Beans, Peas, Legumes, Nuts, Seeds	2.61	cluster1	4.25	1.29	0.93	0.17	1.96	0.10	0.05	0.01	0.09	N.D	N.D	0.06	N.D	0.05	8.96
286	Peanuts (Sunfood Superfoods)	Beans, Peas, Legumes, Nuts, Seeds	2.54	cluster1	5.01	0.49	0.59	0.26	1.79	0.10	0.03	0.00	0.06	N.D	N.D	0.06	N.D	0.03	8.44
287	Macadamia (Sunfood Superfood)	Beans, Peas, Legumes, Nuts, Seeds	0.82	cluster1	4.17	0.26	0.96	0.23	1.50	0.10	0.04	N.D	0.07	N.D	N.D	0.06	N.D	0.03	7.43
288	Apricots w/ seeds	Beans, Peas, Legumes, Nuts, Seeds	4.91	cluster1	2.99	0.54	0.88	0.46	4.11	0.16	0.10	0.01	0.18	N.D	N.D	0.12	N.D	0.03	9.57
289	Brazil nuts	Beans, Peas, Legumes, Nuts, Seeds	1.68	cluster1	1.15	0.29	0.30	0.16	0.72	0.07	0.03	0.00	0.04	N.D	N.D	0.05	N.D	0.02	2.83
290	Pistachio (Fiddyment Farm)	Beans, Peas, Legumes, Nuts, Seeds	1.66	cluster1	3.80	1.27	0.67	0.18	2.14	0.10	0.06	0.00	0.10	N.D	N.D	0.05	N.D	0.05	8.43
291	Pine nut	Beans, Peas, Legumes, Nuts, Seeds	0.86	cluster1	5.41	0.49	0.62	0.16	1.22	0.09	0.03	0.01	0.04	N.D	N.D	0.04	N.D	0.04	8.13

292	Pecan	Beans, Peas, Legumes, Nuts, Seeds	2.52	cluster1	2.16	0.37	0.43	0.24	0.75	0.08	0.04	0.00	0.07	N.D	N.D	0.04	N.D	0.03	4.21
293	Sunflower seeds	Beans, Peas, Legumes, Nuts, Seeds	3.85	cluster1	1.44	0.64	0.34	0.26	2.18	0.08	0.04	0.01	0.08	N.D	N.D	0.18	N.D	0.05	5.31
294	Pumpkin seeds	Beans, Peas, Legumes, Nuts, Seeds	5.29	cluster1	1.32	0.62	0.20	0.65	0.61	0.06	0.03	0.00	0.06	N.D	N.D	0.02	N.D	0.04	3.62
295	Minestrone soup	Vegetables	90.27	cluster1	5.01	0.24	0.13	0.07	0.13	N.D	0.02	N.D	0.12	N.D	N.D	0.01	N.D	0.01	5.74
296	Quinoa kale red lentil soup	Vegetables	87.80	cluster1	5.75	0.30	0.22	0.05	0.19	N.D	0.03	N.D	0.20	N.D	N.D	0.02	N.D	0.02	6.79
297	Frozen peach flesh	Fruits	86.97	cluster1	4.22	0.18	0.18	0.11	0.27	N.D	0.03	N.D	0.36	N.D	N.D	0.03	N.D	0.01	5.39
298	Frozen whole blueberry	Fruits	84.42	cluster1	4.50	0.24	0.13	0.22	0.16	N.D	0.03	N.D	0.25	N.D	N.D	0.02	N.D	0.00	5.56
299	Frozen whole strawberry	Fruits	89.37	cluster1	2.91	0.07	0.13	0.14	0.06	N.D	0.03	N.D	0.27	N.D	N.D	0.02	N.D	0.01	3.64
300	Frozen whole raspberry	Fruits	84.90	cluster1	2.57	0.20	0.14	0.15	0.16	N.D	0.03	N.D	0.28	N.D	N.D	0.02	N.D	0.01	3.56
301	Frozen cauliflower	Vegetables	93.30	cluster1	1.30	0.29	0.24	0.11	0.32	N.D	0.05	N.D	0.32	N.D	N.D	0.05	N.D	0.02	2.70
302	Frozen sweet pea	Beans, Peas, Legumes, Nuts, Seeds	77.58	cluster1	5.98	0.47	0.27	0.11	0.66	N.D	0.04	N.D	0.16	N.D	N.D	N.D	N.D	0.05	7.73
303	Frozen spinach leaves	Vegetables	91.20	cluster1	0.29	0.24	0.13	0.07	0.33	N.D	0.08	N.D	0.31	N.D	N.D	0.01	N.D	0.02	1.48

304	Frozen whole okra	Vegetables	91.96	cluster1	1.06	0.16	0.17	0.11	0.10	N.D	0.07	N.D	0.40	N.D	N.D	0.02	N.D	0.02	2.10
305	Frizen whole kale	Vegetables	91.21	cluster1	0.94	0.28	0.08	0.11	0.20	N.D	0.06	N.D	0.50	N.D	N.D	0.01	N.D	0.03	2.20
306	Frozen edamame	Beans, Peas, Legumes, Nuts, Seeds	72.92	cluster1	3.49	1.15	0.22	0.13	0.51	N.D	0.08	N.D	0.21	N.D	N.D	0.15	N.D	0.05	6.00
307	Frozen whole green beans	Vegetables	90.75	cluster1	1.96	0.51	0.16	0.11	0.17	N.D	0.05	N.D	0.58	N.D	N.D	0.06	N.D	0.02	3.62
308	Frozen whole brussel sprouts	Vegetables	87.18	cluster1	2.52	0.72	0.19	0.12	0.85	N.D	0.10	N.D	0.59	N.D	N.D	0.06	N.D	0.05	5.21
309	Frozen broccoli florets	Vegetables	89.17	cluster1	1.00	0.51	0.13	0.17	0.63	N.D	0.08	N.D	0.49	N.D	N.D	0.03	N.D	0.05	3.09
310	Dragon fruit flesh and seeds	Fruits	80.48	cluster1	2.66	0.38	0.58	0.58	0.55	N.D	0.05	N.D	0.09	N.D	N.D	N.D	N.D	0.01	4.91
311	Wheat gluten burger patty	Grain Products	59.93	cluster1	3.22	0.09	0.20	0.11	0.08	N.D	0.00	N.D	0.01	N.D	N.D	N.D	N.D	0.01	3.72
312	Ground non-meat (Quorn)	Vegetables	71.12	cluster1	2.57	0.16	0.07	0.00	0.01	N.D	N.D	0.04	N.D	N.D	N.D	0.46	N.D	0.05	3.36
313	Plant based burger patty	Beans, Peas, Legumes, Nuts, Seeds	54.07	cluster1	2.04	1.08	0.54	0.15	0.38	N.D	0.05	N.D	0.13	N.D	N.D	N.D	N.D	0.07	4.44
314	Ground non-meat (Beyond meat)	Beans, Peas, Legumes, Nuts, Seeds	51.40	cluster1	2.23	0.23	N.D	0.02	0.14	0.04	0.01	0.00	0.03	N.D	N.D	0.02	N.D	0.04	2.77
315	Naked Juice Power Machine	Fruits	88.07	cluster1	2.51	0.06	0.12	0.08	0.12	0.06	0.01	0.00	0.11	N.D	N.D	0.01	N.D	0.00	3.10
316	Naked Juice Green Machine	Fruits	92.05	cluster1	2.10	0.05	0.16	0.02	0.04	0.02	0.00	0.00	0.06	N.D	N.D	0.01	N.D	0.00	2.44
317	Naked Juice Orange Carrot	Fruits	86.91	cluster1	3.31	0.11	0.19	0.02	0.08	0.05	0.01	0.00	0.08	N.D	N.D	0.02	N.D	0.00	3.87

318	Naked Juice Orange Mango	Fruits	86.83	cluster1	3.08	0.08	0.12	0.03	0.07	0.05	0.01	0.00	0.07	N.D	N.D	0.00	N.D	0.00	3.52
319	Naked Juice Pina Colada	Fruits	86.24	cluster1	3.87	0.08	0.18	0.02	0.04	0.05	0.00	0.00	0.04	N.D	N.D	0.03	N.D	0.00	4.31
320	Naked Juice Red Machine	Fruits	88.78	cluster1	2.53	0.10	0.39	0.07	0.05	N.D	0.01	N.D	0.09	N.D	N.D	N.D	N.D	0.00	3.23
321	Naked Juice Mighty Mango	Fruits	85.85	cluster1	3.28	0.04	0.79	0.04	0.11	N.D	0.01	N.D	0.19	N.D	N.D	N.D	N.D	0.00	4.46
322	Naked Juice Tropical Guava	Fruits	85.49	cluster1	3.83	0.13	0.21	0.06	0.07	0.03	0.01	0.00	0.08	N.D	N.D	0.01	N.D	0.00	4.43
323	Naked Juice Kale Blazer	Fruits	90.06	cluster1	3.16	0.16	0.96	0.03	0.08	N.D	0.03	N.D	0.13	N.D	N.D	0.01	N.D	0.01	4.57
324	Naked Juice Plant Protein Peach Mango	Fruits	84.21	cluster1	4.03	0.18	1.33	0.05	0.10	N.D	0.01	N.D	0.10	N.D	N.D	0.02	N.D	0.02	5.82
325	Naked Juice Blue Machine	Fruits	84.87	cluster1	4.48	0.07	1.00	0.05	0.04	N.D	0.01	N.D	0.16	N.D	N.D	N.D	N.D	0.00	5.80
326	Naked Juice Rainbow Machine	Vegetables	85.98	cluster1	3.92	0.28	0.48	0.03	0.13	N.D	0.02	N.D	0.16	N.D	N.D	N.D	N.D	0.00	5.01
327	Naked Juice Berry Veggie	Fruits	87.49	cluster1	3.80	0.18	0.73	0.07	0.08	N.D	0.01	N.D	0.18	N.D	N.D	N.D	N.D	0.00	5.05
328	Naked Juice Strawberry Banana	Fruits	87.19	cluster1	4.29	0.09	0.63	0.06	0.04	N.D	0.01	N.D	0.19	N.D	N.D	N.D	N.D	0.00	5.32
329	Chicken Noodle Soup w/ Chicken Bone Broth (Pacifiic Foods)	Vegetables	89.16	cluster1	1.86	0.11	0.02	0.02	0.05	0.01	0.01	0.00	0.04	N.D	N.D	0.01	N.D	0.01	2.13
330	Butternut Squah Soup (Pacific Foods)	Vegetables	92.76	cluster1	2.18	0.11	0.07	0.02	0.03	0.02	0.01	0.00	0.08	N.D	N.D	0.03	N.D	0.00	2.56
331	Chicken & Wild Rice Soup	Vegetables	90.85	cluster1	4.90	0.11	0.06	0.06	0.06	0.01	0.01	0.00	0.05	N.D	N.D	0.01	N.D	0.01	5.27
332	Roasted Red Pepper Tomato	Vegetables	87.94	cluster1	2.18	0.16	0.09	0.02	0.02	0.01	0.01	0.00	0.12	N.D	N.D	0.02	N.D	0.00	2.64

333	Thai Sweet Potato Soup	Vegetables	89.27	cluster1	2.35	0.18	0.06	0.02	0.07	0.00	0.01	0.00	0.11	N.D	N.D	0.10	N.D	0.00	2.92
334	Creamy Cashew Carrot Ginger Soup	Vegetables	92.84	cluster1	1.74	0.12	0.01	0.02	0.05	0.01	0.01	0.00	0.06	N.D	N.D	0.09	N.D	0.00	2.12
335	Southwest Style Chicken Tortilla Soup	Meat, Poultry, Fish, and Mixtures	87.20	cluster1	3.78	0.16	N.D	0.07	0.17	0.02	0.01	0.00	0.08	N.D	N.D	0.06	N.D	0.01	4.36
336	Poblano Pepper and Corn Chowder	Vegetables	86.57	cluster1	4.82	0.24	N.D	0.05	0.09	0.01	0.01	0.00	0.03	N.D	N.D	0.01	N.D	0.00	5.26
337	Chicken Noodle Soup (Pacific Foods)	Vegetables	90.13	cluster1	4.22	0.09	0.03	0.04	0.07	0.00	0.00	0.00	0.03	N.D	N.D	0.01	N.D	0.00	4.51
338	Vegetable Lentil & Roasted Red Pepper Soup	Vegetables	86.64	cluster1	5.34	0.45	0.08	0.09	0.37	0.02	0.02	0.00	0.11	N.D	N.D	0.06	N.D	0.01	6.55
339	Tomato Creamy Soup (Imagine)	Vegetables	93.03	cluster1	2.40	0.11	0.10	0.02	0.03	0.00	0.01	0.00	0.11	N.D	N.D	0.03	N.D	0.00	2.82
340	Portobello Mushroom Creamy Soup (Imagine)	Vegetables	95.41	cluster1	1.97	0.06	0.03	0.00	0.01	0.00	0.00	0.00	0.02	N.D	N.D	0.00	N.D	0.00	2.12
341	Garden Tomato Cream w/ Light Sodium (Imagine)	Vegetables	93.61	cluster1	1.93	0.09	0.05	0.01	0.03	0.00	0.01	0.00	0.11	N.D	N.D	0.02	N.D	0.00	2.24
342	Butternut Squash Creamy Soup w/ Light Sodium (Imagine)	Vegetables	87.57	cluster1	3.56	0.18	0.02	0.04	0.05	0.02	0.02	0.00	0.15	N.D	N.D	0.05	N.D	0.00	4.09

343	Tomato Basil Creamy Soup (Imagine)	Vegetables	93.19	cluster1	2.05	0.09	0.02	0.02	0.03	0.01	0.01	0.00	0.10	N.D	N.D	0.02	N.D	0.00	2.35
344	Potato Leak Creamy Soup	Vegetables	94.20	cluster1	2.62	0.11	0.00	0.00	0.02	0.00	0.00	0.00	0.03	N.D	N.D	N.D	N.D	0.00	2.79
345	Broccoli Creamy Soup	Vegetables	95.12	cluster1	1.97	0.09	0.01	0.01	0.05	0.00	0.01	0.00	0.06	N.D	N.D	0.01	N.D	0.00	2.22
346	Super Greens Creamy Soup	Vegetables	94.02	cluster1	1.71	0.10	0.04	0.01	0.08	0.01	0.01	0.00	0.07	N.D	N.D	0.01	N.D	0.00	2.05
347	Whole Ground Flaxseed Meal	Beans, Peas, Legumes, Nuts, Seeds	7.18	cluster1	2.77	2.53	0.45	2.06	2.30	0.38	0.43	0.01	0.55	N.D	N.D	0.05	N.D	0.05	11.57
348	Vegi Soup Mix	Beans, Peas, Legumes, Nuts, Seeds	72.15	cluster1	6.46	0.35	0.14	0.04	1.62	0.01	0.02	0.00	0.00	N.D	N.D	0.01	N.D	0.02	8.67
349	Instant Miso-Cup Soup (w/ Seaweed)	Beans, Peas, Legumes, Nuts, Seeds	95.11	cluster1	0.12	0.02	0.00	0.01	0.07	0.01	0.00	0.00	0.00	N.D	N.D	0.00	N.D	0.00	0.24
350	Cream of Cauliflower Soup	Vegetables	90.22	cluster1	4.99	0.16	0.01	0.01	0.06	0.01	0.01	0.00	0.03	N.D	N.D	0.01	N.D	0.00	5.28
351	Extra Firm Tofu (WIldwood)	Beans, Peas, Legumes, Nuts, Seeds	83.67	cluster1	0.29	0.16	N.D	0.01	0.03	0.01	0.01	0.01	0.03	N.D	N.D	0.08	N.D	0.02	0.66
352	Firm Tofu (Wildwood)	Beans, Peas, Legumes, Nuts, Seeds	83.64	cluster1	0.17	0.16	N.D	0.01	0.04	0.01	0.01	0.00	0.03	N.D	N.D	0.06	N.D	0.02	0.52

353	High Protein Super Firm Tofu	Beans, Peas, Legumes, Nuts, Seeds	75.93	cluster1	0.30	0.29	N.D	0.01	0.06	0.02	0.02	0.01	0.04	N.D	N.D	0.12	N.D	0.02	0.89
354	Firm Tofu (Nasoya)	Beans, Peas, Legumes, Nuts, Seeds	80.63	cluster1	0.29	0.16	0.22	0.01	0.07	0.01	0.02	0.00	0.04	0.01	0.00	0.04	N.D	0.02	0.90
355	Medium Firm Tofu (House Foods)	Beans, Peas, Legumes, Nuts, Seeds	84.77	cluster1	0.56	0.34	0.08	0.00	0.04	0.00	0.01	0.00	0.02	N.D	N.D	0.07	N.D	0.02	1.14
356	Firm Tofu (House Foods)	Beans, Peas, Legumes, Nuts, Seeds	84.13	cluster1	0.49	0.20	0.06	0.00	0.04	0.01	0.01	0.00	0.02	N.D	N.D	0.08	N.D	0.02	0.93
357	Extra Firm Tofu (Nasoya)	Beans, Peas, Legumes, Nuts, Seeds	81.06	cluster1	0.40	0.16	0.10	0.01	0.04	0.01	0.02	0.01	0.03	N.D	N.D	0.08	N.D	0.01	0.87
358	Extra Firm Tofu (House Foods)	Beans, Peas, Legumes, Nuts, Seeds	80.43	cluster1	0.26	0.19	0.11	0.00	0.05	0.01	0.01	0.01	0.02	N.D	N.D	0.05	N.D	0.02	0.74
359	Extra Firm Silken Tofu	Beans, Peas, Legumes, Nuts, Seeds	87.50	cluster1	0.27	0.17	0.07	0.00	0.03	0.01	0.01	0.01	0.01	N.D	N.D	0.05	N.D	0.02	0.64
360	Soft Silken Tofu	Beans, Peas, Legumes,	89.50	cluster1	0.62	0.33	0.23	0.00	0.02	0.00	0.01	0.00	0.01	N.D	N.D	0.05	N.D	0.01	1.28

		Nuts, Seeds																	
361	Firm Silken Tofu	Beans, Peas, Legumes, Nuts, Seeds	86.97	cluster1	0.34	0.31	0.13	0.02	0.02	0.01	0.01	0.01	0.01	N.D	N.D	0.03	N.D	0.01	0.90
362	Frozen Broccoli Cuts (Birds Eye)	Vegetables	89.68	cluster1	1.02	0.30	0.18	0.04	0.42	0.02	0.04	0.00	0.31	N.D	N.D	0.04	N.D	0.03	2.40
363	Frozen Spinach Leaf Cut (Best Yet)	Vegetables	90.73	cluster1	0.37	0.19	0.11	0.04	0.24	0.01	0.04	0.00	0.18	N.D	N.D	0.01	N.D	0.02	1.20
364	Frozen Whole Green Beans (Birds Eye)	Vegetables	78.26	cluster1	3.93	0.58	1.22	0.07	0.22	0.02	0.03	0.00	0.62	0.01	0.00	0.13	N.D	0.02	6.87
365	Frozen Broccoli Cuts (Flav R Pac)	Vegetables	89.12	cluster1	1.33	0.32	0.20	0.06	0.49	0.02	0.05	0.01	0.30	N.D	N.D	0.05	N.D	0.03	2.86
366	Frozen Cauliflower Florets (Flav R Pac)	Vegetables	93.10	cluster1	0.91	0.18	0.06	0.03	0.25	0.01	0.02	N.D	0.21	N.D	N.D	0.03	N.D	0.01	1.73
367	Frozen Chopped Collard Greens	Vegetables	90.50	cluster1	0.65	0.32	0.05	0.10	0.28	0.03	0.05	0.00	0.38	0.00	0.00	0.02	N.D	0.02	1.90
368	Frozen Whole Green beans (Birds Eye)	Vegetables	87.62	cluster1	2.07	0.40	0.06	0.06	0.22	0.02	0.04	0.00	0.55	N.D	N.D	0.11	N.D	0.02	3.54
369	Frozen Whole Crinkle Cut Carrots	Vegetables	89.17	cluster1	2.99	0.54	0.13	0.02	0.29	0.03	0.05	0.00	0.40	N.D	N.D	0.03	N.D	0.01	4.49
370	Frozen Chopped Baby Spinach (Birds Eye)	Vegetables	87.62	cluster1	0.45	0.24	0.03	0.05	0.24	0.02	0.06	0.00	0.25	N.D	N.D	0.02	N.D	0.01	1.36
371	Frozen Chopped	Vegetables	89.17	cluster1	0.24	0.13	0.04	0.03	0.15	0.01	0.03	0.00	0.22	N.D	N.D	0.01	N.D	0.01	0.87

	Spinach (Green Giant)																		
372	Frozen Spinach Cut (Cascadian)	Vegetables	92.09	cluster1	0.20	0.13	0.03	0.02	0.24	0.01	0.04	0.00	0.19	N.D	N.D	0.02	N.D	0.01	0.88
373	Frozen Petite Peas (Birds Eye)	Beans, Peas, Legumes, Nuts, Seeds	90.84	cluster1	4.26	0.23	0.27	0.05	0.50	0.01	0.03	0.00	0.20	N.D	N.D	0.00	N.D	0.03	5.58
374	Frozen Broccoli Florets (Stahlbush)	Vegetables	92.46	cluster1	0.95	0.24	0.14	0.04	0.39	0.02	0.04	0.00	0.28	0.00	N.D	0.03	N.D	0.03	2.17
375	Frozen Spinach Leaf Cut (Signature Select)	Vegetables	92.09	cluster1	0.21	0.13	0.10	0.04	0.12	0.00	0.03	0.00	0.20	N.D	N.D	0.01	N.D	0.01	0.87
376	Frozen Chopped Spinach (Signature Select)	Vegetables	90.84	cluster1	0.29	0.12	0.07	0.04	0.13	0.01	0.03	0.00	0.18	N.D	0.00	0.00	N.D	0.01	0.91
377	Frozen Petite Broccoli Florets (Signature Select)	Vegetables	92.46	cluster1	0.77	0.29	0.11	0.05	0.46	0.02	0.04	0.00	0.33	N.D	N.D	0.01	N.D	0.03	2.13
378	Frozen Petite Brussel Sprouts (Woodstock)	Vegetables	85.69	cluster1	1.99	0.39	0.08	0.05	0.63	0.03	0.06	N.D	0.36	N.D	N.D	0.04	N.D	0.02	3.65
379	Frozen Spinach Cut (Woodstock)	Vegetables	80.28	cluster1	0.97	0.38	0.21	0.09	0.41	0.03	0.07	0.01	0.59	N.D	N.D	0.06	N.D	0.02	2.84
380	Frozen Whole Cauliflower (Stahlbush)	Vegetables	92.89	cluster1	1.23	0.23	0.03	0.05	0.28	0.02	0.03	0.00	0.21	N.D	N.D	0.03	N.D	0.01	2.13
381	Frozen Whole Baby Asparagus	Vegetables	92.15	cluster1	0.58	0.09	0.05	0.07	0.14	0.02	0.03	0.00	0.13	N.D	N.D	0.06	N.D	0.03	1.19

382	Frozen Broccoli Florets (Birds Eye)	Vegetables	91.33	cluster1	0.72	0.23	0.04	0.06	0.34	0.02	0.04	0.00	0.29	N.D	N.D	0.04	N.D	0.03	1.80
383	Frozen Whole Edamame	Vegetables	69.57	cluster1	2.35	0.56	0.08	0.08	0.42	0.05	0.05	0.01	0.17	N.D	N.D	0.18	N.D	0.04	4.00
384	Frozen Petite Brussel Sprouts (Signature Select)	Vegetables	87.67	cluster1	1.90	0.33	0.17	0.07	0.60	0.02	0.05	0.00	0.35	N.D	N.D	0.05	N.D	0.02	3.56
385	Frozen Cauliflower Florets (Signature Select)	Vegetables	93.14	cluster1	1.55	0.26	0.57	0.06	0.32	0.03	0.03	0.00	0.20	N.D	N.D	0.03	N.D	0.01	3.06
386	Frozen French-sliced Green Beans (SIgnature Select)	Vegetables	91.90	cluster1	1.50	0.48	0.16	0.04	0.17	0.02	0.03	0.00	0.36	N.D	N.D	0.02	N.D	0.01	2.80
387	Frozen Broccoli Florets (Cascadian)	Vegetables	90.38	cluster1	0.92	0.26	0.12	0.06	0.42	0.02	0.04	0.00	0.29	N.D	N.D	0.03	N.D	0.02	2.18
388	Frozen Petite Brussel Sprouts (Signature Select)	Vegetables	87.60	cluster1	1.56	0.38	0.06	0.06	0.65	0.03	0.06	0.00	0.37	N.D	N.D	0.05	N.D	0.02	3.25
389	Frozen Cut Green Beans (Cascadian)	Vegetables	91.07	cluster1	1.43	0.42	0.03	0.05	0.16	0.02	0.03	0.00	0.41	N.D	0.00	0.05	N.D	0.01	2.60
390	Cilantro	Vegetables	87.56	cluster1	0.46	0.16	0.70	0.05	0.08	N.D	0.05	0.02	0.36	N.D	N.D	0.03	N.D	0.01	1.91
391	Garlic	Vegetables	56.89	cluster1	0.88	0.40	3.22	0.06	0.11	0.06	0.03	N.D	0.26	N.D	N.D	N.D	N.D	0.01	5.03
392	Onion	Vegetables	91.78	cluster1	1.66	0.20	0.45	0.02	0.02	0.02	0.01	N.D	0.21	N.D	N.D	0.00	N.D	0.00	2.60
393	Jalapeno	Vegetables	92.94	cluster1	1.10	0.14	0.36	0.03	0.06	0.02	0.05	N.D	0.27	N.D	N.D	0.01	N.D	0.01	2.04

394	Parsley	Vegetables	84.66	cluster1	1.01	0.16	0.76	0.05	0.18	N.D	0.03	0.00	0.39	N.D	N.D	0.02	N.D	0.02	2.62
395	Basil	Vegetables	86.93	cluster1	1.24	0.24	0.56	0.05	0.06	N.D	0.03	0.00	0.62	N.D	N.D	0.01	N.D	0.01	2.82
396	Baby Spring Mix	Vegetables	93.15	cluster1	0.27	0.12	0.23	0.04	0.04	0.00	0.02	0.00	0.27	N.D	N.D	0.02	N.D	0.01	1.03
397	Broccoli	Vegetables	86.46	cluster1	1.06	0.34	0.90	0.09	0.35	0.01	0.04	0.00	0.37	N.D	N.D	0.07	N.D	0.03	3.27
398	Pickles	Vegetables	94.98	cluster1	0.41	0.11	0.44	0.03	0.03	0.00	0.01	N.D	0.14	N.D	N.D	0.01	N.D	0.00	1.17
399	Tomato sauce	Fruits	90.32	cluster1	1.58	0.07	0.31	0.02	0.03	0.03	0.01	0.00	0.28	N.D	N.D	0.02	N.D	0.01	2.36
400	Soy sauce	Beans, Peas, Legumes, Nuts, Seeds	68.36	cluster1	0.26	0.52	0.03	0.16	0.15	0.05	0.03	0.00	0.05	0.00	N.D	0.15	N.D	0.01	1.41
401	American Cheese	Milk and Milk Products	20.39	cluster1	1.79	1.63	0.05	0.00	0.01	0.01	0.00	0.00	0.00	N.D	N.D	0.02	N.D	0.01	3.53
402	Sharp Chedder Cheese	Milk and Milk Products	34.41	cluster1	0.02	0.08	0.02	0.00	0.00	0.00	0.00	0.00	0.00	N.D	N.D	0.01	N.D	0.00	0.15
403	Coca cola	Sugars, Sweets, and Beverages	88.83	cluster1	3.76	0.00	1.56	0.00	0.01	0.01	0.00	0.00	0.00	N.D	N.D	0.01	N.D	0.00	5.35
404	Sprite	Sugars, Sweets, and Beverages	90.02	cluster1	3.70	0.00	1.45	0.00	0.01	0.01	0.00	0.00	0.00	N.D	N.D	0.01	N.D	0.00	5.19
405	Pinenuts	Beans, Peas, Legumes, Nuts, Seeds	0.26	cluster1	5.50	0.43	0.86	0.15	0.61	0.04	0.03	0.00	0.06	N.D	N.D	0.04	N.D	0.02	7.73
406	Walnuts	Beans, Peas,	1.84	cluster1	1.69	0.35	0.17	0.30	0.49	0.10	0.07	0.01	0.20	N.D	N.D	0.04	N.D	0.01	3.44

		Legumes, Nuts, Seeds																	
407	Coffee grounds	Sugars, Sweets, and Beverages	0.78	cluster1	0.23	2.39	0.13	0.06	0.61	0.01	0.07	0.03	0.03	N.D	N.D	6.49	N.D	0.02	10.08
408	Protein powder	Milk and Milk Products	4.79	cluster1	1.88	1.93	0.07	0.00	0.02	0.05	0.00	0.00	0.00	0.02	N.D	0.32	N.D	0.01	4.30
409	Black tea	Sugars, Sweets, and Beverages	99.21	cluster1	0.04	0.01	0.01	0.00	0.01	0.00	0.00	0.00	0.00	N.D	N.D	0.00	N.D	0.00	0.07
410	Gatorade	Sugars, Sweets, and Beverages	95.39	cluster1	1.77	0.01	0.17	0.00	0.00	0.02	0.00	0.00	0.00	N.D	N.D	0.00	N.D	0.00	1.98
411	Cranberry juice	Sugars, Sweets, and Beverages	88.71	cluster1	4.10	0.06	0.72	0.00	0.01	0.04	0.00	0.00	0.01	N.D	N.D	0.02	N.D	0.00	4.98
412	Cabernet Sauvignon wine	Sugars, Sweets, and Beverages	97.67	cluster1	0.11	0.02	0.01	0.00	0.02	0.00	0.00	0.00	0.03	N.D	N.D	0.02	N.D	0.00	0.22
413	Budweiser	Sugars, Sweets, and Beverages	96.23	cluster1	1.93	0.02	0.02	0.03	0.03	0.00	0.00	0.00	0.00	N.D	N.D	0.01	N.D	0.00	2.05
414	Sour cream	Milk and Milk Products	73.62	cluster1	1.30	1.22	0.03	0.00	0.00	0.00	0.00	0.00	0.00	0.00	N.D	0.00	N.D	0.01	2.59
415	Yogurt	Milk and Milk Products	83.85	cluster1	1.45	1.61	0.02	0.00	0.00	0.00	0.00	0.00	0.00	N.D	N.D	0.01	N.D	0.01	3.12

416	Unsweetened Vanilla almond milk	Beans, Peas, Legumes, Nuts, Seeds	98.33	cluster1	0.03	0.01	0.01	0.01	0.02	0.00	0.00	0.00	0.00	0.00	N.D	0.00	N.D	0.00	0.08
417	Unsweetened almond milk	Beans, Peas, Legumes, Nuts, Seeds	97.34	cluster1	0.05	0.02	0.01	0.01	0.03	0.00	0.00	0.00	0.00	0.00	N.D	0.00	N.D	0.00	0.12
418	Low sodium chicken broth	Meat, Poultry, Fish, and Mixtures	98.86	cluster1	0.07	0.00	0.06	0.00	0.00	N.D	0.00	0.00	0.00	0.00	N.D	0.00	N.D	0.00	0.14
419	Low sodium vegetable broth	Vegetables	99.60	cluster1	0.06	0.01	0.02	0.00	0.00	0.00	0.00	0.00	0.01	0.00	N.D	0.00	N.D	0.00	0.11
420	Rustic basil pesto	Vegetables	51.57	cluster1	0.21	0.12	0.22	N.D	0.04	N.D	0.01	0.01	0.11	N.D	N.D	N.D	N.D	0.01	0.74
421	Pesto w/ pine nuts and walnuts	Vegetables	33.86	cluster1	0.86	1.68	N.D	0.02	0.01	N.D	0.01	0.01	0.05	0.01	N.D	N.D	N.D	0.01	2.65
422	Pasta sauce w/ tomato and basil	Vegetables	89.57	cluster1	1.33	0.12	0.48	0.04	0.04	N.D	0.02	0.00	0.32	0.00	N.D	0.01	N.D	0.01	2.38
423	Pasta sauce w/ tomato, basil, olive oil	Vegetables	85.68	cluster1	1.72	0.13	0.63	0.05	0.05	N.D	0.02	N.D	0.35	0.00	N.D	0.01	N.D	0.01	2.97
424	Muir's Diced tomatoes	Vegetables	92.97	cluster1	1.25	0.06	0.40	0.02	0.02	N.D	0.01	0.00	0.14	0.00	N.D	0.01	N.D	0.00	1.91
425	Hunt's Tomato paste	Vegetables	69.05	cluster1	4.17	0.27	1.53	0.10	0.08	N.D	0.06	0.00	0.88	0.01	N.D	0.09	N.D	0.02	7.20
426	Traditional italian sauce	Vegetables	83.03	cluster1	3.00	0.13	1.07	0.04	0.05	N.D	0.02	0.00	0.32	N.D	N.D	0.03	N.D	0.01	4.66
427	Hunt's Tomato sauce	Vegetables	86.02	cluster1	1.88	0.11	0.60	0.04	0.03	N.D	0.02	0.00	0.33	0.00	N.D	0.01	N.D	0.01	3.03

428	Muir's Tomato sauce	Vegetables	90.41	cluster1	1.32	0.08	0.44	0.03	0.03	N.D	0.02	0.00	0.27	0.00	N.D	0.00	N.D	0.01	2.20
429	Muir's Tomato paste	Vegetables	71.71	cluster1	4.59	0.20	1.61	0.12	0.09	N.D	0.05	0.00	0.89	N.D	N.D	0.07	N.D	0.03	7.64
430	Chicken Noodle Soup	Meat, Poultry, Fish, and Mixtures	90.65	cluster1	3.82	0.05	0.05	0.05	0.06	0.00	0.00	0.00	0.01	0.00	N.D	0.02	N.D	0.01	4.07
431	Sunflower butter	Beans, Peas, Legumes, Nuts, Seeds	0.01	cluster1	3.63	0.89	0.49	0.32	1.29	0.05	0.09	0.01	0.14	0.01	N.D	0.53	0.00	0.04	7.48
432	Mayonnaise	Fats, Oils, and Salad Dressings	34.64	cluster1	0.08	0.01	0.06	0.01	0.00	0.01	0.00	0.00	0.00	0.00	N.D	0.01	N.D	0.00	0.19
433	Chunky vegetable soup	Vegetables	94.86	cluster1	1.13	0.06	0.10	0.03	0.05	0.00	0.01	0.00	0.10	N.D	N.D	0.00	N.D	0.01	1.50
434	Olive oil	Fats, Oils, and Salad Dressings	0.83	cluster1	0.13	0.01	0.08	0.02	0.00	0.01	0.00	0.00	0.00	0.00	N.D	0.01	N.D	0.00	0.27
435	Unsalted butter	Fats, Oils, and Salad Dressings	18.03	cluster1	0.38	0.32	0.08	0.01	0.00	0.01	0.00	0.00	0.00	0.00	N.D	0.01	N.D	0.00	0.84
436	Pears, blueberries, and spinach blend	Fruits	78.88	cluster1	1.97	0.22	0.69	0.32	0.28	0.02	0.03	0.00	0.27	N.D	N.D	0.03	N.D	0.02	3.86
437	Veggies & wild rice baby food w/ mushroom & parmessan	Vegetables	82.66	cluster1	5.14	0.63	0.00	0.05	0.14	0.01	0.02	N.D	0.12	0.00	N.D	0.02	N.D	0.02	6.14
438	Ataulfo mango	Fruits	61.45	cluster1	6.14	0.33	2.72	0.17	0.41	0.05	0.04	N.D	0.34	N.D	N.D	N.D	N.D	0.01	10.19
439	Bella mango	Fruits	84.67	cluster1	4.50	0.17	1.04	0.06	0.14	0.03	0.02	N.D	0.20	N.D	N.D	N.D	N.D	0.00	6.16
440	Lime	Fruits	90.18	cluster1	0.61	0.08	0.21	0.00	0.02	0.00	0.01	0.00	0.01	N.D	N.D	N.D	N.D	0.00	0.95
441	Kale	Vegetables	85.92	cluster1	0.84	0.34	0.08	0.16	0.34	0.03	0.08	0.00	0.53	0.01	N.D	0.07	N.D	0.03	2.50

442	Red kale	Vegetables	91.32	cluster1	0.30	0.21	N.D	0.11	0.15	0.02	0.05	0.00	0.42	N.D	N.D	0.07	N.D	0.01	1.35
443	Lacinato Kale	Vegetables	89.17	cluster1	0.16	0.24	N.D	0.08	0.13	0.01	0.05	0.00	0.43	0.00	N.D	0.04	N.D	0.02	1.17
444	Green onion	Vegetables	92.30	cluster1	1.55	0.29	0.31	0.09	0.06	0.02	0.02	0.00	0.32	0.00	N.D	0.04	N.D	0.01	2.71
445	Romaine lettuce	Vegetables	94.58	cluster1	0.70	0.11	0.14	0.06	0.08	0.01	0.03	0.00	0.23	0.00	N.D	0.02	N.D	0.01	1.40
446	Eggplant	Vegetables	91.83	cluster1	1.28	0.34	0.14	0.11	0.09	0.01	0.04	0.00	0.36	N.D	N.D	0.05	N.D	0.01	2.41
447	Cabbage	Vegetables	93.14	cluster1	1.52	0.08	0.03	0.07	0.10	0.01	0.02	0.00	0.20	N.D	N.D	0.03	N.D	0.00	2.07
448	Albacore white tuna	Meat, Poultry, Fish, and Mixtures	75.50	cluster1	1.63	0.02	0.03	0.00	0.02	0.00	0.00	0.00	0.01	0.00	N.D	0.01	N.D	0.04	1.76
449	Chunky light tuna	Meat, Poultry, Fish, and Mixtures	74.91	cluster1	1.03	0.02	0.03	0.00	0.01	0.00	0.00	0.00	0.00	0.00	N.D	0.01	N.D	0.07	1.18
450	Chicken breast	Meat, Poultry, Fish, and Mixtures	61.97	cluster1	0.12	0.01	0.03	0.00	0.00	0.00	0.00	0.00	0.00	0.00	N.D	0.03	N.D	0.15	0.36
451	Beef round tip steak	Meat, Poultry, Fish, and Mixtures	59.39	cluster1	0.23	0.02	N.D	0.01	0.00	0.00	0.00	0.00	0.00	0.00	N.D	0.06	N.D	0.08	0.40
452	Shrimp	Meat, Poultry, Fish, and Mixtures	78.74	cluster1	0.47	0.01	0.03	0.00	0.00	0.00	0.00	0.00	0.00	0.00	N.D	0.02	N.D	0.05	0.59
453	Brown egg yolk	Eggs	52.70	cluster1	0.36	0.11	0.04	0.01	0.00	0.01	0.00	0.00	0.00	0.02	0.01	0.50	N.D	0.00	1.06
454	Brown egg white	Eggs	87.69	cluster1	0.33	0.04	0.02	0.00	0.00	0.00	0.00	0.00	0.00	0.02	0.02	0.36	N.D	0.00	0.80
455	Whole brown egg	Eggs	74.32	cluster1	0.47	0.07	0.03	0.00	0.00	0.00	0.00	0.00	0.00	0.04	0.03	0.52	N.D	0.00	1.17

456	Cabbage	Vegetables	94.55	cluster1	1.37	0.12	0.08	0.06	0.12	0.01	0.02	N.D	0.20	0.00	N.D	0.03	N.D	0.00	2.02
457	Whole brown egg	Eggs	77.37	cluster1	0.49	0.07	0.03	0.01	0.00	0.00	0.00	0.00	0.00	0.03	0.03	0.48	0.00	0.00	1.14
458	Thin almond nut crackers	Grain Products	96.09	cluster1	0.01	0.00	0.00	0.00	0.00	0.00	0.00	0.00	0.00	0.00	N.D	0.00	N.D	0.00	0.02
459	Purple cabbage	Vegetables	94.18	cluster1	1.56	0.11	0.23	0.06	0.17	0.01	0.03	N.D	0.24	0.00	N.D	0.02	N.D	0.00	2.43
460	Purple cabbage	Vegetables	91.08	cluster1	1.16	0.12	0.33	0.09	0.21	0.02	0.03	0.00	0.26	0.00	N.D	0.03	N.D	0.01	2.26
461	Coconut flour	Beans, Peas, Legumes, Nuts, Seeds	2.33	cluster5	5.37	5.19	N.D	1.68	1.40	N.D	0.12	0.05	0.32	N.D	N.D	43.09	0.02	N.D	57.24
462	Plantain banana (steamed 10 min)	Fruits	67.29	cluster1	25.50	0.19	0.27	0.05	0.11	0.03	0.00	N.D	0.29	N.D	N.D	N.D	N.D	0.00	26.46
463	Steamed whole boomer gold potato	Vegetables	77.55	cluster1	19.48	0.71	N.D	0.08	0.12	0.01	0.02	N.D	0.11	N.D	N.D	0.03	N.D	0.01	20.56
464	Steamed boomer gold potato flesh only	Vegetables	76.95	cluster1	20.57	0.68	N.D	0.09	0.12	0.01	0.02	N.D	0.13	N.D	N.D	0.07	N.D	0.01	21.70
465	Steamed purple mini potato medley flesh only	Vegetables	78.36	cluster1	16.05	0.55	N.D	0.02	0.07	0.01	0.03	N.D	0.14	N.D	N.D	N.D	N.D	0.00	16.86
466	Steamed red mini potato medley flesh only	Vegetables	77.79	cluster1	16.64	0.38	N.D	0.03	0.09	0.01	0.02	N.D	0.13	N.D	N.D	0.01	N.D	0.01	17.30
467	Steamed whole russet potato	Vegetables	77.26	cluster1	15.91	0.59	N.D	0.03	0.12	0.02	0.02	N.D	0.15	N.D	N.D	N.D	N.D	0.01	16.86

468	Steamed white sweet potato flesh only	Vegetables	76.63	cluster1	24.65	0.58	0.44	0.17	0.28	0.00	0.05	N.D	0.42	N.D	N.D	N.D	N.D	0.01	26.60
469	Sugar free Strawberry jam (Smuckers)	Fruits	72.76	cluster1	19.41	0.34	N.D	0.02	0.07	N.D	0.02	N.D	0.31	N.D	N.D	0.24	N.D	0.00	20.41
470	Classic hummus	Beans, Peas, Legumes, Nuts, Seeds	56.37	cluster1	20.15	1.30	N.D	0.23	0.92	0.04	0.07	N.D	0.24	N.D	N.D	1.08	0.03	N.D	24.07
471	Xanthan gum	Grain Products	7.66	cluster1	19.24	0.10	0.26	0.06	0.14	0.05	0.21	1.49	0.10	N.D	N.D	13.39	N.D	0.02	35.05
472	Oatmeal & Sprouted quinoa w/ banana and strawberries	Grain Products	63.06	cluster1	16.22	0.42	2.06	0.18	0.26	N.D	0.02	N.D	0.21	N.D	N.D	0.27	N.D	N.D	19.63
473	Oatmeal & Sprouted quinoa w/ apple and blueberry	Grain Products	67.35	cluster1	16.76	0.61	1.33	0.36	0.36	N.D	0.02	N.D	0.18	N.D	N.D	0.29	N.D	N.D	19.90
474	Sqash and pumpkin ravioli w/ sage sauce	Grain Products	71.26	cluster1	18.06	0.53	0.16	0.40	0.33	N.D	0.01	N.D	0.19	N.D	N.D	0.09	N.D	N.D	19.77
475	Spinach and Cheese ravioli w/ Marinara sauce	Grain Products	63.94	cluster1	20.39	0.60	0.47	0.91	0.77	N.D	0.03	N.D	0.32	N.D	N.D	0.07	N.D	N.D	23.55
476	Quinoa	Grain Products	67.07	cluster1	19.48	0.32	N.D	0.07	0.44	N.D	0.03	N.D	0.23	N.D	N.D	0.07	N.D	N.D	20.65
477	White rice (Organics)	Grain Products	64.69	cluster1	26.63	0.07	0.18	0.03	0.09	0.01	0.00	0.00	0.00	N.D	N.D	0.01	N.D	0.01	27.04
478	Enriched long white rice (Mahatma)	Grain Products	71.30	cluster1	26.66	0.13	N.D	0.05	0.07	N.D	0.00	N.D	0.00	N.D	N.D	0.04	N.D	N.D	26.95

479	Brown rice (Mahatma)	Grain Products	73.05	cluster1	21.06	0.10	0.12	0.08	0.17	0.01	0.00	0.00	0.00	N.D	N.D	0.01	N.D	0.01	21.58
480	Wheat farina cereal/cream of wheat	Grain Products	83.86	cluster1	14.58	0.13	N.D	0.17	0.14	N.D	0.00	N.D	0.00	N.D	N.D	0.02	N.D	N.D	15.05
481	Rolled oats	Grain Products	75.70	cluster1	19.46	0.27	N.D	0.48	0.41	N.D	0.00	N.D	0.01	N.D	N.D	0.04	N.D	N.D	20.67
482	Organic bulgur and quinoa mix	Grain Products	64.36	cluster1	20.74	0.29	N.D	0.34	0.32	N.D	0.01	N.D	0.06	N.D	N.D	0.16	N.D	N.D	21.91
483	Gluten-free spaghetti	Grain Products	71.93	cluster1	23.19	0.07	0.13	0.05	0.21	0.01	0.00	0.00	0.00	N.D	N.D	0.01	N.D	0.01	23.67
484	Spaghetti made w/ zucchini and spinach puree	Grain Products	60.20	cluster1	26.37	0.11	0.18	0.27	0.47	0.01	0.00	0.00	0.01	N.D	N.D	0.03	N.D	0.02	27.47
485	No. 5 Spaghetti	Grain Products	59.65	cluster1	24.03	0.14	0.15	0.35	0.71	0.01	0.00	0.00	0.00	N.D	N.D	0.03	N.D	0.02	25.43
486	Chickpea spaghetti	Beans, Peas, Legumes, Nuts, Seeds	63.12	cluster1	16.83	0.21	N.D	0.61	0.45	N.D	0.01	N.D	0.02	N.D	N.D	N.D	N.D	N.D	18.12
487	Black bean penne	Beans, Peas, Legumes, Nuts, Seeds	59.71	cluster1	24.80	0.89	N.D	0.34	1.63	N.D	0.05	0.02	0.26	0.03	0.01	0.27	N.D	0.03	28.32
488	Green lentil fusili	Beans, Peas, Legumes, Nuts, Seeds	59.82	cluster1	24.17	0.99	N.D	0.09	0.74	N.D	0.04	N.D	0.15	N.D	N.D	0.01	N.D	0.04	26.22
489	Inca red quinoa	Grain Products	55.75	cluster1	20.00	0.33	0.05	0.09	0.73	N.D	0.05	N.D	0.57	N.D	N.D	N.D	N.D	N.D	21.82
490	Red whole grain quinoa	Grain Products	61.62	cluster1	17.10	0.20	N.D	0.10	0.84	N.D	0.09	N.D	0.71	0.02	0.01	0.03	N.D	0.10	19.19

491	Whole grain quinoa	Grain Products	66.66	cluster1	25.57	N.D	0.05	0.07	0.54	N.D	0.07	N.D	0.31	N.D	N.D	N.D	N.D	0.07	26.68
492	Red whole grain bulgur	Grain Products	72.64	cluster1	24.56	0.24	N.D	0.56	0.48	N.D	0.00	0.00	0.01	N.D	N.D	0.04	N.D	0.01	25.92
493	Tri-color whole grain quinoa	Grain Products	69.32	cluster1	15.62	0.11	0.10	0.03	0.61	0.03	0.02	0.01	0.05	N.D	N.D	0.02	N.D	0.03	16.64
494	Whole grain sorghum	Grain Products	65.42	cluster1	22.17	0.14	0.11	0.15	0.34	0.02	0.00	0.01	0.01	N.D	N.D	0.02	N.D	0.01	22.97
495	Whole grain kasha	Grain Products	65.15	cluster1	21.66	0.13	0.10	0.04	0.40	0.02	0.02	0.00	0.03	N.D	N.D	0.01	N.D	0.02	22.44
496	Whole grain brown lentils	Beans, Peas, Legumes, Nuts, Seeds	75.18	cluster1	17.22	0.89	N.D	0.08	0.67	N.D	0.04	N.D	0.14	N.D	N.D	0.07	N.D	0.03	19.14
497	Whole grain red lentils	Beans, Peas, Legumes, Nuts, Seeds	67.62	cluster1	17.76	1.00	N.D	0.07	0.69	N.D	0.04	N.D	0.11	N.D	N.D	0.04	N.D	0.04	19.75
498	Dried corn grits/polenta	Grain Products	71.27	cluster1	21.37	0.05	0.15	0.05	0.21	0.01	0.00	0.00	0.00	N.D	N.D	0.01	N.D	0.01	21.86
499	Whole grain rolled oats	Grain Products	72.11	cluster1	17.26	0.09	0.11	0.26	0.32	0.02	0.00	0.00	0.00	N.D	N.D	0.03	N.D	0.01	18.10
500	Whole grain millets	Grain Products	62.38	cluster1	16.04	0.11	0.10	0.06	0.24	0.02	0.00	0.00	0.00	N.D	N.D	0.01	N.D	0.01	16.61
501	Whole grain farro	Grain Products	69.54	cluster1	17.97	0.21	0.13	0.43	0.73	0.01	0.00	0.00	0.00	N.D	N.D	0.04	N.D	0.01	19.53
502	Whole grain pearl barley	Grain Products	72.68	cluster1	16.77	0.06	0.11	0.32	0.70	0.02	0.00	0.00	0.00	N.D	N.D	0.04	N.D	0.01	18.04
503	Whole grain irish oat	Grain Products	68.08	cluster1	22.68	0.27	N.D	0.35	0.32	N.D	0.01	0.01	0.05	0.02	0.01	0.09	N.D	0.03	23.83
504	Freeze dried Roasted red pepper	Vegetables	3.55	cluster1	22.54	0.64	5.00	0.39	0.53	N.D	0.37	N.D	3.09	N.D	N.D	0.13	N.D	0.05	32.74
505	Carrot sticks	Vegetables	0.43	cluster1	24.11	5.61	0.39	0.20	2.36	N.D	0.61	N.D	5.06	N.D	N.D	0.53	N.D	0.09	38.96

506	Dried apricots	Fruits	9.92	cluster1	18.80	1.02	2.12	0.67	1.44	N.D	0.23	N.D	1.68	N.D	N.D	0.06	N.D	0.04	26.08
507	Freeze dried raspberry	Fruits	0.56	cluster1	17.29	1.23	4.13	3.31	1.61	N.D	0.30	N.D	1.54	N.D	N.D	0.19	N.D	0.05	29.66
508	Freeze dried apple rings	Fruits	9.63	cluster1	25.78	2.16	3.63	0.85	2.22	N.D	0.19	N.D	1.62	N.D	N.D	0.30	N.D	0.02	36.77
509	Flaxseed crackers	Beans, Peas, Legumes, Nuts, Seeds	1.54	cluster1	20.17	1.06	0.46	0.70	2.18	0.18	0.15	0.01	0.23	N.D	N.D	0.40	N.D	0.06	25.60
510	Flax bread (Food for life)	Grain Products	41.65	cluster1	22.24	N.D	0.30	0.51	1.16	0.05	0.02	0.00	0.02	N.D	N.D	0.07	N.D	0.03	24.42
511	Frozen falafel	Beans, Peas, Legumes, Nuts, Seeds	46.67	cluster1	20.91	2.51	0.35	0.22	2.00	N.D	0.12	N.D	0.45	N.D	N.D	0.09	N.D	0.05	26.71
512	Kale and quinoa burger patty	Vegetable s	49.80	cluster1	17.34	0.37	0.11	0.14	0.24	N.D	0.02	N.D	0.22	N.D	N.D	N.D	N.D	0.02	18.46
513	Pumpkin and spinach burger patty	Grain Products	47.08	cluster1	15.69	0.56	0.13	0.16	0.25	N.D	0.03	N.D	0.31	N.D	N.D	N.D	N.D	0.02	17.15
514	Barley and quinoa burger patty	Beans, Peas, Legumes, Nuts, Seeds	31.48	cluster1	18.61	0.70	1.36	0.41	0.78	N.D	0.05	N.D	0.34	N.D	N.D	N.D	N.D	0.06	22.31
515	Pumpkin Seed & Flaxseed Granola	Grain Products	3.07	cluster1	23.11	0.34	0.40	0.18	0.29	0.02	0.01	0.00	0.00	N.D	N.D	0.02	N.D	0.01	24.38
516	Original Waffle	Grain Products	41.07	cluster1	21.80	0.25	0.21	0.26	0.33	0.03	0.00	0.00	0.00	N.D	N.D	0.04	N.D	0.01	22.95
517	8 Whole Grain, Multigrain Waffle	Grain Products	47.38	cluster1	22.66	0.37	0.15	0.71	0.60	0.04	0.01	0.00	0.00	N.D	N.D	0.04	N.D	0.01	24.59

518	Blueberry Waffle	Grain Products	39.87	cluster1	19.41	0.15	0.35	0.76	0.54	0.01	0.00	0.00	0.00	N.D	N.D	0.06	N.D	0.01	21.29
519	Original Pancake	Grain Products	51.54	cluster1	24.35	0.26	0.19	0.21	0.29	0.01	0.00	0.00	0.00	N.D	N.D	0.06	N.D	0.01	25.38
520	Frozen Golden Corn Kernels (Siganture Select)	Vegetable s	76.00	cluster1	17.63	0.19	1.67	0.35	0.65	0.00	0.01	0.00	0.02	N.D	N.D	0.07	N.D	0.02	20.61
521	Cranberry	Fruits	6.02	cluster1	26.54	0.34	4.89	0.19	0.35	0.33	0.02	N.D	0.32	N.D	N.D	0.02	N.D	0.00	32.99
522	Chocolate	Sugars, Sweets, and Beverages	0.11	cluster1	23.05	2.56	2.26	0.05	0.19	0.17	0.02	0.00	0.07	N.D	N.D	0.05	N.D	0.01	28.43
523	Dates	Fruits	8.85	cluster1	24.52	0.34	6.97	0.21	0.25	0.25	0.04	N.D	0.46	N.D	N.D	0.03	N.D	0.01	33.09
524	Vegan white cheddar chickepea puffs	Beans, Peas, Legumes, Nuts, Seeds	49.03	cluster1	24.55	0.86	0.22	0.11	1.43	0.01	0.04	0.00	0.06	0.00	N.D	0.03	N.D	0.02	27.35
525	Bananas and carrots oat bar	Grain Products	11.42	cluster1	24.74	0.59	4.34	0.24	0.18	0.05	0.05	N.D	0.23	0.00	N.D	0.02	N.D	0.02	30.47
526	Apples and spinach oat bar	Grain Products	8.81	cluster1	27.35	0.73	3.88	0.45	0.36	0.04	0.06	N.D	0.22	0.04	N.D	0.13	N.D	0.02	33.28
527	Powerseed bread sweetened w/ fruit juice	Grain Products	38.01	cluster1	22.26	0.34	0.27	1.48	1.07	0.01	0.02	0.01	0.05	0.00	N.D	0.12	N.D	0.02	25.67
528	Almond flour	Beans, Peas, Legumes, Nuts, Seeds	4.53	cluster1	26.59	0.42	0.36	0.21	0.95	0.03	0.05	0.01	0.06	0.00	N.D	0.18	N.D	0.01	28.88
529	Bean threads (vermicelli)	Beans, Peas, Legumes, Nuts, Seeds	79.81	cluster1	18.25	0.08	0.11	0.00	0.04	0.00	0.00	0.00	0.00	0.00	N.D	0.00	N.D	0.00	18.49

530	Chickpeas rotini pasta	Beans, Peas, Legumes, Nuts, Seeds	61.07	cluster1	17.14	0.93	0.19	0.10	1.06	0.02	0.03	0.02	0.04	0.00	N.D	0.11	N.D	0.03	19.67
531	Yellow lentil penne	Beans, Peas, Legumes, Nuts, Seeds	60.79	cluster1	22.37	1.01	0.21	0.09	0.88	0.01	0.03	0.01	0.03	0.00	N.D	0.04	N.D	0.04	24.73
532	Toasted Pearl couscous	Grain Products	70.16	cluster1	21.99	0.18	0.18	0.23	0.25	0.00	0.00	0.00	0.00	0.00	N.D	0.05	N.D	0.01	22.89
533	Mix couscous	Grain Products	78.83	cluster1	14.87	0.11	0.13	0.15	0.16	0.00	0.00	0.00	0.00	0.00	N.D	0.03	N.D	0.01	15.47
534	Brown long grain rice	Grain Products	69.29	cluster1	25.25	0.14	0.17	0.08	0.15	0.00	0.00	0.00	0.00	0.00	N.D	0.02	N.D	0.01	25.83
535	Wild blend rice	Grain Products	74.28	cluster1	18.11	0.16	0.14	0.08	0.13	0.00	0.00	0.00	0.01	0.00	N.D	0.02	N.D	0.01	18.67
536	Tri-color couscous	Grain Products	75.15	cluster1	19.99	0.20	0.15	0.26	0.27	0.00	0.00	0.00	0.00	0.00	N.D	0.04	N.D	0.00	20.92
537	Spinach and ricotta ravioli	Grain Products	51.91	cluster1	20.61	1.51	N.D	0.34	0.22	0.01	0.01	N.D	0.03	N.D	N.D	0.06	N.D	0.01	22.79
538	Cheddar cheese, beans, and rice burrito	Beans, Peas, Legumes, Nuts, Seeds	51.77	cluster1	24.24	0.37	0.25	0.55	0.90	0.03	0.02	0.01	0.04	0.00	N.D	0.10	N.D	0.02	26.53
539	Red lentil pasta	Beans, Peas, Legumes, Nuts, Seeds	59.84	cluster1	19.36	0.66	0.22	0.08	0.86	0.01	0.02	0.01	0.02	0.00	0.00	0.04	N.D	0.04	21.33
540	Yellow banana flesh only	Fruits	75.94	cluster1	14.14	0.12	1.83	0.04	0.07	0.01	0.00	N.D	0.25	N.D	N.D	0.08	N.D	N.D	16.55
541	Red banana (steamed 10min)	Fruits	73.67	cluster1	13.51	0.16	0.83	0.10	0.12	0.01	0.01	N.D	0.29	N.D	N.D	0.12	N.D	N.D	15.15

542	Whole yellow nectarine (HGO Farms)	Fruits	83.89	cluster1	6.46	0.18	1.77	0.07	0.34	0.02	0.02	N.D	0.40	N.D	N.D	0.03	N.D	0.00	9.29
543	Yellow mango	Fruits	76.53	cluster1	11.19	0.97	3.94	0.27	0.67	0.11	0.04	N.D	0.49	N.D	N.D	0.10	N.D	N.D	17.79
544	Pixie tangerine	Fruits	77.95	cluster1	7.57	0.26	2.26	0.06	0.18	0.02	0.09	N.D	0.34	N.D	N.D	0.03	N.D	N.D	10.81
545	Golden kiwi	Fruits	76.39	cluster1	9.22	0.17	4.06	0.03	0.06	0.02	0.01	N.D	0.22	N.D	N.D	0.04	N.D	N.D	13.83
546	Green seedless grape	Fruits	81.37	cluster1	7.66	0.09	2.65	0.02	0.05	0.01	0.01	N.D	0.11	N.D	N.D	0.00	N.D	N.D	10.59
547	Steamed swet white corn kernels	Vegetables	77.52	cluster1	8.98	0.29	0.13	0.71	0.56	0.02	0.01	N.D	0.03	N.D	N.D	0.04	N.D	N.D	10.76
548	Steamed whole blushing belle potato	Vegetables	84.12	cluster1	8.60	0.37	0.07	0.05	0.09	0.00	0.02	N.D	0.13	N.D	N.D	N.D	N.D	0.01	9.35
549	Steamed blushing belle potato flesh only	Vegetables	81.65	cluster1	13.43	0.62	0.18	0.06	0.11	0.00	0.02	N.D	0.15	N.D	N.D	0.01	N.D	0.01	14.57
550	Pureed sweet potato flesh only	Vegetables	81.08	cluster1	6.41	0.34	0.13	0.04	0.16	0.02	0.04	N.D	0.41	N.D	N.D	0.00	N.D	0.01	7.56
551	Steamed whole purple mini potato medley	Vegetables	80.10	cluster1	14.10	0.48	N.D	0.01	0.08	0.03	0.03	N.D	0.14	N.D	N.D	N.D	N.D	0.01	14.87
552	Steamed whole red mini potato medley	Vegetables	80.63	cluster1	11.25	0.36	0.08	0.04	0.08	0.00	0.02	N.D	0.13	N.D	N.D	N.D	N.D	0.01	11.97
553	Steamed whole yellow mini potato medley	Vegetables	85.56	cluster1	7.51	0.44	N.D	0.02	0.07	0.01	0.01	N.D	0.14	N.D	N.D	0.00	N.D	0.01	8.20
554	Steamed yellow mini potato medley flesh only	Vegetables	82.51	cluster1	10.29	0.38	N.D	0.01	0.07	0.02	0.01	N.D	0.11	N.D	N.D	0.00	N.D	0.01	10.91
555	Steamed russet potato flesh only	Vegetables	76.63	cluster1	13.45	0.55	N.D	0.05	0.08	0.02	0.01	N.D	0.08	N.D	N.D	N.D	N.D	0.00	14.25

556	Steamed whole garnet yam	Vegetables	79.52	cluster1	7.72	0.35	N.D	0.11	0.18	0.03	0.04	N.D	0.28	N.D	N.D	0.00	N.D	0.01	8.72
557	Steamed garnet yam flesh only	Vegetables	80.22	cluster1	7.53	0.41	N.D	0.05	0.18	0.05	0.03	N.D	0.26	N.D	N.D	N.D	N.D	0.01	8.51
558	Pureed garnet yam flesh only	Vegetables	81.35	cluster1	8.24	0.47	1.51	0.13	0.21	0.01	0.03	N.D	0.29	N.D	N.D	N.D	N.D	0.01	10.90
559	Steamed whole red sweet potato	Vegetables	81.42	cluster1	9.00	0.44	0.15	0.26	0.23	0.02	0.05	N.D	0.42	N.D	N.D	0.00	N.D	0.01	10.59
560	Steamed red sweet potato flesh only	Vegetables	79.66	cluster1	8.38	0.37	N.D	0.16	0.20	0.03	0.05	N.D	0.43	N.D	N.D	0.01	N.D	0.01	9.65
561	Steamed golden sweet potato flesh only	Vegetables	79.19	cluster1	10.39	0.34	N.D	0.13	0.18	0.02	0.04	N.D	0.34	N.D	N.D	0.01	N.D	0.01	11.46
562	Steamed whole oyster mushrooms	Vegetables	87.27	cluster1	7.32	0.19	N.D	0.15	0.02	0.06	0.00	0.04	0.00	N.D	N.D	0.25	N.D	0.03	8.05
563	Cashew butter (Open Nature)	Beans, Peas, Legumes, Nuts, Seeds	0.85	cluster1	14.71	1.21	N.D	0.24	0.49	0.04	0.04	N.D	0.34	N.D	N.D	N.D	0.04	N.D	17.10
564	Peanut Butter (Justin's)	Beans, Peas, Legumes, Nuts, Seeds	0.00	cluster1	10.23	1.12	N.D	0.93	2.58	0.13	0.14	N.D	0.51	N.D	N.D	0.15	0.03	N.D	15.82
565	Hazelnut and Almond Butter (Justin's)	Beans, Peas, Legumes, Nuts, Seeds	10.02	cluster1	7.52	0.91	N.D	0.69	1.97	0.13	0.14	N.D	0.51	N.D	N.D	N.D	0.01	N.D	11.87
566	Vanilla almond Butter (Justin's)	Beans, Peas,	0.66	cluster1	10.09	1.63	0.09	0.98	3.79	0.14	0.20	N.D	0.37	N.D	N.D	N.D	0.02	N.D	17.32

		Legumes, Nuts, Seeds																	
567	Rice milk	Grain Products	89.10	cluster1	8.93	N.D	N.D	N.D	0.01	N.D	0.00	N.D	0.00	N.D	N.D	0.02	N.D	N.D	8.96
568	Roasted red pepper hummus	Beans, Peas, Legumes, Nuts, Seeds	42.15	cluster1	8.75	0.87	0.17	0.13	0.83	0.04	0.05	0.01	0.11	N.D	N.D	0.52	N.D	0.03	11.50
569	Roasted pine nut hummus	Beans, Peas, Legumes, Nuts, Seeds	56.72	cluster1	8.89	1.12	N.D	0.11	0.91	N.D	0.07	N.D	0.25	N.D	N.D	0.22	N.D	0.04	11.60
570	Canned low sodium pinto bean	Beans, Peas, Legumes, Nuts, Seeds	75.80	cluster1	8.62	0.16	0.09	0.16	0.77	0.03	0.01	0.01	0.03	N.D	N.D	0.08	N.D	0.01	9.98
571	Canned black beans (S&W)	Beans, Peas, Legumes, Nuts, Seeds	76.95	cluster1	8.78	0.23	0.08	0.18	1.56	0.04	0.01	0.01	0.04	N.D	N.D	0.11	N.D	0.02	11.06
572	Canned garbanzo beans	Beans, Peas, Legumes, Nuts, Seeds	74.67	cluster1	9.91	0.31	0.07	0.06	1.28	0.02	0.02	0.00	0.03	N.D	N.D	0.02	N.D	0.02	11.74
573	Canned black beans (Organics)	Beans, Peas, Legumes, Nuts, Seeds	75.25	cluster1	7.14	0.18	0.06	0.17	1.34	0.04	0.01	0.01	0.04	N.D	N.D	0.09	N.D	0.02	9.10
574	Canned Kidney beans	Beans, Peas,	67.57	cluster1	10.61	0.61	0.13	0.31	0.99	0.09	0.03	0.02	0.13	N.D	N.D	0.21	N.D	0.02	13.15

		Legumes, Nuts, Seeds																	
575	Canned Blackeye beans	Beans, Peas, Legumes, Nuts, Seeds	73.90	cluster1	11.16	0.18	0.07	0.07	0.89	0.03	0.02	0.01	0.04	N.D	N.D	0.03	N.D	0.02	12.51
576	Canned Butter beans	Beans, Peas, Legumes, Nuts, Seeds	72.31	cluster1	8.02	0.18	0.06	0.19	1.27	0.03	0.02	0.01	0.06	N.D	N.D	0.35	N.D	0.01	10.19
577	Canned garbanzo beans (S&W)	Beans, Peas, Legumes, Nuts, Seeds	66.89	cluster1	14.01	0.29	0.08	0.07	1.69	0.05	0.03	0.01	0.05	N.D	N.D	0.03	N.D	0.02	16.31
578	Commercial Mac and Cheese	Vegetables	74.01	cluster1	13.15	0.52	0.53	0.02	0.23	N.D	0.02	N.D	0.16	N.D	N.D	0.06	N.D	N.D	14.69
579	Organic Happy Baby 293	Fruits	78.23	cluster1	8.71	0.21	2.17	0.09	0.11	N.D	0.02	N.D	0.36	N.D	N.D	0.04	N.D	0.01	11.72
580	Organic Happy Baby 298	Fruits	73.22	cluster1	7.60	0.14	0.98	0.08	0.10	0.05	0.01	0.00	0.15	N.D	N.D	0.09	N.D	0.00	9.20
581	Organic Happy Baby 301	Fruits	83.24	cluster1	8.21	0.20	3.52	0.15	0.16	N.D	0.01	N.D	0.13	N.D	N.D	0.01	0.01	N.D	12.39
582	Organic Happy Baby 302	Fruits	77.47	cluster1	11.03	0.06	0.14	0.11	0.16	N.D	0.01	N.D	0.18	N.D	N.D	0.08	N.D	0.01	11.77
583	Organic Happy Baby 304	Fruits	78.15	cluster1	8.72	0.19	2.07	0.07	0.10	N.D	0.01	N.D	0.16	N.D	N.D	0.07	N.D	N.D	11.38
584	Beach-Nut Naturals 309	Fruits	79.03	cluster2	6.89	0.43	0.89	0.13	0.26	0.06	0.04	N.D	0.47	N.D	N.D	0.05	N.D	0.01	9.23
585	Beach-Nut Naturals 310	Vegetables	81.96	cluster2	6.40	0.16	0.18	0.08	0.11	N.D	0.03	N.D	0.21	N.D	N.D	N.D	N.D	0.01	7.17
586	Beach-Nut Naturals 312	Fruits	75.33	cluster2	10.96	0.10	3.57	0.05	0.08	N.D	0.01	N.D	0.27	N.D	N.D	N.D	N.D	0.01	15.05

587	Beach-Nut Naturals 316	Fruits	76.70	cluster2	8.60	0.26	4.11	0.19	0.23	0.02	0.02	N.D	0.28	N.D	N.D	0.04	0.01	N.D	13.74
588	Beach-Nut Naturals 317	Fruits	79.40	cluster2	6.74	0.18	2.46	0.03	0.21	N.D	0.02	N.D	0.21	N.D	N.D	0.01	N.D	N.D	9.87
589	Beach-Nut Naturals 322	Fruits	78.69	cluster2	7.33	0.23	3.28	0.43	0.15	0.01	0.01	N.D	0.24	N.D	N.D	0.07	0.01	N.D	11.76
590	Plum Organics 337	Vegetables	87.84	cluster2	6.86	0.29	N.D	0.10	0.14	N.D	0.03	N.D	0.20	N.D	N.D	N.D	0.01	N.D	7.63
591	Plum Organics 338	Fruits	77.71	cluster2	8.02	0.20	2.26	0.14	0.20	N.D	0.02	N.D	0.20	N.D	N.D	0.01	N.D	N.D	11.06
592	Plum Organics 339	Fruits	82.85	cluster2	8.80	0.19	0.34	0.09	0.22	N.D	0.03	N.D	0.30	N.D	N.D	0.02	N.D	0.01	10.01
593	Plum Organics 341	Fruits	83.69	cluster2	10.58	0.47	3.18	0.31	0.38	0.05	0.04	N.D	0.44	N.D	N.D	N.D	0.02	N.D	15.48
594	Plum Organics 345	Fruits	79.26	cluster2	7.73	0.26	2.85	0.09	0.16	0.01	0.01	N.D	0.20	N.D	N.D	0.10	0.02	N.D	11.43
595	Plum Organics 347	Fruits	82.01	cluster2	7.43	0.47	3.61	0.02	0.08	0.00	0.01	N.D	0.18	N.D	N.D	0.05	0.01	N.D	11.85
596	Plum Organics 348	Fruits	82.94	cluster2	6.78	0.45	2.93	0.41	0.28	0.01	0.01	N.D	0.15	N.D	N.D	0.07	0.01	N.D	11.11
597	Organics 364	Fruits	78.36	cluster2	6.62	0.24	1.93	0.50	0.40	N.D	0.01	N.D	0.25	N.D	N.D	0.11	N.D	N.D	10.05
598	Organics 366	Fruits	77.33	cluster2	9.45	0.15	1.68	0.21	0.14	N.D	0.01	N.D	0.27	0.01	0.00	0.10	N.D	0.01	12.02
599	Pumpkin Tree 372	Fruits	75.13	cluster2	10.63	0.08	2.81	0.09	0.07	N.D	0.01	N.D	0.28	N.D	N.D	0.13	N.D	0.01	14.10
600	Gerber Natural 379	Fruits	75.41	cluster2	10.49	0.19	2.50	0.11	0.12	N.D	0.01	N.D	0.25	N.D	N.D	0.12	N.D	N.D	13.79
601	Gerber Natural 381	Fruits	76.09	cluster2	9.86	0.14	1.16	0.08	0.10	N.D	0.01	N.D	0.25	N.D	N.D	0.07	N.D	0.00	11.66
602	Geber Organics 389	Vegetables	82.50	cluster2	7.12	0.14	2.32	0.04	0.07	N.D	0.02	N.D	0.32	0.01	0.00	0.06	N.D	0.01	10.13
603	Geber Organics 399	Fruits	67.37	cluster2	9.24	0.13	2.22	0.07	0.12	0.11	0.01	0.00	0.29	N.D	N.D	0.13	N.D	0.01	12.33
604	Geber Organics 400	Fruits	78.83	cluster2	7.79	0.12	1.94	0.28	0.23	0.01	0.01	N.D	0.16	N.D	N.D	N.D	0.01	N.D	10.55
605	Gerber 404	Fruits	73.19	cluster2	6.47	0.28	2.09	0.56	0.21	N.D	0.02	N.D	0.36	N.D	N.D	0.07	N.D	N.D	10.05
606	Gerber 405	Fruits	77.09	cluster2	10.80	0.08	2.06	0.05	0.07	N.D	0.01	N.D	0.28	N.D	N.D	0.13	N.D	0.01	13.48
607	Gerber 406	Fruits	79.85	cluster2	6.65	1.08	2.01	0.22	0.12	N.D	0.01	N.D	0.26	N.D	N.D	0.03	N.D	N.D	10.39

608	Gerber 410	Fruits	82.53	cluster2	7.96	0.07	1.45	0.07	0.05	N.D	0.01	N.D	0.19	N.D	N.D	0.07	N.D	0.00	9.87
609	Gerber 411	Vegetables	82.97	cluster2	7.34	0.29	2.71	0.05	0.21	N.D	0.04	N.D	0.39	0.01	0.00	0.07	N.D	0.01	11.13
610	Gerber 415	Fruits	67.87	cluster2	11.36	0.61	1.28	1.14	0.91	N.D	0.05	N.D	0.45	N.D	N.D	0.06	N.D	0.02	15.89
611	Gerber 416	Fruits	79.83	cluster2	9.68	0.29	1.26	0.44	0.33	N.D	0.01	N.D	0.17	N.D	N.D	0.12	N.D	0.01	12.31
612	Gerber 417	Fruits	82.62	cluster2	6.34	0.21	1.56	0.28	0.18	N.D	0.02	N.D	0.26	N.D	N.D	0.05	N.D	0.01	8.90
613	Red lentil fusili	Beans, Peas, Legumes, Nuts, Seeds	59.82	cluster2	13.73	0.59	N.D	0.06	0.52	N.D	0.03	N.D	0.09	0.02	0.01	N.D	N.D	0.04	15.08
614	Scottish oatmeal	Grain Products	77.99	cluster2	12.46	0.07	0.07	0.19	0.39	0.01	0.00	0.00	0.00	N.D	N.D	0.04	N.D	0.01	13.25
615	Hot cereal oat bran	Grain Products	82.84	cluster2	7.36	0.07	0.05	0.12	0.33	0.01	0.00	0.00	0.00	N.D	N.D	0.02	N.D	0.01	7.97
616	Hot cereal whole grain creamy buckwheat	Grain Products	81.18	cluster2	11.23	0.16	0.07	0.03	0.23	0.01	0.01	0.00	0.01	N.D	N.D	0.01	N.D	0.01	11.77
617	Steel cut whole grain oats	Grain Products	75.37	cluster2	12.71	0.08	0.10	0.16	0.29	0.01	0.00	0.00	0.00	N.D	N.D	0.02	N.D	0.02	13.40
618	Quick Whole grain rolled oats	Grain Products	78.15	cluster2	13.51	0.10	0.10	0.14	0.21	0.01	0.00	0.00	0.00	N.D	N.D	0.02	N.D	0.01	14.09
619	Peanut butter (MaraNatha)	Beans, Peas, Legumes, Nuts, Seeds	0.30	cluster2	6.88	0.69	1.19	0.31	2.61	0.10	0.05	0.01	0.09	N.D	N.D	0.07	N.D	0.04	12.03
620	Cashew butter (Artisana Organics)	Beans, Peas, Legumes, Nuts, Seeds	1.21	cluster2	13.46	0.60	1.01	0.66	0.61	0.06	0.03	0.00	0.06	N.D	N.D	0.04	N.D	0.05	16.59
621	Cashew butter (Once again)	Beans, Peas, Legumes,	0.12	cluster2	12.24	0.43	0.87	0.13	0.72	0.07	0.02	0.00	0.06	N.D	N.D	0.04	N.D	0.04	14.62

		Nuts, Seeds																	
622	Kale snacks	Vegetables	2.03	cluster2	11.24	1.40	N.D	0.50	1.50	N.D	0.26	N.D	1.75	N.D	N.D	0.36	N.D	0.06	17.07
623	Cashew	Beans, Peas, Legumes, Nuts, Seeds	3.15	cluster2	11.45	0.74	0.80	0.11	0.65	0.04	0.02	N.D	0.04	N.D	N.D	0.02	N.D	0.04	13.90
624	Black bean vegetable soup	Beans, Peas, Legumes, Nuts, Seeds	83.07	cluster2	9.00	0.69	0.34	0.25	0.71	N.D	0.04	N.D	0.21	N.D	N.D	0.08	N.D	0.02	11.34
625	Split pea soup	Beans, Peas, Legumes, Nuts, Seeds	87.97	cluster2	6.35	0.51	0.18	0.03	0.39	N.D	0.02	N.D	0.12	N.D	N.D	0.00	N.D	0.02	7.62
626	Vegetable soup	Vegetables	84.98	cluster2	7.41	0.27	0.08	0.11	0.33	N.D	0.02	N.D	0.18	N.D	N.D	0.05	N.D	0.02	8.48
627	Frozen whole cherry	Fruits	79.20	cluster2	6.81	0.23	0.37	0.07	0.41	N.D	0.10	N.D	0.41	N.D	N.D	0.06	N.D	0.01	8.46
628	Frozen mango flesh	Fruits	81.22	cluster2	9.51	0.14	0.28	0.08	0.17	N.D	0.02	N.D	0.24	N.D	N.D	0.03	N.D	0.01	10.48
629	Frozen pineapple flesh	Fruits	83.42	cluster2	7.30	0.22	0.31	0.23	0.17	N.D	0.01	N.D	0.03	N.D	N.D	0.06	N.D	0.01	8.33
630	Frozen corn	Vegetables	75.57	cluster2	9.07	0.27	0.95	0.23	0.26	N.D	0.01	N.D	0.03	N.D	N.D	N.D	N.D	0.02	10.84
631	Pomegranate w/ seeds	Fruits	75.43	cluster2	8.71	0.22	1.15	0.04	0.23	N.D	0.04	N.D	0.26	N.D	N.D	N.D	N.D	0.01	10.66
632	Black bean burger patty	Beans, Peas, Legumes, Nuts, Seeds	43.72	cluster2	14.92	1.04	1.22	0.31	1.30	N.D	0.09	N.D	0.44	N.D	N.D	N.D	N.D	0.04	19.36

633	Pea protein burger patty	Beans, Peas, Legumes, Nuts, Seeds	36.28	cluster2	7.15	0.65	0.85	0.13	0.59	N.D	0.02	N.D	0.11	N.D	N.D	N.D	N.D	0.07	9.57
634	Cauliflower burger patty	Beans, Peas, Legumes, Nuts, Seeds	39.89	cluster2	6.64	1.27	0.89	0.19	0.74	N.D	0.06	N.D	0.33	N.D	N.D	N.D	N.D	0.05	10.17
635	Mixed seed burger patty	Grain Products	45.05	cluster2	8.59	0.35	0.43	0.12	0.28	N.D	0.04	N.D	0.15	N.D	N.D	N.D	N.D	0.02	9.99
636	Beet and bean burger patty	Beans, Peas, Legumes, Nuts, Seeds	49.80	cluster2	13.25	0.63	0.19	0.18	0.61	N.D	0.04	N.D	0.27	N.D	N.D	N.D	N.D	0.02	15.18
637	Mixed vegetable burger patty	Vegetables	47.16	cluster2	13.83	1.24	0.25	0.26	0.43	N.D	0.05	N.D	0.34	N.D	N.D	N.D	N.D	0.03	16.44
638	Bean and corn burger patty	Grain Products	39.93	cluster2	11.88	0.39	0.16	0.17	0.59	N.D	0.05	N.D	0.25	N.D	N.D	N.D	N.D	0.02	13.49
639	Whole grain millet sweet potato and leafy greens burger patty	Grain Products	39.69	cluster2	11.78	0.24	0.20	0.11	0.13	N.D	0.03	N.D	0.09	N.D	N.D	N.D	N.D	0.01	12.58
640	Cream of Mushroom Soup (Pacific Foods)	Vegetables	89.50	cluster2	7.73	0.20	0.10	0.00	0.03	0.00	0.00	0.00	0.00	N.D	N.D	0.03	N.D	0.00	8.10
641	Cream of Chicken Soup	Meat, Poultry, Fish, and Mixtures	86.53	cluster2	6.81	0.15	0.04	0.00	0.02	0.00	0.00	0.00	0.00	N.D	N.D	0.01	N.D	0.01	7.04
642	Vegan Split Pea Soup	Beans, Peas,	79.26	cluster2	9.97	0.61	0.25	0.03	0.56	0.02	0.03	0.01	0.20	N.D	N.D	0.08	N.D	0.02	11.77

		Legumes, Nuts, Seeds																		
643	13 Beans Soup Mix	Beans, Peas, Legumes, Nuts, Seeds	78.27	cluster2	7.82	0.78	0.22	0.18	3.06	0.03	0.02	0.01	0.02	N.D	N.D	0.11	N.D	0.02	12.27
644	Frozen Whole Sweet Green Peas (Flav R Pac)	Beans, Peas, Legumes, Nuts, Seeds	78.26	cluster2	6.85	0.24	0.94	0.05	0.78	0.02	0.03	0.00	0.15	N.D	N.D	N.D	N.D	0.04	9.10
645	Frozen Sweet Peas (Cascadian)	Beans, Peas, Legumes, Nuts, Seeds	77.52	cluster2	11.90	0.38	4.23	0.23	1.07	0.02	0.03	0.00	0.17	N.D	N.D	0.00	N.D	0.04	18.07
646	Frozen Whole Green Peas (Signature Select)	Beans, Peas, Legumes, Nuts, Seeds	56.14	cluster2	11.62	0.55	0.17	0.15	1.63	0.06	0.07	0.00	0.31	N.D	N.D	0.01	N.D	0.07	14.65
647	Frozen Whole Green Peas (Stahlbush)	Beans, Peas, Legumes, Nuts, Seeds	76.79	cluster2	6.51	0.29	0.29	0.06	0.61	0.03	0.03	0.00	0.14	N.D	N.D	N.D	N.D	0.04	8.01
648	Refried Beans	Beans, Peas, Legumes, Nuts, Seeds	78.39	cluster2	7.27	0.41	0.23	0.23	0.85	0.06	0.02	0.01	0.05	0.01	N.D	0.11	N.D	0.01	9.26
649	Vanilla bean ice cream	Milk and Milk Products	56.70	cluster2	9.26	1.74	0.73	0.00	0.02	0.06	0.00	0.00	0.00	N.D	N.D	0.18	N.D	0.01	12.01

650	Vanilla rice drink	Grain Products	88.97	cluster2	8.12	0.05	0.06	0.01	0.02	0.00	0.00	0.00	0.00	0.00	N.D	0.00	N.D	0.00	8.27
651	Enriched rice drink	Grain Products	89.38	cluster2	7.56	0.05	0.06	0.00	0.01	0.00	0.00	0.00	0.00	0.00	N.D	0.00	N.D	0.00	7.68
652	Enriched vanilla rice drink	Grain Products	87.81	cluster2	7.66	0.05	0.06	0.00	0.02	0.00	0.00	0.00	0.00	0.00	N.D	0.01	N.D	0.00	7.80
653	Tomato Ketchup	Vegetables	58.93	cluster2	12.65	0.21	2.70	0.02	0.05	N.D	0.02	N.D	0.27	0.02	N.D	N.D	N.D	0.00	15.95
654	Refried Beans	Beans, Peas, Legumes, Nuts, Seeds	73.17	cluster2	8.48	0.50	0.18	0.23	0.91	0.05	0.03	0.01	0.06	0.00	N.D	0.15	0.00	0.02	10.63
655	Cannellini Beans	Beans, Peas, Legumes, Nuts, Seeds	74.85	cluster2	8.20	0.66	0.16	0.21	0.80	0.06	0.03	0.00	0.22	0.01	N.D	0.12	N.D	0.02	10.51
656	Refried black beans	Beans, Peas, Legumes, Nuts, Seeds	73.04	cluster2	8.20	0.48	0.17	0.26	0.93	0.05	0.03	0.01	0.08	0.00	0.00	0.14	0.00	0.02	10.37
657	Almond butter protein bar	Beans, Peas, Legumes, Nuts, Seeds	4.07	cluster2	11.33	2.43	1.12	0.27	1.25	0.04	0.07	0.00	0.11	0.01	N.D	0.10	N.D	0.04	16.79
658	Peanut butter protein bar	Beans, Peas, Legumes, Nuts, Seeds	1.27	cluster2	12.40	3.19	2.35	0.30	1.04	0.05	0.05	0.01	0.06	0.01	N.D	0.10	N.D	0.04	19.59
659	Unsweetened pitted prunes	Fruits	8.94	cluster2	13.36	1.49	2.05	0.16	0.78	0.09	0.11	N.D	1.39	N.D	N.D	0.28	N.D	0.01	19.71

660	Rice ramen	Grain Products	82.72	cluster2	13.55	0.08	0.11	0.05	0.11	0.00	0.00	0.00	0.00	0.00	N.D	0.01	N.D	0.01	13.92
661	Chickpea mac & cheese pasta	Beans, Peas, Legumes, Nuts, Seeds	74.67	cluster2	14.51	0.77	0.11	0.07	0.82	0.01	0.02	0.01	0.03	0.00	N.D	0.08	N.D	0.01	16.44
662	Whole double fiber bread	Grain Products	27.15	cluster2	34.98	0.21	0.35	1.45	1.18	0.10	0.01	0.01	0.01	N.D	N.D	0.14	N.D	0.04	38.48
663	Whole whole grain bread	Grain Products	26.86	cluster2	30.38	0.49	0.24	1.03	1.95	0.11	0.01	0.01	0.02	N.D	N.D	0.15	N.D	0.04	34.45
664	Inside only Whole grain bread	Grain Products	26.54	cluster2	32.55	0.43	0.30	1.06	2.00	0.08	0.01	0.01	0.02	N.D	N.D	0.15	N.D	0.04	36.65
665	Whole Whole Grains English muffin (Oroweat)	Grain Products	28.54	cluster2	32.94	0.11	0.50	0.59	0.63	0.09	0.00	0.00	N.D	N.D	N.D	0.14	N.D	0.04	35.05
666	Whole Double Fiber English muffin (Oroweat)	Grain Products	31.95	cluster2	34.30	0.11	0.55	0.92	0.90	0.08	0.00	0.01	N.D	N.D	N.D	0.13	N.D	0.03	37.04
667	Yellow corn tortilla	Grain Products	41.64	cluster2	28.10	0.44	0.16	0.45	0.66	0.01	0.00	N.D	0.01	N.D	N.D	0.18	N.D	0.02	30.02
668	White corn tortilla	Grain Products	42.66	cluster2	30.55	0.45	0.18	0.38	0.65	0.03	0.00	N.D	0.01	N.D	N.D	0.16	N.D	0.02	32.43
669	Whole wheat tortilla	Grain Products	30.83	cluster2	30.62	N.D	0.20	0.97	1.14	0.03	0.00	N.D	0.01	N.D	N.D	0.06	N.D	0.03	33.05
670	Raspberry jam (Bonne Maman)	Fruits	30.62	cluster2	27.15	0.06	10.89	0.11	0.08	N.D	0.02	N.D	0.26	N.D	N.D	N.D	N.D	0.01	38.59
671	Orange jam (Bonne Maman)	Sugars, Sweets, and Beverages	27.88	cluster2	32.72	0.13	13.28	0.06	0.08	N.D	0.05	N.D	0.23	0.06	0.02	N.D	N.D	0.01	46.65
672	Fig jam (Bonne Maman)	Fruits	31.16	cluster2	34.36	0.04	13.60	0.02	0.07	N.D	0.02	N.D	0.28	0.04	0.02	N.D	N.D	0.01	48.46

673	Strawberry jam (Bonne Maman)	Fruits	29.61	cluster2	30.28	0.05	12.29	0.05	0.05	N.D	0.01	N.D	0.07	0.03	0.01	N.D	N.D	0.01	42.84
674	Plum jam (Bonne Maman)	Fruits	27.68	cluster2	30.76	0.06	10.38	0.03	0.05	N.D	0.01	N.D	0.08	0.05	0.02	N.D	N.D	0.01	41.45
675	Mango Peach jam (Bonne Maman)	Fruits	27.48	cluster2	28.88	0.09	11.70	0.06	0.13	N.D	0.02	N.D	0.27	N.D	N.D	N.D	N.D	0.01	41.16
676	Nutella spread	Beans, Peas, Legumes, Nuts, Seeds	0.20	cluster2	31.83	2.96	15.25	0.09	0.41	N.D	0.04	N.D	0.20	N.D	N.D	0.20	0.01	N.D	51.00
677	Baking flour	Beans, Peas, Legumes, Nuts, Seeds	9.09	cluster2	27.39	0.41	0.39	0.15	1.73	0.09	0.05	0.01	0.09	N.D	N.D	2.06	N.D	0.03	32.40
678	Brown Rice (Minute)	Grain Products	58.79	cluster2	33.54	0.10	0.19	0.13	0.25	0.02	0.00	0.00	0.01	N.D	N.D	0.02	N.D	0.02	34.27
679	7 grains mix	Grain Products	59.44	cluster2	31.11	0.32	0.05	0.84	0.52	N.D	0.01	N.D	N.D	N.D	N.D	0.04	N.D	N.D	32.89
680	Grains and vegetable mix	Grain Products	64.75	cluster2	33.15	0.37	N.D	0.10	0.23	N.D	0.00	N.D	N.D	N.D	N.D	0.02	N.D	N.D	33.87
681	Spaghetti	Grain Products	56.20	cluster2	27.73	0.20	0.14	0.35	0.62	0.02	0.00	0.00	0.00	N.D	N.D	0.04	N.D	0.02	29.12
682	Whole wheat spaghetti	Grain Products	64.20	cluster2	32.76	0.17	N.D	0.05	0.10	N.D	0.00	N.D	0.00	N.D	N.D	0.05	N.D	N.D	33.13
683	Whole grain amaranth	Grain Products	58.95	cluster2	28.53	0.40	N.D	0.23	0.72	N.D	0.10	N.D	0.42	N.D	N.D	0.05	N.D	0.04	30.49
684	Whole grain khorasan kamut	Grain Products	51.68	cluster2	30.05	0.16	0.25	0.61	0.84	0.02	0.00	0.00	0.00	N.D	N.D	0.04	N.D	0.02	32.01
685	Brown rice hot cereal	Grain Products	64.70	cluster2	29.07	0.14	0.28	0.10	0.27	0.01	0.00	0.00	0.00	N.D	N.D	0.03	N.D	0.01	29.91

686	Beet chips	Vegetables	0.48	cluster2	28.68	1.68	1.54	4.24	4.00	N.D	0.37	N.D	1.41	N.D	N.D	0.30	N.D	0.08	42.30
687	Freeze dried strawberry	Fruits	0.90	cluster2	33.28	1.88	5.04	0.74	0.88	N.D	0.14	N.D	1.96	N.D	N.D	0.02	N.D	0.07	44.01
688	Freeze dried pinepapple rings	Fruits	9.37	cluster2	31.00	1.07	1.27	1.30	0.93	N.D	0.03	N.D	0.27	N.D	N.D	0.61	N.D	0.03	36.52
689	Dried coconut strips	Fruits	2.51	cluster2	33.51	1.10	1.10	N.D	0.18	N.D	0.04	N.D	0.08	N.D	N.D	2.67	N.D	0.01	38.69
690	Dried watermelon	Fruits	3.31	cluster2	29.13	0.55	7.23	0.35	0.46	N.D	0.07	N.D	0.86	N.D	N.D	0.46	N.D	0.04	39.15
691	Multigrain bread (Trade Joes)	Grain Products	36.69	cluster2	33.11	N.D	0.69	0.67	1.75	0.03	0.00	0.00	0.01	N.D	N.D	0.12	N.D	0.03	36.41
692	Sprouted wheat bread (Silver Hills)	Grain Products	37.21	cluster2	31.65	N.D	0.70	0.85	1.64	0.03	0.00	0.00	0.01	N.D	N.D	0.12	N.D	0.03	35.04
693	Buckwheat Molasses bread	Grain Products	33.11	cluster2	33.02	N.D	0.54	0.46	1.53	0.05	0.04	0.03	0.03	N.D	N.D	0.37	N.D	0.03	36.11
694	Millet and chia bread	Grain Products	30.05	cluster2	31.64	N.D	0.90	0.28	0.73	0.04	0.02	0.01	0.02	N.D	N.D	0.20	N.D	0.03	33.87
695	Sprouted grain bread	Grain Products	38.35	cluster2	32.23	N.D	0.80	0.82	1.40	0.03	0.00	0.00	0.01	N.D	N.D	0.15	N.D	0.03	35.47
696	Whole grain bread (Artisan Bakers)	Grain Products	34.82	cluster2	30.94	N.D	0.35	0.52	1.01	0.04	0.00	0.00	0.01	N.D	N.D	0.14	N.D	0.03	33.06
697	Multigrain bread (Alvarado St. Bakery)	Grain Products	37.31	cluster2	31.06	N.D	0.55	0.66	1.48	0.03	0.00	0.00	0.00	N.D	N.D	0.12	N.D	0.03	33.94
698	Whole grain bread (Happy Campers)	Grain Products	41.17	cluster2	32.82	0.27	0.23	0.34	1.03	0.03	0.04	0.03	0.02	N.D	N.D	0.34	N.D	0.03	35.19
699	Sesame bread	Grain Products	38.99	cluster2	27.63	0.56	0.21	0.85	1.92	0.02	0.01	0.01	0.01	N.D	N.D	0.11	N.D	0.03	31.36
700	Flax bread (Udls)	Grain Products	30.14	cluster2	32.62	0.50	0.55	0.16	0.54	0.08	0.03	0.01	0.05	N.D	N.D	0.28	N.D	0.03	34.84

701	Sprouted wheat bread (Trader Joes)	Grain Products	36.66	cluster2	31.86	0.19	0.30	0.86	2.11	0.02	0.00	0.01	0.00	N.D	N.D	0.12	N.D	0.03	35.51
702	Mini Cheddar Cheese Biscuit	Grain Products	0.15	cluster2	32.59	2.05	0.40	0.24	0.38	0.01	0.00	0.00	0.00	N.D	N.D	0.09	N.D	0.02	35.79
703	Flax Plus Waffle	Grain Products	34.07	cluster2	31.40	0.59	0.28	0.38	0.45	0.05	0.01	0.00	0.02	N.D	N.D	0.07	N.D	0.01	33.27
704	Maple syrup	Sugars, Sweets, and Beverages	27.05	cluster2	31.85	0.03	3.72	0.01	0.07	0.21	0.00	0.00	0.00	N.D	N.D	0.07	N.D	0.00	35.95
705	Honey	Sugars, Sweets, and Beverages	25.08	cluster2	28.66	0.05	6.09	0.00	0.13	0.11	0.00	0.00	0.00	N.D	N.D	0.05	N.D	0.00	35.10
706	Wheat bran cereal	Grain Products	2.77	cluster2	28.65	0.65	1.27	2.41	2.13	0.02	0.02	0.02	0.02	0.00	N.D	0.14	N.D	0.06	35.39
707	Whole wheat bread	Grain Products	30.57	cluster2	32.32	0.46	0.61	1.61	0.83	0.01	0.01	0.01	0.01	0.00	N.D	0.16	N.D	0.02	36.06
708	Sprouted whole grain bread	Grain Products	30.05	cluster2	32.57	0.48	0.73	1.37	0.77	0.01	0.01	0.01	0.01	0.00	N.D	0.18	N.D	0.03	36.15
709	Whole grains and seeds bread	Grain Products	38.01	cluster2	30.30	0.36	0.82	1.20	0.91	0.01	0.01	0.01	0.01	0.00	N.D	0.15	N.D	0.02	33.80
710	Stir fry rice noodle	Grain Products	62.27	cluster2	31.74	0.20	0.19	0.03	0.08	0.00	0.00	0.00	0.00	0.00	N.D	0.02	N.D	0.01	32.28
711	Chicken breast tender	Meat, Poultry, Fish, and Mixtures	52.24	cluster2	32.74	0.20	0.32	0.03	0.10	0.01	0.00	0.01	0.01	0.00	N.D	0.04	N.D	0.12	33.58
712	Short grain brown rice	Grain Products	56.42	cluster2	33.19	0.16	0.22	0.07	0.12	0.01	0.00	0.00	0.00	0.00	N.D	0.04	N.D	0.01	33.82
713	Whole buttermilk bread	Grain Products	26.00	cluster3	46.83	1.18	0.30	0.41	0.54	0.06	0.00	N.D	0.00	N.D	N.D	0.18	N.D	0.03	49.53

714	Whole potato bread	Grain Products	26.38	cluster3	48.20	0.43	0.47	0.43	0.62	0.04	0.00	0.00	0.01	N.D	N.D	0.16	N.D	0.03	50.40
715	Strawberry Jam (Smuckers)	Fruits	29.61	cluster3	51.44	0.09	6.75	0.05	0.07	N.D	0.01	0.02	0.14	0.06	0.02	N.D	N.D	0.01	58.67
716	7 grain pancake/waffle flour	Grain Products	8.98	cluster3	47.13	0.32	0.43	1.34	1.94	0.04	0.01	0.02	0.01	N.D	N.D	0.13	N.D	0.04	51.40
717	Freeze dried mango flesh	Fruits	2.59	cluster3	48.51	0.83	2.77	0.41	1.04	N.D	0.10	N.D	1.14	N.D	N.D	N.D	N.D	0.04	54.84
718	Whole grain cereal (Trader Joes)	Grain Products	1.29	cluster3	48.97	0.33	3.42	1.13	1.98	0.08	0.00	0.01	N.D	N.D	N.D	0.06	N.D	0.03	56.00
719	Puffin cereal (Barbaras)	Grain Products	10.21	cluster3	46.93	0.20	2.19	1.05	0.74	0.06	0.01	0.01	N.D	N.D	N.D	0.07	N.D	0.03	51.28
720	Multigrain crackers	Grain Products	1.87	cluster3	51.33	0.67	2.33	0.81	1.29	0.08	0.01	0.00	N.D	N.D	N.D	0.10	N.D	0.03	56.65
721	Pita chips	Grain Products	0.33	cluster3	48.56	0.23	0.78	1.05	1.00	0.04	0.01	0.01	0.01	N.D	N.D	0.11	N.D	0.03	51.83
722	Dried mango (Mariani)	Fruits	2.21	cluster3	44.34	0.48	4.89	0.16	0.36	N.D	0.04	N.D	0.81	N.D	N.D	N.D	N.D	0.03	51.13
723	Dried orange slices	Fruits	9.60	cluster3	45.45	0.66	2.52	0.08	0.38	N.D	0.13	N.D	0.58	N.D	N.D	0.26	N.D	0.01	50.08
724	Almond cookies	Grain Products	1.98	cluster3	50.56	0.47	2.47	0.39	0.82	0.08	0.01	0.00	N.D	N.D	N.D	0.08	N.D	0.03	54.89
725	Chocolate ship cookies	Grain Products	2.51	cluster3	44.73	0.14	3.29	0.31	0.48	0.07	0.00	0.00	N.D	N.D	N.D	0.24	N.D	0.02	49.29
726	Honey graham crackers	Grain Products	3.02	cluster3	51.38	0.71	2.75	0.84	1.06	0.06	0.00	0.00	N.D	N.D	N.D	0.15	N.D	0.02	56.98
727	Golden round crackers	Grain Products	3.63	cluster3	49.45	0.76	1.07	0.39	0.62	0.04	0.00	0.00	N.D	N.D	N.D	0.09	N.D	0.02	52.45
728	Peanut butter crackers	Grain Products	2.58	cluster3	49.30	0.63	1.46	0.31	0.68	0.07	0.00	0.00	0.01	N.D	N.D	0.07	N.D	0.02	52.57
729	Brown rice bread	Grain Products	41.91	cluster3	46.95	0.57	0.06	0.19	0.43	0.14	0.01	0.02	0.02	N.D	N.D	0.24	N.D	0.02	48.67
730	Mini Peanut Butter Biscuit	Grain Products	2.05	cluster3	50.64	0.44	0.46	0.36	0.73	0.05	0.02	0.00	0.02	N.D	N.D	0.12	N.D	0.01	52.86

731	Gluten-Free Chia Plus Waffle	Grain Products	31.81	cluster3	48.56	0.50	0.29	0.05	0.17	0.05	0.01	0.00	0.01	N.D	N.D	0.02	N.D	0.01	49.68
732	Table sugar*	Sugars, Sweets, and Beverages	0.01	cluster3	46.78	0.14	38.81	0.00	0.06	0.17	0.00	0.00	0.00	N.D	N.D	0.03	N.D	0.00	52.91
733	Sourdough bread	Grain Products	28.31	cluster3	50.60	0.53	0.48	0.67	0.62	0.02	0.00	0.00	0.00	N.D	N.D	0.09	N.D	0.01	53.02
734	Chickpea veggie crisps	Beans, Peas, Legumes, Nuts, Seeds	0.97	cluster3	50.36	1.15	0.49	0.20	1.52	0.03	0.04	0.01	0.07	0.00	N.D	0.11	N.D	0.05	54.02
735	Blue corn tortilla chips	Grain Products	0.49	cluster3	50.88	0.43	0.48	0.51	0.71	0.01	0.01	0.01	0.02	0.00	N.D	0.04	N.D	0.01	53.12
736	Tortilla chips	Grain Products	1.31	cluster3	51.03	0.53	0.41	0.48	0.72	0.01	0.01	0.01	0.01	0.00	N.D	0.05	N.D	0.01	53.27
737	Whole wheat flour	Grain Products	7.85	cluster3	48.19	0.71	0.48	0.98	0.92	0.01	0.00	0.01	0.01	0.00	N.D	0.12	N.D	0.03	51.45
738	Red lentil rotini pasta	Beans, Peas, Legumes, Nuts, Seeds	59.84	cluster3	51.09	1.14	0.42	0.14	1.22	0.02	0.03	0.01	0.03	0.00	N.D	0.06	N.D	0.05	54.22
739	Almond cereal	Grain Products	1.45	cluster3	48.99	0.43	0.53	0.60	0.79	0.04	0.03	0.01	0.04	0.00	N.D	0.12	N.D	0.03	51.62
740	Whole white bread	Grain Products	28.88	cluster3	44.12	0.01	0.31	0.39	1.00	0.07	0.00	0.00	0.00	N.D	N.D	0.16	N.D	0.03	46.10
741	Crust only whole grain bread	Grain Products	14.99	cluster3	36.72	0.24	0.32	1.47	2.83	0.06	0.02	0.02	0.03	N.D	N.D	0.22	N.D	0.05	41.97
742	Whole oatnut bread	Grain Products	20.71	cluster3	37.63	0.38	0.49	0.39	0.91	0.10	0.01	0.00	0.02	N.D	N.D	0.26	N.D	0.04	40.24
743	Whole Extra Crisp English	Grain Products	36.14	cluster3	44.33	0.08	0.40	0.35	0.64	0.04	0.00	0.00	N.D	N.D	N.D	0.18	N.D	0.03	46.06

	muffin (Oroweat)																		
744	Whole Original English muffin (Thomas)	Grain Products	27.90	cluster3	45.81	0.15	0.53	0.41	0.55	0.06	0.00	0.00	N.D	N.D	N.D	0.12	N.D	0.02	47.65
745	Whole Light Multi Grain English muffin	Grain Products	31.76	cluster3	36.76	0.23	0.24	0.48	0.46	0.02	0.00	N.D	N.D	N.D	N.D	0.12	N.D	0.03	38.35
746	Flour tortilla	Grain Products	31.22	cluster3	36.49	0.22	0.26	0.39	0.52	0.03	0.00	N.D	N.D	N.D	N.D	0.16	N.D	0.02	38.10
747	Brown Rice (Organics)	Grain Products	55.48	cluster3	37.86	0.30	N.D	0.11	0.15	N.D	0.00	N.D	0.01	N.D	N.D	0.01	N.D	N.D	38.44
748	White rice (Minute)	Grain Products	59.49	cluster3	37.61	0.24	N.D	0.05	0.08	N.D	0.00	N.D	0.01	N.D	N.D	0.02	N.D	N.D	38.02
749	Whole wheat penne rigate	Grain Products	54.68	cluster3	38.40	0.34	0.30	1.40	1.01	N.D	0.01	0.01	0.02	N.D	N.D	0.10	N.D	0.02	41.61
750	Raisin bran cereal	Grain Products	3.23	cluster3	44.75	0.23	1.59	1.57	1.78	0.05	0.01	0.01	N.D	N.D	N.D	0.08	N.D	0.04	50.11
751	Wheat crackers (Nabisco)	Grain Products	1.87	cluster3	37.93	0.61	1.88	0.92	1.66	0.06	0.00	0.00	N.D	N.D	N.D	0.07	N.D	0.03	43.15
752	Cauliflower snacks	Vegetable s	1.95	cluster3	41.53	0.63	N.D	0.25	0.73	N.D	0.09	N.D	0.32	N.D	N.D	0.33	N.D	0.05	43.92
753	Dried phiilippines mango (Mariani)	Fruits	4.38	cluster3	38.58	0.45	4.96	0.22	0.66	N.D	0.07	N.D	0.64	N.D	N.D	0.15	N.D	0.03	45.76
754	Freeze dried mandarins	Fruits	5.88	cluster3	39.82	0.95	2.32	0.10	0.83	N.D	0.27	N.D	1.11	N.D	N.D	0.12	N.D	0.02	45.54
755	Cheesy crackers	Milk and Milk Products	2.27	cluster3	38.28	0.32	0.49	0.32	0.69	0.05	0.00	0.00	N.D	N.D	N.D	0.05	N.D	0.02	40.23
756	Bean chips	Beans, Peas, Legumes, Nuts, Seeds	0.89	cluster3	41.13	1.64	0.63	0.58	5.00	0.25	0.05	0.05	0.14	N.D	N.D	0.21	N.D	0.06	49.75

757	Sea Salt Mini Rice Cake	Grain Products	0.82	cluster3	39.75	0.10	0.12	0.18	0.29	0.02	0.00	0.00	0.00	N.D	N.D	0.03	N.D	0.01	40.51
758	Organic Honey Flakes & Oat Cluster Cereals	Grain Products	2.93	cluster3	42.50	0.42	2.31	0.24	0.68	0.01	0.00	0.00	0.00	N.D	N.D	N.D	N.D	0.01	46.18
759	Original Go Rise Cereals	Grain Products	3.79	cluster3	37.19	1.61	0.34	2.18	1.73	0.12	0.05	0.02	0.07	N.D	N.D	0.18	N.D	0.04	43.54
760	Granola	Grain Products	0.59	cluster3	45.44	0.53	0.80	0.41	0.46	0.09	0.01	0.01	0.01	N.D	N.D	0.11	N.D	0.03	47.90
761	White bean chips	Beans, Peas, Legumes, Nuts, Seeds	0.40	cluster3	39.00	0.75	0.37	0.24	1.54	0.08	0.03	0.02	0.06	0.00	N.D	0.20	N.D	0.03	42.34
762	Super seed crackers	Grain Products	1.05	cluster3	36.37	0.42	0.39	0.45	0.71	0.04	0.08	0.01	0.09	0.00	N.D	0.23	N.D	0.03	38.81
763	Hummus crisps w/ rosemary and olive oil	Beans, Peas, Legumes, Nuts, Seeds	2.00	cluster3	43.20	1.32	0.53	0.13	1.47	0.02	0.05	0.02	0.06	0.00	N.D	0.07	N.D	0.04	46.90
764	Good seed bread	Grain Products	26.72	cluster3	38.35	0.47	1.01	0.81	0.73	0.02	0.02	0.00	0.04	0.00	N.D	0.24	N.D	0.03	41.73
765	White bread	Grain Products	35.71	cluster3	36.53	0.29	0.48	0.60	0.68	0.01	0.00	0.00	0.01	0.00	N.D	0.16	N.D	0.02	38.80
766	Bean and cheese burrito	Grain Products	52.06	cluster3	40.99	1.18	0.52	0.92	3.23	0.12	0.06	0.03	0.08	0.01	0.01	0.32	N.D	0.05	47.51
767	Righteous rye bread	Grain Products	28.61	cluster3	36.76	0.45	0.79	0.81	0.70	0.01	0.01	0.01	0.02	0.00	N.D	0.34	N.D	0.04	39.95
768	Peanut butter pretzel	Grain Products	2.44	cluster3	44.95	0.68	0.66	0.51	0.91	0.04	0.04	0.01	0.08	0.00	N.D	0.12	N.D	0.02	48.03
769	Cauliflower tortillas	Vegetables	27.51	cluster3	41.65	0.89	N.D	0.08	0.36	0.03	0.02	0.01	0.17	0.00	N.D	0.60	N.D	0.01	43.84
770	Chocolate chip cookies	Grain Products	1.92	cluster3	67.88	0.57	1.65	0.54	0.57	N.D	0.01	N.D	0.05	N.D	N.D	N.D	0.01	N.D	71.27
771	Corn cereal (corn flakes)	Grain Products	1.47	cluster3	69.40	0.30	N.D	0.12	0.24	N.D	0.00	N.D	0.01	N.D	N.D	N.D	N.D	N.D	70.07

772	Honey nut oat cereal	Grain Products	1.69	cluster3	70.13	0.91	6.62	1.28	1.05	N.D	0.01	N.D	0.07	N.D	N.D	0.07	0.04	N.D	80.19
773	Buttermilk pancake/waffle flour	Grain Products	7.27	cluster3	70.52	3.37	N.D	1.71	1.43	N.D	N.D	N.D	0.01	N.D	N.D	0.16	0.03	N.D	77.23
774	Multigrain cereal (Gerber)	Grain Products	3.20	cluster3	71.66	1.05	N.D	3.08	2.58	N.D	0.01	N.D	0.04	N.D	N.D	0.36	N.D	N.D	78.78
775	Whole wheat cereal (Gerber)	Grain Products	3.33	cluster3	71.71	1.11	N.D	5.45	3.96	N.D	0.02	N.D	0.04	N.D	N.D	0.42	N.D	N.D	82.72
776	Multigrain cereal (Earth's best)	Grain Products	5.05	cluster3	68.64	0.73	N.D	0.81	0.75	N.D	0.01	N.D	0.02	N.D	N.D	0.32	N.D	N.D	71.27
777	Corn flakes cereal	Grain Products	3.87	cluster3	69.92	0.27	1.06	0.10	0.27	0.03	0.00	0.00	N.D	N.D	N.D	0.04	N.D	0.02	71.72
778	Thin crackers	Grain Products	1.85	cluster3	65.91	0.24	0.76	0.13	0.82	0.04	0.02	0.00	N.D	N.D	N.D	0.52	N.D	0.04	68.50
779	Cinnamon Toast Rice Cake	Grain Products	7.50	cluster3	69.51	0.18	1.32	0.41	1.12	0.02	0.01	0.00	0.02	N.D	N.D	0.07	N.D	0.03	72.71
780	5 Grain Rice Cake	Grain Products	5.44	cluster3	67.92	0.21	0.63	0.31	1.26	0.03	0.03	0.00	0.01	N.D	N.D	0.07	N.D	0.04	70.49
781	Salt-free Brown Rice Rice Cake	Grain Products	6.88	cluster3	72.31	0.21	0.53	0.43	0.87	0.04	0.01	0.01	0.01	N.D	N.D	0.09	N.D	0.03	74.53
782	Apple Pie Mini Rice Cake	Grain Products	1.39	cluster3	65.74	0.64	0.24	0.26	0.45	0.03	0.01	0.00	0.01	N.D	N.D	0.06	N.D	0.01	67.46
783	Thin Almond Nut Rice Crackers	Grain Products	0.38	cluster3	66.15	N.D	0.59	0.10	1.71	0.02	0.02	0.01	0.02	N.D	N.D	0.05	N.D	0.03	68.71
784	Organic Frosted Flakes Cereals	Grain Products	2.48	cluster3	67.08	0.16	4.23	0.07	0.56	0.04	0.00	0.00	0.00	N.D	N.D	0.04	N.D	0.00	72.19
785	Organic Morning O's Cereals	Grain Products	3.71	cluster3	66.58	0.99	0.60	1.10	3.64	0.07	0.02	0.01	0.01	N.D	N.D	0.13	N.D	0.05	73.18
786	Cornmeal	Grain Products	10.77	cluster3	67.37	0.07	0.66	0.39	0.65	0.02	0.01	0.00	0.01	N.D	N.D	0.05	N.D	0.01	69.24
787	Jolly rancher candy	Sugars, Sweets,	0.04	cluster3	66.05	0.15	4.23	0.00	0.09	0.08	0.00	0.00	0.00	N.D	N.D	0.02	N.D	0.00	70.63

		and Beverages																	
788	Puffed rice cereal	Grain Products	4.94	cluster3	69.47	0.46	0.50	0.31	0.44	0.01	0.01	0.01	0.02	0.00	N.D	0.05	N.D	0.02	71.29
789	Vanilla cookies	Grain Products	1.29	cluster3	52.98	0.31	0.68	0.27	0.58	0.10	0.00	N.D	N.D	N.D	N.D	0.10	N.D	0.03	55.04
790	Multi grain cereal	Grain Products	2.57	cluster3	56.03	0.67	3.66	0.71	0.70	N.D	0.01	N.D	0.03	N.D	N.D	N.D	0.03	N.D	61.81
791	Whole grain oat cereal	Grain Products	1.86	cluster3	61.12	1.07	3.33	0.33	0.46	N.D	0.00	N.D	0.03	N.D	N.D	N.D	0.05	N.D	66.40
792	Whole grain oatmeal cereal (Earth's best)	Grain Products	2.78	cluster3	53.55	0.39	0.46	0.72	1.45	0.08	0.01	0.01	0.01	N.D	N.D	0.10	N.D	0.06	56.84
793	Puffed Kamut cereal	Grain Products	5.53	cluster3	55.57	0.20	0.84	1.47	1.33	0.03	0.00	0.01	0.01	N.D	N.D	0.08	N.D	0.03	59.58
794	Green pea snack crisps	Beans, Peas, Legumes, Nuts, Seeds	3.55	cluster3	58.83	4.72	0.10	0.31	2.99	N.D	0.17	N.D	0.60	N.D	N.D	N.D	N.D	0.12	67.85
795	Oat crunch cereal	Grain Products	3.84	cluster3	61.82	0.41	2.85	0.68	1.55	0.06	0.01	0.01	N.D	N.D	N.D	0.09	N.D	0.05	67.52
796	Multgrain cereal (Barbaras)	Grain Products	4.13	cluster3	55.24	0.32	2.33	0.76	1.14	0.04	0.01	0.01	N.D	N.D	N.D	0.07	N.D	0.04	59.95
797	Coconot almond chia cereal	Grain Products	1.12	cluster3	58.12	0.22	1.48	0.70	1.54	0.05	0.00	0.01	0.01	N.D	N.D	0.08	N.D	0.04	62.25
798	Wheat crackers (Organic Trsicuit)	Grain Products	3.02	cluster3	61.16	0.65	0.91	1.28	2.63	0.03	0.00	0.01	0.01	N.D	N.D	0.11	N.D	0.03	66.83
799	Pea snacks	Beans, Peas, Legumes, Nuts, Seeds	2.08	cluster3	54.52	2.74	N.D	1.96	2.93	N.D	0.24	N.D	1.14	N.D	N.D	0.08	N.D	0.10	63.70
800	Plantain chips	Fruits	1.78	cluster3	54.80	0.16	1.06	0.10	0.32	0.08	0.00	0.01	0.06	N.D	N.D	0.13	N.D	0.02	56.74

801	Banana chips	Fruits	2.24	cluster3	60.03	N.D	2.20	0.16	0.32	N.D	0.01	N.D	0.68	N.D	N.D	0.31	N.D	0.04	63.74
802	Animal shaped crackers	Grain Products	3.62	cluster3	60.47	0.21	2.27	0.42	0.56	0.07	0.00	0.00	N.D	N.D	N.D	0.12	N.D	0.03	64.16
803	Cheese crackers	Grain Products	2.70	cluster3	52.77	1.43	1.19	0.33	0.44	0.04	0.00	0.00	N.D	N.D	N.D	0.08	N.D	0.02	56.30
804	White cheddar corn puff	Grain Products	2.64	cluster3	55.76	1.99	0.79	0.21	0.61	0.05	0.01	0.01	0.01	N.D	N.D	0.04	N.D	0.03	59.50
805	Vegetable chips	Vegetables	2.11	cluster3	59.01	0.81	0.64	0.03	0.33	0.11	0.02	N.D	0.05	N.D	N.D	0.03	N.D	0.02	61.06
806	Popcorn	Grain Products	2.50	cluster3	53.64	0.24	0.56	0.43	0.84	0.03	0.00	0.01	0.01	N.D	N.D	0.04	N.D	0.03	55.83
807	Red Rice & Quinoa Rice Cake	Grain Products	5.97	cluster3	54.33	0.21	0.41	0.29	0.79	0.02	0.01	0.00	0.01	N.D	N.D	0.06	N.D	0.02	56.15
808	White Cheddar Mini Rice Cake	Grain Products	0.23	cluster3	55.37	0.94	0.44	0.35	0.72	0.04	0.01	0.00	0.02	N.D	N.D	0.07	N.D	0.03	58.00
809	Saltine Crackers	Grain Products	2.71	cluster3	56.65	0.44	0.19	0.49	0.65	0.02	0.00	0.00	0.00	N.D	N.D	0.19	N.D	0.01	58.65
810	Classic Crackers	Grain Products	3.89	cluster3	58.74	0.45	0.25	0.57	0.69	0.03	0.00	0.00	0.00	N.D	N.D	0.11	N.D	0.01	60.85
811	Sprouted Crunchy Golden Flax Cereal	Grain Products	1.44	cluster3	53.49	0.32	0.60	1.85	2.03	0.03	0.02	0.01	0.01	N.D	N.D	0.11	N.D	0.03	58.49
812	Toasted Whole Wheat Berry Flakes & Flaxseed Cereal	Grain Products	0.43	cluster3	62.27	0.36	0.98	1.95	2.27	0.01	0.01	0.01	0.00	N.D	N.D	0.14	N.D	0.02	68.02
813	Organic Morning O's Honey Nut Cereals	Grain Products	2.82	cluster3	62.01	0.13	3.86	0.56	1.45	0.02	0.01	0.01	0.01	N.D	N.D	0.09	N.D	0.05	68.20
814	Rainbow Morning O's Cereals	Grain Products	3.71	cluster3	54.46	0.18	2.87	0.41	0.99	0.05	0.01	0.00	0.01	N.D	N.D	0.05	N.D	0.01	59.05
815	Heritage Flakes	Grain Products	0.94	cluster3	52.08	0.54	0.40	1.29	1.43	0.03	0.01	0.01	0.01	N.D	N.D	0.10	N.D	0.02	55.91

816	Autumn Wheat Whole Wheat Biscuit	Grain Products	4.81	cluster3	56.74	0.80	3.88	0.95	5.02	0.03	0.01	0.01	0.00	N.D	N.D	0.12	N.D	0.03	67.58
817	All purpose flour	Grain Products	9.09	cluster3	59.33	0.17	0.55	0.70	0.74	0.03	0.00	0.00	0.00	N.D	N.D	0.15	N.D	0.01	61.67
818	Veggie crisps	Grain Products	2.43	cluster3	61.68	0.84	0.47	0.15	0.83	0.02	0.03	0.01	0.04	0.00	N.D	0.07	N.D	0.05	64.18
819	Aged white cheddar rice and corn puffs	Grain Products	2.94	cluster3	53.81	1.05	0.41	0.11	0.26	0.01	0.00	0.01	0.01	0.00	N.D	0.05	N.D	0.02	55.72
820	Chicken strips	Meat, Poultry, Fish, and Mixtures	37.39	cluster3	59.02	0.46	0.36	0.68	0.70	0.02	0.00	0.01	0.01	0.00	N.D	0.15	N.D	0.10	61.50
821	Crunchy breaded fish fillet	Meat, Poultry, Fish, and Mixtures	36.76	cluster3	56.55	0.84	0.37	0.58	0.75	0.02	0.00	0.00	0.00	0.00	N.D	0.12	N.D	0.04	59.28
822	Crispy battered fish fillet	Meat, Poultry, Fish, and Mixtures	27.16	cluster3	52.27	0.66	0.16	0.23	0.34	0.02	0.00	0.00	0.01	0.00	N.D	0.08	N.D	0.07	53.84
823	Rice cereal	Grain Products	0.24	cluster4	82.31	0.37	0.18	0.37	0.40	N.D	0.01	N.D	0.00	N.D	N.D	N.D	0.03	N.D	83.67
824	Single grain oatmeal cereal (Gerber)	Grain Products	3.44	cluster4	81.26	1.28	N.D	1.65	1.33	N.D	0.01	N.D	0.05	N.D	N.D	0.48	N.D	N.D	86.05
825	Rice cereal (Gerber)	Grain Products	4.35	cluster4	76.74	0.19	0.55	0.05	0.36	0.03	0.00	0.00	0.01	N.D	N.D	0.06	N.D	0.04	78.04
826	Puffed corn cereal	Grain Products	2.33	cluster4	75.29	0.14	N.D	0.20	0.32	N.D	0.01	N.D	0.03	N.D	N.D	N.D	N.D	0.02	76.01
827	Crisp rice receal	Grain Products	2.19	cluster4	75.49	0.28	1.80	0.05	0.27	0.03	0.00	0.00	N.D	N.D	N.D	0.03	N.D	0.03	77.99
828	Corn Starch	Grain Products	6.00	cluster4	89.80	0.34	0.69	0.00	0.10	0.03	0.00	0.00	0.00	N.D	N.D	0.02	N.D	0.00	90.99

*The fructose value for table sugar was corrected for degradation during hydrolysis by an empirical factor.
